# Likelihood-based random-effects meta-analysis with few studies: empirical and simulation studies

**DOI:** 10.1186/s12874-018-0618-3

**Published:** 2019-01-11

**Authors:** Svenja E. Seide, Christian Röver, Tim Friede

**Affiliations:** 1 Department of Medical Statistics, University Medical Center Göttingen, Humboldtallee 32, Göttingen, 37073 Germany; 2 Institute of Medical Biometry and Informatics, Heidelberg University Hospital, Im Neuenheimer Feld 130.3, Heidelberg, 69120 Germany

**Keywords:** Random-effects meta-analysis, Normal-normal hierarchical model (NNHM), Hartung-Knapp-Sidik-Jonkman (HKSJ) adjustment, Generalized linear mixed model (GLMM), Count data

## Abstract

**Background:**

Standard random-effects meta-analysis methods perform poorly when applied to few studies only. Such settings however are commonly encountered in practice. It is unclear, whether or to what extent small-sample-size behaviour can be improved by more sophisticated modeling.

**Methods:**

We consider likelihood-based methods, the DerSimonian-Laird approach, Empirical Bayes, several adjustment methods and a fully Bayesian approach. Confidence intervals are based on a normal approximation, or on adjustments based on the Student-*t*-distribution. In addition, a linear mixed model and two generalized linear mixed models (GLMMs) assuming binomial or Poisson distributed numbers of events per study arm are considered for pairwise binary meta-analyses. We extract an empirical data set of 40 meta-analyses from recent reviews published by the German Institute for Quality and Efficiency in Health Care (IQWiG). Methods are then compared empirically as well as in a simulation study, based on few studies, imbalanced study sizes, and considering odds-ratio (OR) and risk ratio (RR) effect sizes. Coverage probabilities and interval widths for the combined effect estimate are evaluated to compare the different approaches.

**Results:**

Empirically, a majority of the identified meta-analyses include only 2 studies. Variation of methods or effect measures affects the estimation results. In the simulation study, coverage probability is, in the presence of heterogeneity and few studies, mostly below the nominal level for all frequentist methods based on normal approximation, in particular when sizes in meta-analyses are not balanced, but improve when confidence intervals are adjusted. Bayesian methods result in better coverage than the frequentist methods with normal approximation in all scenarios, except for some cases of very large heterogeneity where the coverage is slightly lower. Credible intervals are empirically and in the simulation study wider than unadjusted confidence intervals, but considerably narrower than adjusted ones, with some exceptions when considering RRs and small numbers of patients per trial-arm. Confidence intervals based on the GLMMs are, in general, slightly narrower than those from other frequentist methods. Some methods turned out impractical due to frequent numerical problems.

**Conclusions:**

In the presence of between-study heterogeneity, especially with unbalanced study sizes, caution is needed in applying meta-analytical methods to few studies, as either coverage probabilities might be compromised, or intervals are inconclusively wide. Bayesian estimation with a sensibly chosen prior for between-trial heterogeneity may offer a promising compromise.

**Electronic supplementary material:**

The online version of this article (10.1186/s12874-018-0618-3) contains supplementary material, which is available to authorized users.

## Background

Meta-analyses of few studies are common in practice. For instance, a review of the Cochrane Library revealed that half of the meta-analyses reported in the Cochrane Library are conducted with two or three studies [1]. However, standard random-effects meta-analysis methods perform poorly when applied to few studies only [2, 3]. It is unclear, whether or to what extent small-sample-size behaviour can be improved by more sophisticated modeling. Bayesian random-effects meta-analyses with weakly informative priors for the between-study heterogeneity have been proposed for this setting [4] and their performance has been found to be satisfactory in numerical applications and simulations [3, 5]. Other alternative approaches including likelihood based methods have been mentioned as potential remedies [6].

In meta-analyses commonly a two-stage approach is applied. In the first step, data from the individual studies are analyzed resulting in effect estimates with standard errors. These are then combined in a second step. As individual patient data (IPD) are not generally available and effects with standard errors can typically extracted from publications, this two-stage approach makes a lot of sense from a practical point of view. With binary data, however, the individual patient data are summarized by 2 × 2 frequency tables and are usually readily available from publications [7]. Therefore, preference might be given to one-stage approaches in this setting over the commonly applied two-stage approach. However, numerical differences between the one-stage and two-stage approaches have been found to be small in a simple Gaussian model [8]. If differences are observed, these arise mostly for differing models [9, 10] or relate not to the main effects but interactions [11]. So, while a simpler two-stage model is often sufficient (especially in case of many studies and non-rare events), a one-stage model may on the other hand be expected to be more flexible and more exact [12]. A Bayesian approach may be more suitable especially in cases of few studies [3–5]. For a more detailed discussion of common models for binary data, see also Jackson et al. [13].

Although some model and method comparison studies appeared recently [13, 14], a systematic evaluation and comparison of the various methods is lacking in the context of few studies. Here we intend to close this gap by an empirical study and comprehensive simulations.

This manuscript is structured as follows. In the following section we summarize the meta-analysis approaches compared, the extraction of the empirical data set and the setup of the simulation study. Then the results of the empirical study as well as of the simulation study are presented. We close with a brief discussion and some conclusions.

## Methods

### Modeling approaches

In the following, we will consider meta-analyses based on binary endpoints, where each study’s outcome may be summarized in a 2 × 2 table giving the the numbers of participants with and without an event in both study arms.

### Normal-normal hierarchical model (NNHM)

#### Model specification

Traditionally, meta-analytical methods often follow a contrast-based summary measure approach which is based on the log-transformed empirical estimates of the outcome measure and their standard errors, and assuming an approximate normal likelihood [15].

In a common situation in random-effects meta-analysis, *k* independent studies are available in which the treatment effect *θ*_*i*_ is the parameter of interest (*i*=1,2,…,*k*). From each study, an effect estimate $\hat {\theta }_{i}$ with its estimated variance (squared standard error) $\sigma _{i}^{2}$ is provided for this treatment effect. It is then assumed that $\hat {\theta }_{i}$ follows a normal distribution centered around the unknown true treatment effect *θ*_*i*_, with the variance $\sigma _{i}^{2}$ accounting for the measurement uncertainty, or within-study variation. Although $\sigma _{i}^{2}$ usually only is an estimate, it is commonly treated as known. The *θ*_*i*_ may vary across study populations around a global mean *μ* due to the between-study heterogeneity *τ*. After integrating out the parameters *θ*_*i*_, the marginal model can be expressed as

1 $$  \hat{\theta}_{i} \;\sim\; \mathcal{N}\left(\mu, \,\sigma_{i}^{2} + \tau^{2}\right) \text{.}  $$

This model is commonly applied to both log-transformed risk ratio (RR) or odds ratio (OR) measures of treatment effect for binary data $\hat {\theta }_{i}$ [16, 17]; it is denoted as “model 1” in the investigation by Jackson et al. [13].

#### Inference

We will consider frequentist and Bayesian approaches to inference within the generic NNHM. In the frequentist approaches, an estimate of the between-study heterogeneity *τ* is usually required first. Different estimators are available; in the following we consider the commonly used DerSimonian-Laird (DL) [18], maximum likelihood (ML), restricted maximum likelihood (REML) [19, 20] and empirical Bayes (EB) estimators, the latter also being known as the Paule-Mandel estimator [21, 22]. Based on an estimate of this heterogeneity $\hat {\tau }$, the mean effect estimates are determined in a subsequent step by conditioning on the $\hat {\tau }$ value as if it were known.

The fully Bayesian estimation within the NNHM framework is done using three different prior specifications for the between-study heterogeneity (*τ*). Uncertainty in the heterogeneity is naturally accounted for when estimating the combined treatment effect *μ* by marginalisation. Especially if the number of studies is small, however, the choice of priors matters, as has been discussed by Turner et al. [23], Dias et al. [24, Sec. 6.2], or Röver [25]. We follow Friede et al. [3] and Spiegelhalter et al. [26, Sec. 5.7] and consider two half-normal priors with scales 0.5 and 1.0 for the between-study heterogeneity. These specifications include up to “*fairly high*” and “*fairly extreme*” heterogeneity [26, Sec. 5.7.3], and they also span the range of values considered in the simulations (see Table 1). In all of these approaches risk ratios (RR) and odds ratios (OR) can be used as the treatment effect.

**Table 1:** Absolute heterogeneity values (*τ*) corresponding to relative settings (*I*^2^) used in the simulations that are shown in Figs. 4 and 5

		Relative risk (RR)			Odds ratio (OR)		
	*I* ^2^	Equal	One small	One large	Equal	One small	One large
*k* = 2	0.25	0.0534	0.1254	0.0396	0.1781	0.4179	0.1321
	0.50	0.0926	0.2171	0.0687	0.3086	0.7237	0.2289
	0.75	0.1604	0.3761	0.1189	0.5345	1.2536	0.3964
	0.90	0.2777	0.6514	0.2060	0.9258	2.1712	0.6866
*k* = 3	0.25	0.0534	0.1069	0.0447	0.1781	0.3563	0.1491
	0.50	0.0926	0.1852	0.0775	0.3086	0.6172	0.2582
	0.75	0.1604	0.3207	0.1342	0.5345	1.0690	0.4472
	0.90	0.2777	0.5549	0.2324	0.9258	1.8516	0.7746
*k* = 5	0.25	0.0534	0.0844	0.0484	0.1781	0.2981	0.1613
	0.50	0.0926	0.1549	0.0838	0.3086	0.5164	0.2795
	0.75	0.1604	0.2683	0.1452	0.5345	0.8944	0.4840
	0.90	0.2777	0.4648	0.2515	0.9258	1.5491	0.8384

### Generalized linear mixed models (GLMM)

#### Models

The statistical model may also be based directly on the count data, using either a binomial or a Poisson assumption on the numbers of events per study arm. Generalized linear mixed models (GLMMs) may then be fitted to the data, using a logarithmic link for Poisson rates or a logit link for proportions. Treatment effects may be modeled based on ORs or RRs, and random effects may be included at several stages in order to account for heterogeneity. In addition, we also consider some approximate variants of these models. The models used are outlined briefly below; most of these are also discussed in more detail by Jackson et al. [13].

#### Model specification and inference

If a Poisson distribution is assumed for the number of events per arm and study, a log-link will be used to model the RR. Following Böhning et al. [7, Ch. 2] this model is estimated using the profile likelihood; in the following, this model will be denoted as the “PN-PL” model.

For binomially distributed numbers of events per study arm, a logit-link will be applied to model ORs in a logistic regression. Four different specifications are included in the comparison. Unconditional logistic regression with fixed and random study-specific nuisance parameters as discussed by Turner et al. [27] are considered (“UM.FS” and “UM.RS”, respectively, in the following). These correspond to models 4 and 5 in Jackson et al. [13].

In addition, we follow van Houwelingen et al. [28] in using a conditional logistic approach, where the total number of events per study is conditioned upon, in order to avoid the need to also model their variability [19]. The likelihood of this conditional model can be described using Fisher’s non-central hypergeometric distribution [28] (“CM.EL” in the following, and corresponding to model 7 in [13]).

Fisher’s non-central hypergeometric distribution may be approximated by a binomial distribution, if the number of cases is small compared to the overall participants in that study [29]; this model specification will be denoted by “CM.AL” in the following (approximate version of model 7 in [13, Sec. 3.7.2]). All of the logistic regression models are fitted using maximum likelihood.

### Confidence and credible intervals combined effects

The 95% credible intervals in the Bayesian estimation and confidence intervals in the frequentist approaches are estimated for the combined treatment effect *μ*. The narrowest 95% highest posterior density intervals are used in the Bayesian estimation. For the construction of confidence intervals, Wald-type intervals based on normal quantiles are considered, which are known to be anti-conservative when the number of studies is small or non-negligible amounts of heterogeneity are present [2, 3, 30, 31]. To account for this behaviour, confidence intervals are in addition constructed using Student’s t-distribution in case of the GLMMs, and the Hartung-Knapp-Sidik-Jonkman (HKSJ) adjustment [30–32] in case of the NNHM. The HKSJ-adjusted intervals tend to be wider than the Wald-type intervals, although this is not strictly the case [2, 32, 33]. Knapp and Hartung [33] proposed a modification of the Hartung-Knapp-Sidik-Jonkman adjustment (mHKSJ) correcting HKSJ-adjusted intervals in the cases where they are counterintuitively narrow. These modified confidence intervals are also considered.

### *I*^2^ as measure of between-study heterogeneity

The “relative amount of between-study heterogeneity” can be expressed in terms of the measure *I*^2^, which expresses the the between-study variance (*τ*^2^) in relation to the overall variance ($\tilde {\sigma }^{2}$) [34], which is stated as

2 $$ I^{2} = \frac{\hat{\tau}^{2}}{\tilde{\sigma}^{2} + \hat{\tau}^{2}} \text{.}  $$

In the calculation of *I*^2^, a “typical” $\tilde {\sigma }^{2}$ value is required as an estimate of the within-study variances $\sigma _{i}^{2}$. Higgins and Thompson [34] suggest a weighted average of the individual within-study variances as “typical” value. This, together with the fact that the *I*^2^ is bounded between zero and one, permits the interpretation of heterogeneity magnitude as a relative percentage. The *I*^2^ is used to set the amount of heterogeneity in the simulation study. Hoaglin [35] remarks that the probability for observing a moderate (estimated) *I*^2^ even in the absence of heterogeneity is dependent on the number of studies included and is not negligible. As the *I*^2^ expresses the between-study variation relative to the total variation, the same values of *τ* may lead to different values of *I*^2^, depending on the precision of the underlying studies and should therefore always be interpreted as a relative measure [36].

### Extraction of the empirical data set

A data set of 40 meta-analyses was extracted from publications of the German Institute for Quality and Efficiency in Health Care (IQWiG). IQWiG publications were searched chronologically for meta-analyses of binary data in April 2017 starting with the most recent available ones and reaching back to March 2012. In total, 521 documents were screened, including all document types in the search. If a detailed and a short version of a document existed, only the detailed version was considered. From documents including at least one meta-analysis of binary data, the first one was extracted to obtain a realistic data set with respect to the number of studies typically included in a meta-analysis and the sample sizes of those studies. Meta-analyses involving studies with zero events in one or more arms were excluded from the data set for better comparability of the evaluated methods.

### Simulation procedure

**Fig. 1:**
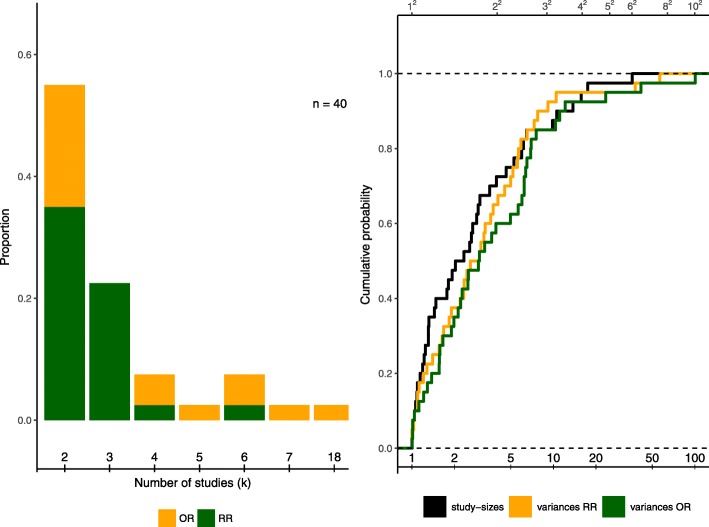
Characteristics of the data set extracted from IQWiG publications. Left side: Proportions of number of studies included per meta-analysis out of *n* = 40. Colours indicate the effect measure used in the original publications. Right side: Empirical distribution function for the proportion of study sizes (largest vs. smallest per meta-analysis, black) and the proportion of study-specific variances (largest vs. smallest per meta-analysis) for the log-transformed RR (green) and the log-transformed OR (orange). All meta-analyses are included for both effect measures

To compare properties of the investigated approaches to meta-analysis, we conducted a Monte-Carlo simulation adapting the setup from IntHout et al. [14] who described the simulation of 2 × 2 tables. In deviation from IntHout et al. [14], series of trials with up to 10 studies were simulated, and each series was repeated only 2000 times. Three different designs were considered, where in the first one all studies were of equal size, one study was ten times larger than the other studies in the second, and one study was only a tenth of the size of the other studies in the third design. It should be noted however that this ratio corresponds to extreme, but not unrealistic cases, as is also illustrated in the right panel of Fig. 1. The (less common) case of equal sizes is of interest here, as this is where we expect the HKSJ methods to perform best [2, 14].

**Fig. 2:**
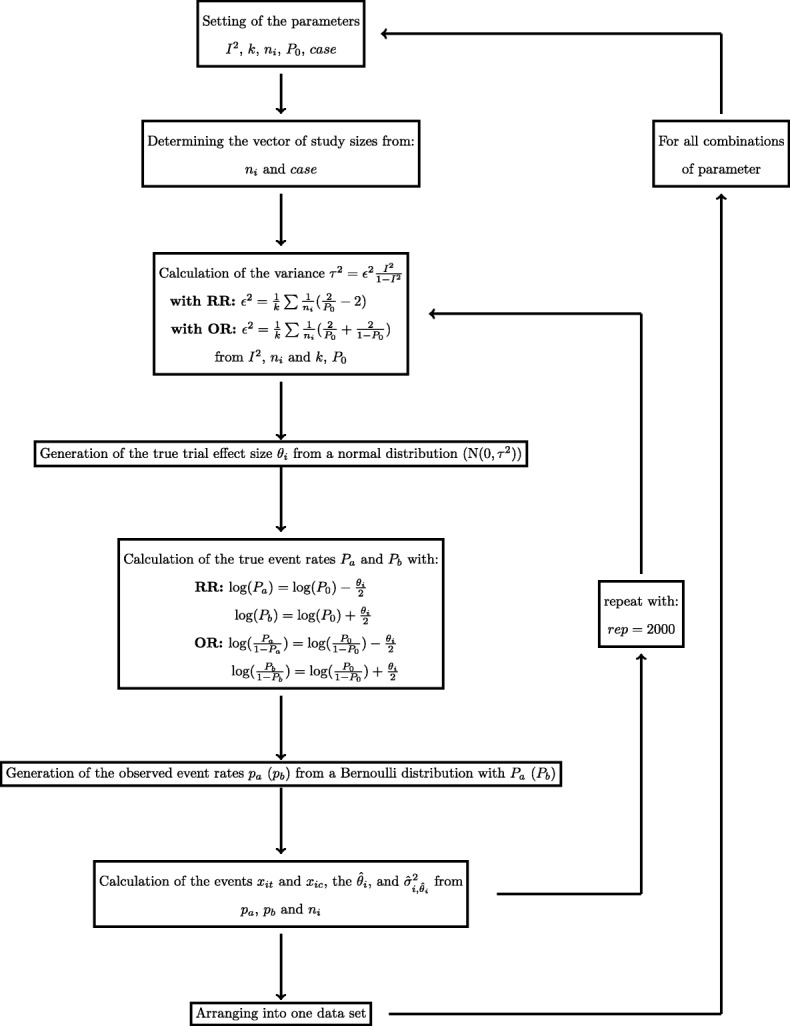
Generation of data sets in the simulation study

To generate dichotomous outcomes, *p*_0_ and *I*^2^ have to be set in advance. Considered values of the *I*^2^ correspond to levels of no, low, moderate, high and very high heterogeneity, respectively [37]. Note however, that the same *I*^2^ value may correspond to different values of between-study heterogeneity *τ* depending on the effect measure used, and on whether or not study sizes are balanced; the resulting *τ* values are shown in Table 1. From the *τ* values one can see that in some of the scenarios, the *I*^2^ settings imply unrealistically large absolute heterogeneity [26, Sec. 5.7.3], which needs to be considered in the interpretation. This would be true for instance for odds ratios with *I*^2^ in the range of 0.75 and 0.90 and one small study when *τ* is roughly in the range of 1 to 2 (see Table 1). The baseline event rate (*p*_0_) needs to be set as an additional parameter and varies from 0.1 to 0.9 in steps of 0.2. The treatment effect *θ*_*i*_ is set to unity for both RR and OR, which corresponds to the absence of an effect. Note that while for meta-analyses of continuous (or, more specifically, normally distributed) endpoints the magnitude of the simulated treatment effect (*θ*_*i*_) should not affect performance, e.g. for binomial counts it may make a difference, as it affects the chances of observing few or zero events in the treatment arm. However, since we chose not to focus on rare-event issues, and in order to keep the number of simulation scenarios manageable, only the case of *no effect* was investigated. For every combination of the simulation parameters 2000 repetitions are simulated. In case zero event counts occurred, for the models based on the NNHM, a continuity correction of 0.5 was added to all cells of the affected study’s contingency table. Zero counts, however, were rare in the scenarios considered. The simulation scenarios are also summarized in Table 2. For more details on the simulation procedure see also IntHout et al. [14] and Fig. 2 below. As in the case of the empirical data set, the two-sided significance level *α* was set to 0.05. Different methods and scenarios are compared based on observed confidence or credible interval coverage probabilities and lengths.

**Table 2:** Parameters of the simulation for both effect measures, i.e., relative risk and odds ratio

Parameter	Values
Effect measure (*θ*_*i*_)	RR, OR
Design	Equally sized studies,
	One small study ($\frac {1}{10}$ size)
	One large study (10-fold size)
Observations per study arm (*n*_*i*_)	25, 50, 100, 250, 500, 1000
Number of studies (*k*)	2, 3, 5, 10
Event rates (*p*_0_)	0.1, 0.3, 0.5, 0.7, 0.9
Level of heterogeneity (*I*^2^)	No heterogeneity: 0.00
	Low heterogeneity: 0.25
	Moderate heterogeneity: 0.50
	High heterogeneity: 0.75
	Very high heterogeneity: 0.90

### Estimation in R

The software environment R [38] and two of its extensions, the metafor [39, 40] and bayesmeta [25, 41] packages are used with their default options. As no implementation in R was found for the PL estimation of Poisson-normal model we translated the steps described by Böhning et al. [7, Ch. 2] into R code which is shown in the Additional file 1.

## Results

### Empirical study

Most (419; 80%) of the 521 documents searched did not include a meta-analysis, because either the assignment was canceled (11; 2%), the assignment had just started without results being available at the time of search (70; 13%), no meta-analysis was included or accepted by the IQWiG (186; 36%), no study (34; 7%) or just one study (118; 23%) was identified. Out of the remaining 102 documents which included at least one meta-analysis, 25 (5%) did not include any binary meta-analysis, 19 (4%) were network meta-analyses, and in 18 (3%) cases the first binary meta-analysis included at least one study with zero events. An overview over the identified meta-analyses is given in Table 3; the data are also available online [42].

**Table 3:** Data extracted from IQWiG publications [42]

No.	Identifier	Date	Endpoint	Page	Number of studies (*k*)	Effect measure
1	N15-06	2017-03	Morning pain	85	5	OR
2	N15-11	2017-03	Ear infection	62	2	OR
3	S15-02	2017-01	Mortality	53	2	OR
4	D15-02	2017-01	Mortality	74	2	OR
5	A16-71	2016-12	Morbidity	5	6	OR
6	A16-38	2016-12	Vomiting	4	2	RR
7	P14-03	2016-11	Breast cancer screening	55	3	RR
8	N14-02	2016-08	Remission from anxiety disorder	127	2	OR
9	A16-30	2016-08	AIDS-defining events	103	2	RR
10	N15-07	2016-08	Ejaculation dysfunction	89	4	OR
11	A16-11	2016-06	Serious adverse events	86	2	RR
12	A10-03	2016-04	Serious adverse events	89	2	OR
13	A15-57	2016-02	St. George’s respiratory questionnaire response	22	2	RR
14	A15-45	2016-01	Morbidity	24	2	OR
15	A15-31	2015-11	Mortality	87	2	RR
16	A15-25	2015-10	Serious adverse events	89	2	RR
17	A15-21	2015-07	Mortality	16	2	RR
18	S13-04	2015-05	Screening for abdominal aortic aneurysm	71	4	OR
19	A15-06	2015-05	Morbidity	96	3	RR
20	A15-05	2015-03	Morbidity	4	2	RR
21	A14-38	2015-01	Serious adverse events	65	3	RR
22	A14-25	2014-11	Serious adverse events	115	2	RR
23	A14-22	2014-10	Transition Dyspnea Index responder	67	2	RR
24	A14-19	2014-09	Urge to urinate	75	3	RR
25	A14-18	2014-09	Persistent virological response (SVR24)	194	3	RR
26	S13-03	2014-06	Participants with cervical intraepithelial neoplasia 3+	15	6	RR
27	A13-29	2013-10	Metformidosis	15	3	RR
28	A10-01	2013-08	Remissions	1183	2	OR
29	A13-20	2013-08	Visual acuity	28	3	RR
30	S11-01	2013-07	Bowel cancer	61	7	OR
31	A13-23	2013-06	Mortality	15	2	RR
32	A13-05	2013-04	Full recovery	19	4	RR
33	A05-10	2013-04	Cardiovascular death	75	3	RR
34	A12-19	2013-03	Ocular adverse event	17	2	RR
35	A05-18	2012-08	Serious adverse events	67	18	OR
36	A12-10	2012-07	Adverse events	20	3	RR
37	A12-03	2012-04	Loss of transplant	23	2	RR
38	A12-04	2012-04	Virus occurrence	22	3	RR
39	A09-05	2012-04	Alzheimer’s disease assessment scale	51	6	OR
40	A11-30	2012-03	Mortality	24	2	OR

**Table 4:** Abbreviations used for analysis models

NN-DL	Normal-normal (NN) model using the DerSimonian-Laird (DL) heterogeneinty estimator
NN-REML	NN model using the restricted maximum-likelihood (REML) estimator
NN-EB	NN model using the empirical-Bayes (EB) estimator
PN-PL	Poisson model using profile likelihood (PL) estimation
BN-UM.FS	Binomial model using unconditional logistic regression and fixed study (nuisance) parameters
BN-UM.RS	Binomial model using unconditional logistic regression and random study (nuisance) parameters
BN-CM.EL	Conditional (hypergeometric) model (exact likelihood)
BN-CM.AL	Conditional (hypergeometric) model (approximate likelihood)
NN-Bayes HN(0.5)	NN Bayesian model using a half-normal heterogeneity prior with scale 0.5
NN-Bayes HN(1.0)	NN Bayesian model using a half-normal heterogeneity prior with scale 1.0

In the original publications, a slight majority of studies (26 of 40) was analyzed using RR as the effect measure. In the extracted data set, 21 out of the 40 meta-analyses (53%) included only 2 studies, while 10 (25%) consisted of three studies. Even in this small example, a common occurrence of 2- and 3-study meta-analyses is found, which is also observed empirically by [43] and [44]. The distribution of study sizes and endpoints is also illustrated on the left panel in Fig. 1. With only two studies included, three methods coincide: the DL, the REML and the EB estimation [45]. As this is the case for a major share of the data set, these three methods are expected to show similar results in the analysis. The maximum number of studies observed is 18. The original analyses were based on the NNHM, and, with only the exceptions of the publications A15-45, S11-01 and A11-30 performed using DL variance estimation.

Imbalance in study sizes may influence the estimation of an overall treatment effect [2, 14, 46]. As IntHout et al. [14] observe in an empirical study, such unequal study sizes are common in meta-analyses. In the data set extracted from IQWiG publications, ratios of sample sizes between the largest and the smallest study in a meta-analysis ranged from 1.0 up to 15.8, with a mean of 3.4 and a median of 1.9. Nearly half of the meta-analyses included at least one study twice as large as the smallest study. In the NNHM, study-specific variances $\sigma _{i}^{2}$ should roughly be inversely proportional to sample sizes; imbalances in sample size then affect analysis via an imbalance in the *σ*_*i*_. Ratios of largest to smallest study sizes and variances using both effect measures for all studies are shown on the right panel in Fig. 1, where the ratio between the largest and the smallest value is ordered by the ratio of sample sizes in descending order. It can be observed that the ratios of the variances of ORs seem to vary more when study sizes are unbalanced than those of the RRs. However, they both roughly follow the same pattern as the ratio of study sizes.

**Fig. 3:**
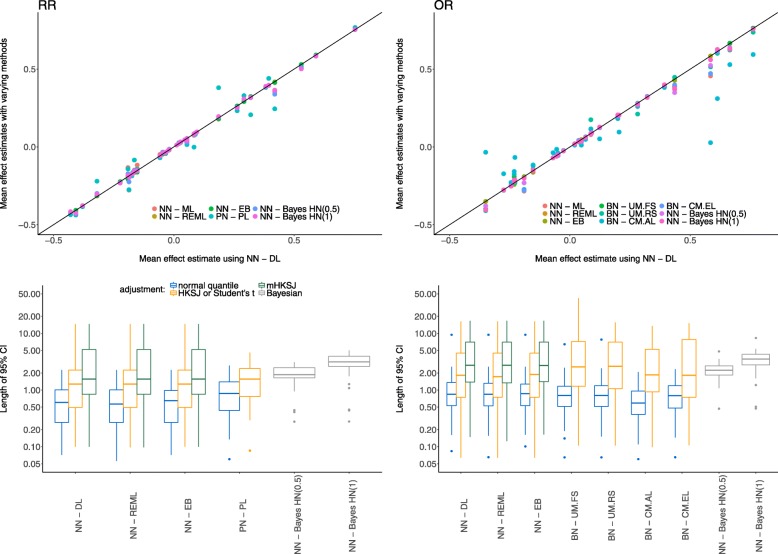
Estimates of the combined treatment effect and lengths of confidence or credible intervals for both effect measures, empirical data set. The first row shows the treatment effect estimates for the RR (left column) and the OR (right column) compared to the standard DL approach. Colours indicate the various methods. The second row illustrates the length of confidence or credible intervals and the respective adjustments, again for the RR (left column) and the OR (right column)

The extracted data set is then analyzed based on the models and methods described above. As 2 × 2 tables are available for all studies, both effect measures are used to summarize the individual meta-analyses and to evaluate the influence of the choice of effect measure on the estimation results. The ratios of point estimates of the different methods against the standard DL approach are illustrated by the first row of Fig. 3 where the RR is displayed in the left and the OR in the right panel. As expected, DL, REML and EB estimation coincide in the majority of cases including only two studies [45]. These three estimators are also observed to behave comparable when more than two studies are included, as do the point estimates of the Bayesian approach. The greatest deviation from the standard DL approach is observed in the GLMMs in both effect measures. In the case of OR as an effect measure, UM.FS and UM.RS perform comparable. CM.EL estimation does not converge in all cases, however, in the cases where convergence was achieved, it is in line with DL estimation. The CM.AL however, is in general different from the DL estimation. The Poisson-based results also differ considerably from the DL estimates.

The length of confidence intervals for the frequentist and credible intervals in the Bayesian estimation are also of importance as it might not be possible to detect significant treatment effects if intervals are inconclusively wide. For both effect measures, all intervals and the discussed adjustments are shown in the second row in Fig. 3. Again, the RR is displayed in the left and the OR in the right panel. The Bayesian credible intervals are generally wider than the unadjusted confidence intervals and more similar to the adjusted ones with respect to the median length, but exhibiting less variability.

### Simulation study

**Fig. 4:**
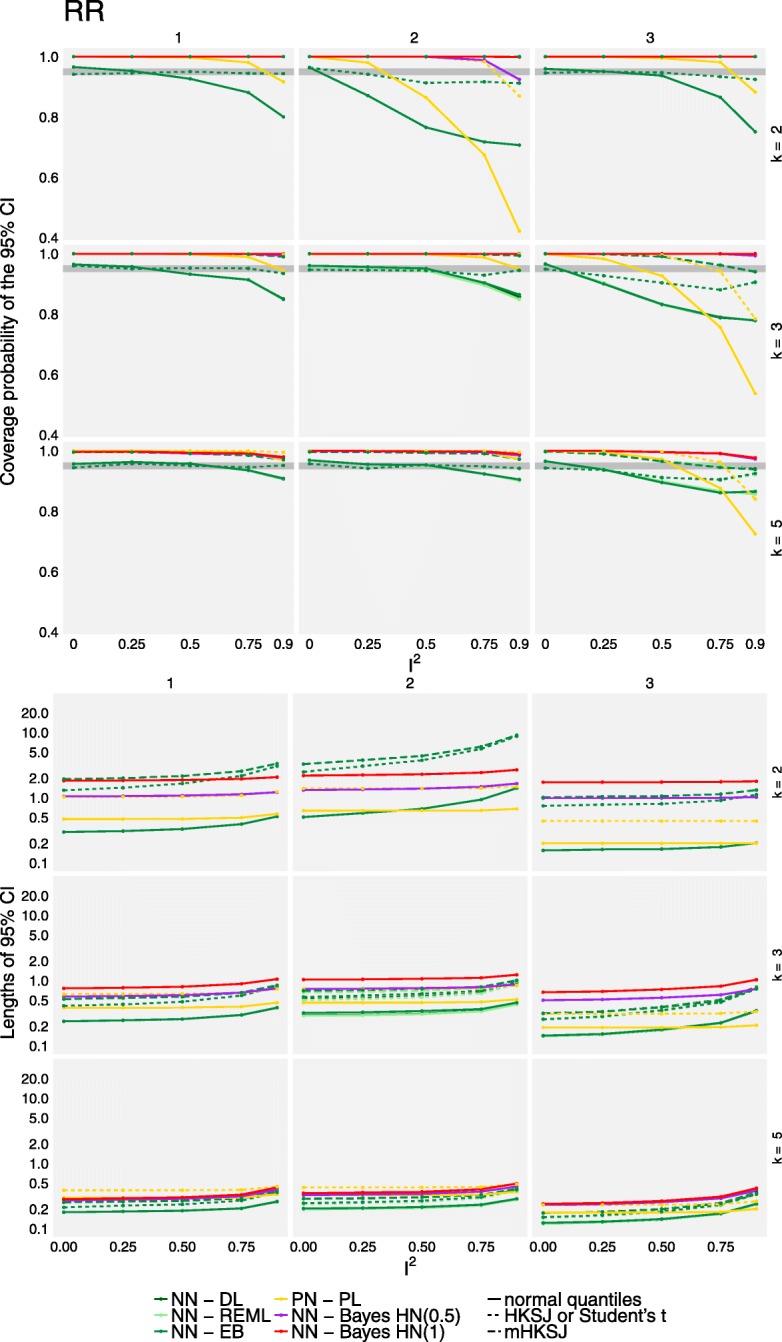
Coverage probabilities and lengths of 95% confidence or credible intervals for the overall effect for RR effects based on the simulated data. The top panel shows the coverage probabilities of treatment effect CIs for the different methods (colours) and adjustments (line types). The grey area indicates the range expected with 95% probability if the coverage is accurate. The bottom panel similarly shows the lengths of 95% confidence or credible intervals. Results are illustrated for a study size of *n*_*i*_ = 100 and a baseline event probability *p*_0_ = 0.7, and are based on 2000 replications per scenario. CM.EL is omitted due to low convergence rates

**Fig. 5:**
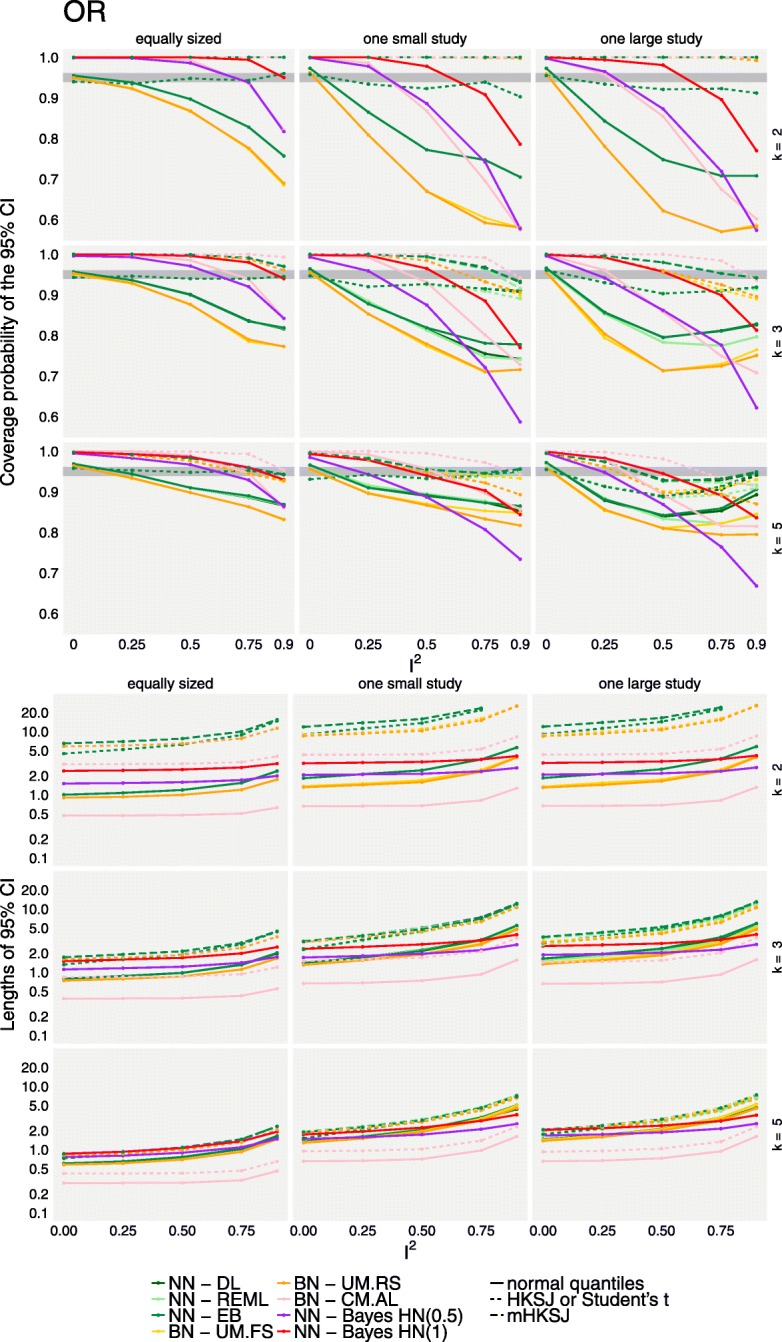
Coverage probabilities and lengths of 95% confidence or credible intervals for the overall effect for OR effects based on the simulated data. The top panel shows the coverage probabilities of treatment effect CIs for the different methods (colours) and adjustments (line types). The grey area indicates the range expected with 95% probability if the coverage is accurate. The bottom panel similarly shows the lengths of 95% confidence or credible intervals. Results are illustrated for a study size of *n*_*i*_ = 100 and a baseline event probability *p*_0_ = 0.7, and are based on 2000 replications per scenario. CM.EL is omitted due to low convergence rates

Figures 4 and 5 illustrate the coverage rates (first row) and lengths (second row) of the 95% confidence or credible intervals of the different methods for the relative risks and odds ratios, respectively. All results shown here exemplarily refer to the combination of 100 participants per arm and study and a baseline event rate of 0.7. Results of the other scenarios may be found in the supplement (see Additional files 2 and 3). The different methods are indicated by colours, while the different adjustments are indicated by the line type.

Non-convergence rates averaged over all scenarios and both effect measures are mostly negligible in the methods based on the normal likelihood on the log-scale (ML: 0.049%, EB: 0.032%, HN(1.0): 0.002%, HN(0.5): 0.036%). Estimation based on REML or the methods taking the distributional assumptions on the trial-arms lead to slightly higher non-convergence rates (REML: 0.43%, UM.FS: 0.43%, UM.RS: 0.22%, CM.AL: 0.47%). The only method with high non-convergence rates is CM.EL, with an average of 18%. None of the methods fully dominates the others over the range of the investigated scenarios. Estimation using CM.EL for the binomial-normal model however was computationally expensive and convergence was problematic in a large proportion of scenarios (using the default values), as has been noted before [13, 19]; these results are omitted here. Coverage rates of all methods are comparable when either the number of studies included in each meta-analysis is sufficiently large or when the heterogeneity is absent or low (*I*^2^ ≤ 0.25). However, given the frequency with which 2- or 3-study meta-analyses occur empirically in our example data set and others [43, 44] and the difficulties in the determination of the absence of heterogeneity [2] this is hardly relevant in practice. In general when study sizes were not balanced, coverage rates for all methods were substantially lower even in the presence of only low heterogeneity in the simulation of OR, while Bayesian estimation and the adjustment of frequentist confidence intervals resulted in better coverage when RR was used. This might be due to the *I*^2^ values translating to lower values of absolute heterogeneity in the latter case. In the presence of heterogeneity, coverage could drop as low as 40% for some extreme scenarios in both, frequentist and Bayesian estimation, resulting in high false-positive rates. In general, it was also observed that one large study tended to lead to lower coverage than one small study per meta-analysis in the frequentist methods when more than *k* = 2 studies are present, which is in line with [14]. This effect is more noticeable in the unadjusted methods, in scenarios where the number of patients per arm (*n*_*i*_) is small, or when heterogeneity (*I*^2^) is large. When considering *k* = 2 trials per meta-analysis, coverages are comparable (OR) or the effect is even reversed (RR). The frequentist methods based on the normal-normal hierarchical model perform similarly, or, in the case of two studies per meta-analysis, even identically [45]. In the case of heterogeneous data, in particular regarding small study sizes, all frequentist methods perform below the nominal coverage probability when confidence intervals are not adjusted. In the scenarios with unbalanced study sizes this is even more pronounced than in the balanced scenarios; this is in line with the findings of [14] and [2]. Coverage can, at the cost of interval width, be increased by either using the HKSJ or the mHKSJ adjustment, but the HKSJ adjustment yields in some scenarios coverage probabilities which are still below the nominal level [14].

The length of confidence and credible intervals is illustrated in the second row of Figs. 4 and 5. Bayesian credible intervals are, as in the case for the empirical data set, in general wider than the unadjusted confidence intervals from the frequentist estimations. When compared to adjusted confidence intervals, Bayesian credible intervals tend to be, especially in the presence of heterogeneity and with only two studies per meta-analysis, narrower than the frequentist intervals based on the normal-normal model as long as the number of patients per trial arm is small. However, when *n*_*i*_ increases, this is no longer true for RR (in contrast to the scenarios using OR). These differences may be due to the fact that idential *I*^2^ settings can imply very different (and sometimes possibly unrealistically large) magnitudes of heterogeneity values on the *τ* scale, as can also be seen in Table 1. In these extreme scenarios, adjusted frequentist confidence intervals are observed to be inconclusively wide, with the exception of BN-UM.FS and BN-UM.RS, and especially when estimation is based on the NNHM. In the other scenarios, Bayesian credible and adjusted confidence intervals are comparable.

## Discussion

In our empirical study we found that the majority of the 40 meta-analyses extracted from publications of IQWiG included only two studies. This is in agreement with a much larger empirical investigation based on the Cochrane Library by Turner et al. [1]. This finding emphasizes the need for methods appropriate for meta-analysis with few studies. Furthermore, varying methods and / or effect measures lead to differences in the results for the 40 meta-analyses considered. This demonstrates that prespecification of methods as well as effect measures is important for controlling operating characteristics. The problems encountered in meta-analyses of few studies may mostly be attributed to the estimation of heterogeneity, and in particular to the proper accounting for its uncertainty in constructing intervals for the combined effect. The difference in performance between different heterogeneity estimators is relatively small compared to the difference in whether or how heterogeneity uncertainty is propagated through to the effect estimate [3].

In the simulation study, coverage probability was below the nominal level for all frequentist methods in the presence of heterogeneity and few studies. This phenomenon is even more pronounced when studies included in a meta-analysis are of unequal size. However, coverage probabilities generally improve when confidence intervals are adjusted based on the Student-*t*-distribution. Bayesian methods mostly result in better coverage across all scenarios, except for some cases of very large heterogeneity (in terms of *τ*) where the coverage is slightly lower. Credible intervals are empirically and in the simulation study wider than unadjusted confidence intervals, but considerably narrower than adjusted ones, with some exceptions when considering RRs and large numbers of patients per trial-arm. Previous simulation studies comparing a more restricted set of methods including standard frequentist and Bayesian approaches only led to similar conclusions. The simulations presented here considering a wider set of methods show that the issues entailed by the increased complexity of some likelihood-based approaches may often outweigh their expected advantages [6]. However, confidence intervals based on the GLMMs for example are in general slightly narrower than those from other frequentist methods. Furthermore, certain maximum-likelihood methods turned out to suffer from frequent numerical problems in the setting with few studies. To our knowledge, this has not been described previously.

Our empirical investigation did not consider all IQWiG reports, but only the most recent 40 meta-analyses at the time of extraction. A consideration of all meta-analyses might have led to a more complete picture, but was not feasible with the resources of this project as no specific funding was available. Furthermore, the simulation study could have been enriched by additional methods. For instance, we only considered Bayesian two-stage approaches but did not include Bayesian approaches utilizing the full information of the 2 × 2 tables. The latter was considered recently by [47] in the context of network meta-analyses, where pairwise meta-analysis would be a special case. As for the likelihood methods, we would expect that the results of the one-stage approach are overall quite similar to those of the two-stage approach considered, maybe with the potential of some small improvements. As discussed in the context of the simulation setup, a pre-specified *I*^2^ value may correspond to rather different *τ* values, depending on the circumstances (see also Table 1). Consequently, one may generally expect larger *I*^2^ values for log-RR endpoints, and smaller *I*^2^ values for log-OR endpoints, while heterogeneity priors are probably best discussed at the scale of *τ* values (a prior specification in terms of *I*^2^ would be possible [25], but this would be hard to motivate). By relating the heterogeneity to the *τ* value, the question to consider is *by what factor* the true RRs or ORs *θ*_*i*_ are expected to differ solely due to between-trial heterogeneity [26, Sec. 5.7.3], and the reasonably expected range should then be covered by the prior. For example, a heterogeneity of *τ* = 1.0 implies that the central 95% of true study means (*θ*_*i*_) span a range of a factor of 50 [3, 26]. The HN(0.5)-prior confines *τ* to values below 1.0 with roughly 95% probability, while the HN(1.0)-prior constitutes a conservative variation that instead allows for twice as large heterogeneity, implying a plausible range of roughly up to factor of 50^2^=2500.

The limits of applicability of approximate meta-analysis methods have been discussed from the perspective of the NNHM by Jackson and White [48]. In the limit of many studies (large *k*) and large sample sizes (large *n*_*i*_), the normal approximation usually works well. It starts breaking down, however, when the number of studies (*k*) gets too small. The problem then is related to the estimation of heterogeneity (*τ*) and proper accounting for the associated uncertainty; inference would still be exact if the heterogeneity was known. In the frequentist context, use of the HKSJ adjustment helps, especially if the study-specific standard errors are roughly balanced [49]. This is not so much of a problem when Bayesian methods along with reasonable priors are used; these methods yield valid inference irrespective of the number of included studies [50]. Problems also arise when events are rare or sample sizes (*n*_*i*_) are small. In either case, the chances of observing few or no events in a treatment group increase, and normal approximations to the likelihood break down. In such situations, a solution might be to resort to exact likelihoods respecting the discrete nature of the data, for example a GLMM, which may again be done in frequentist or Bayesian frameworks [7, 40, 51].

## Conclusions

In the presence of between-study heterogeneity, especially with unbalanced study sizes, caution is needed in applying meta-analytical methods to few studies, as either coverage probabilities of intervals may be compromised, or they may be inconclusively wide. Bayesian estimation with sensibly chosen prior for the between-study heterogeneity may offer a compromise and promising alternative.

## Additional files

Additional file 1 LikelihoodMA-PoissonPL.R: The R code implementing profile likelihood (PL) estimation for the Poisson model according to Böhning et al. [7, Ch. 2] (see also the “Methods” section). (R 4 kb)

Additional file 2 Supplement-1.pdf: The plots analogous to Fig. 4, for *all* simulation scenarios. (PDF 676 kb)

Additional file 3 Supplement-2.pdf: The plots analogous to Fig. 5, for *all* simulation scenarios. (PDF 826 kb)

## Abbreviations

For abbreviations of model variations used,: see also Table 4.
DL: DerSimonian-Laird
EB: Empirical Bayes
GLMM: Generalized linear mixed model
HKSJ: Hartung-Knapp-Sidik-Jonkman
IPD: Individual patient data
IQWiG: Institut für Qualität und Wirtschaftlichkeit im Gesundheitswesen (Institute for Quality and Efficiency in Health Care)
mHKSJ: Modified HKSJ
ML: Maximum likelihood
NNHM: Normal-normal hierarchical model
OR: Odds ratio
PL: Profile likelihood
REML: Restricted ML
RR: Risk ratio
