# Supplementary material for: Likelihood-based random-effects meta-analysis with few studies: empirical and simulation studies
Source: BMC Med Res Methodol. 2019 Jan 11;19:16. doi: 10.1186/s12874-018-0618-3 (PMC6330405; doi:10.1186/s12874-018-0618-3)
Supplement: Supplementary file 2 — Supplement-1.pdf: The plots analogous to Fig. 4, for all simulation scenarios. (PDF 676 kb) [file 12874_2018_618_MOESM2_ESM.pdf]

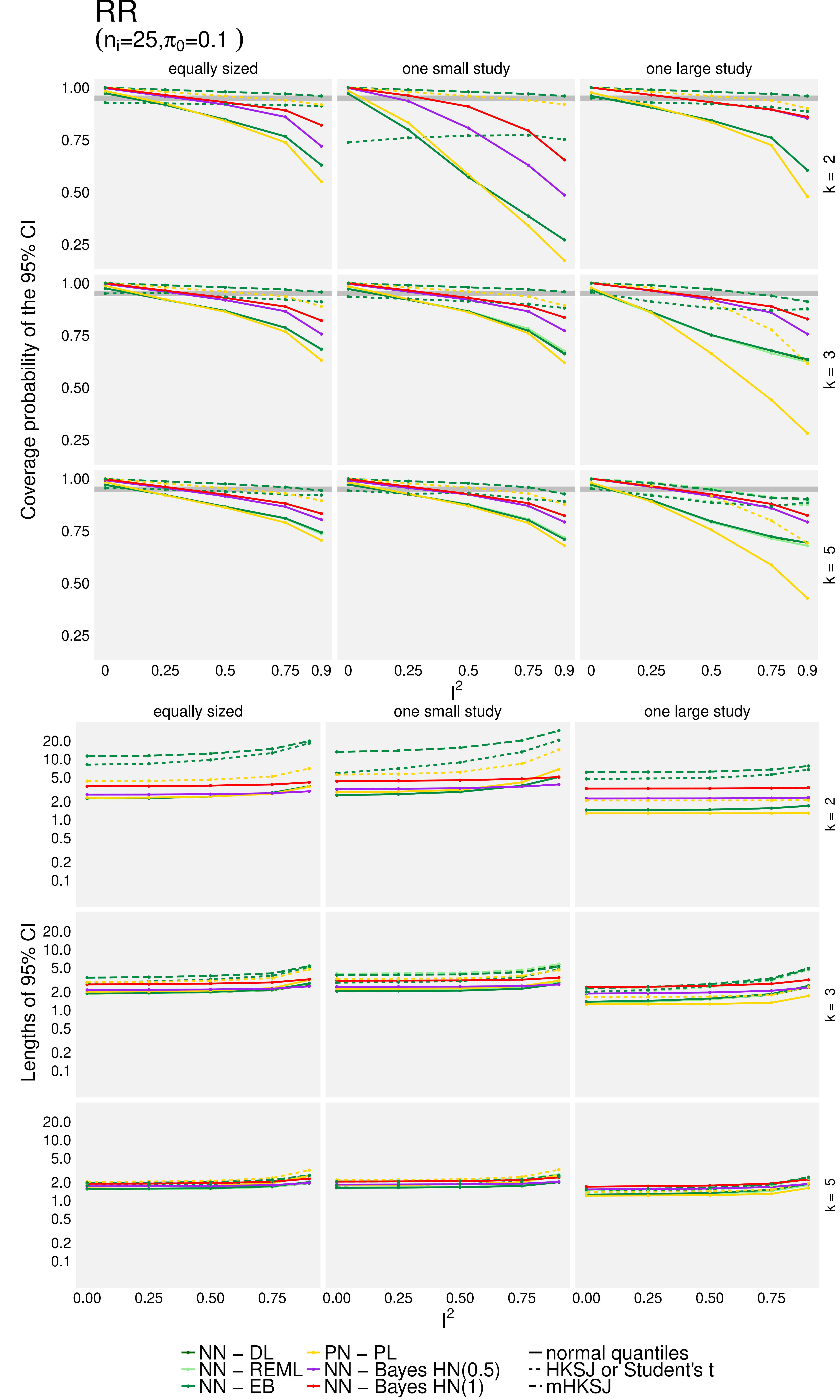
RR
(n_i=25,π_0=0.1)
equally sized
one small study
one large study
Coverage probability of the 95% CI
1.00
0.75
0.50
0.25
k = 2
k = 3
k = 5
0
0.25
0.5
0.75
0.9
I^2
Lengths of 95% CI
20.0
10.0
5.0
2.0
1.0
0.5
0.2
0.1
0.00
0.25
0.50
0.75
NN – DL
NN – REML
NN – EB
PN – PL
NN – Bayes HN(0.5)
NN – Bayes HN(1)
normal quantiles
HKSJ or Student's t
mHKSJ

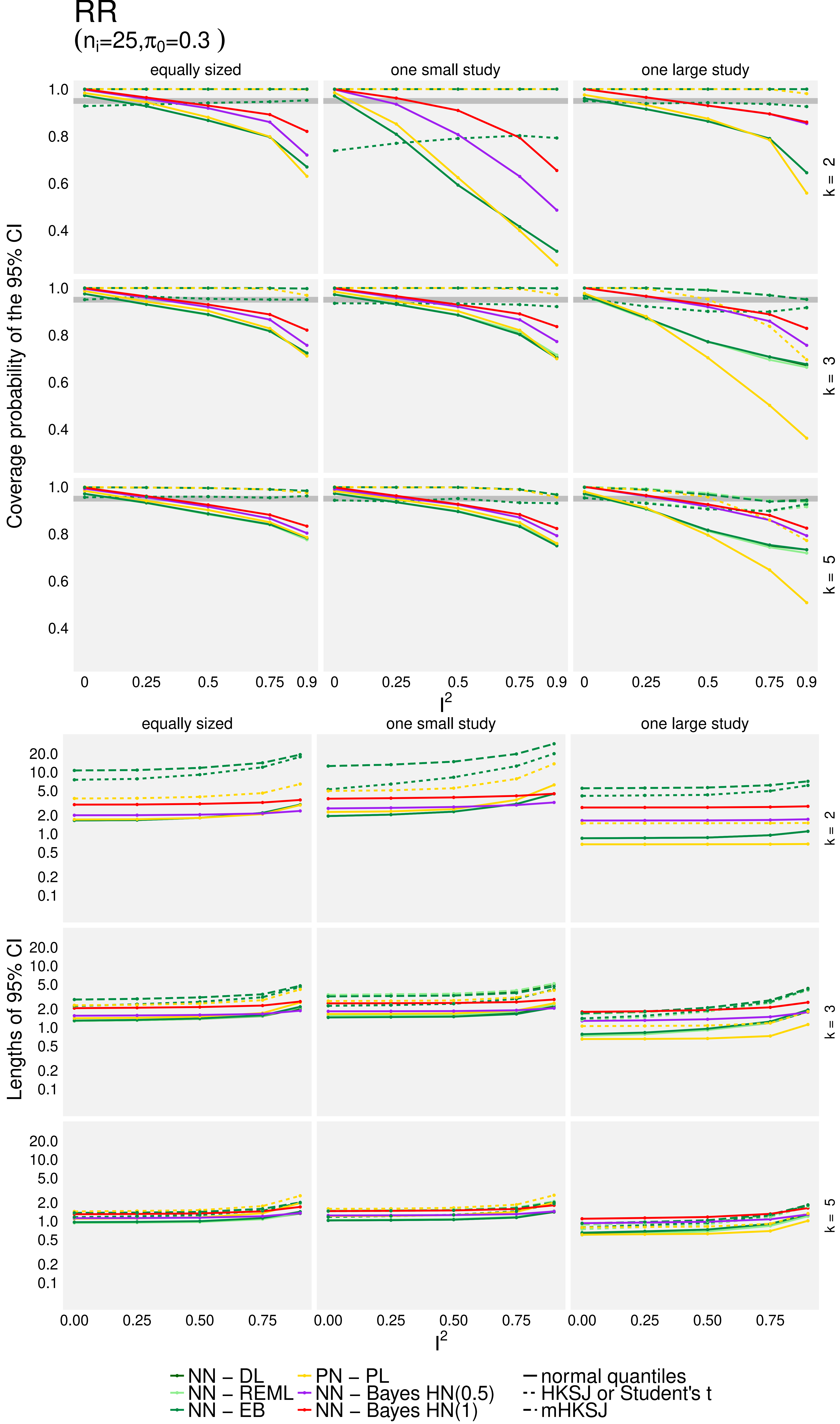
RR
(n_i=25,π_0=0.3)
equally sized
one small study
one large study
Coverage probability of the 95% CI
Lengths of 95% CI
k = 2
k = 3
k = 5
I^2
NN – DL
NN – REML
NN – EB
PN – PL
NN – Bayes HN(0.5)
NN – Bayes HN(1)
normal quantiles
HKSJ or Student's t
mHKSJ

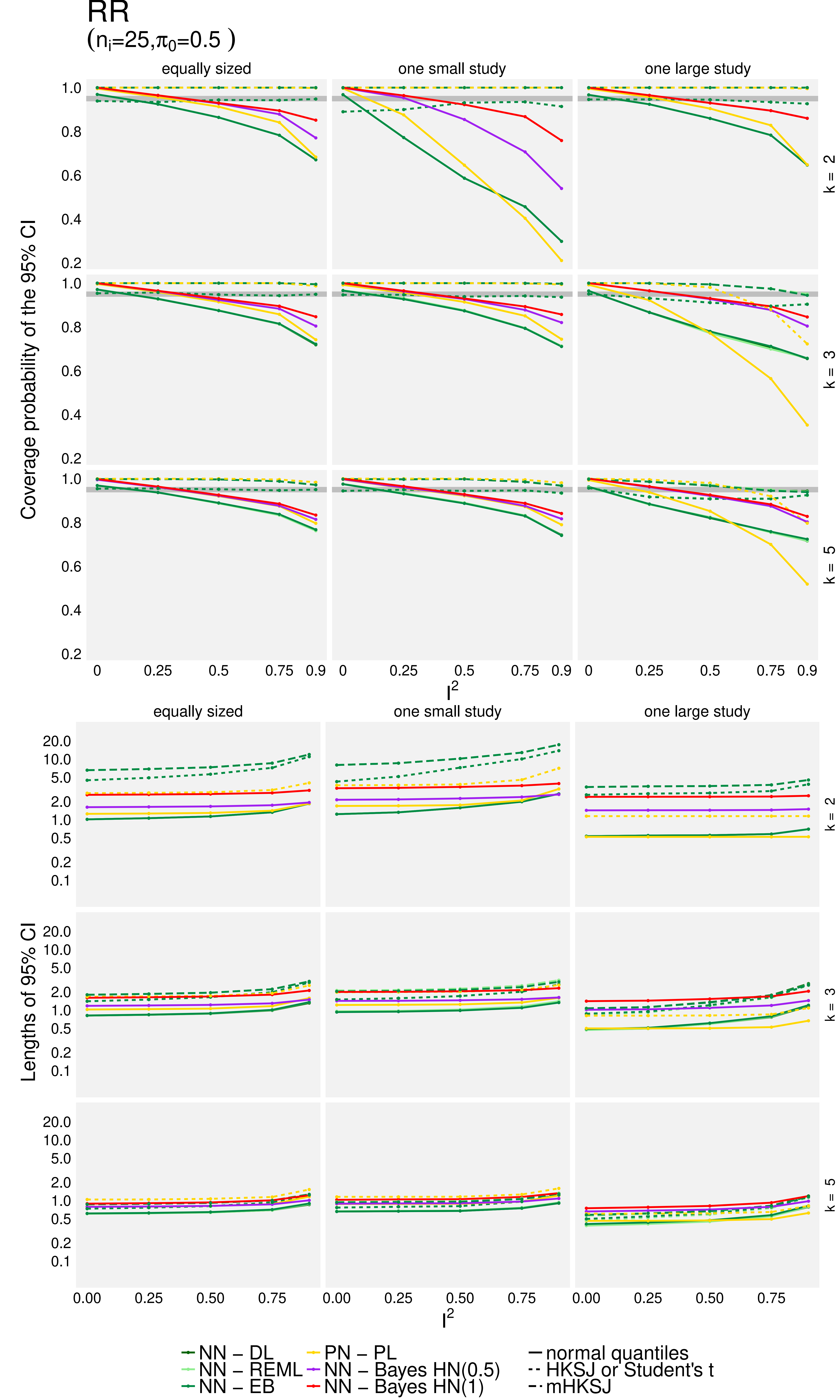

RR
(n_i=25,π_0=0.5)
equally sized
one small study
one large study
Coverage probability of the 95% CI
k = 2
k = 3
k = 5
I^2
Lengths of 95% CI
NN – DL
NN – REML
NN – EB
PN – PL
NN – Bayes HN(0.5)
NN – Bayes HN(1)
normal quantiles
HKSJ or Student's t
mHKSJ

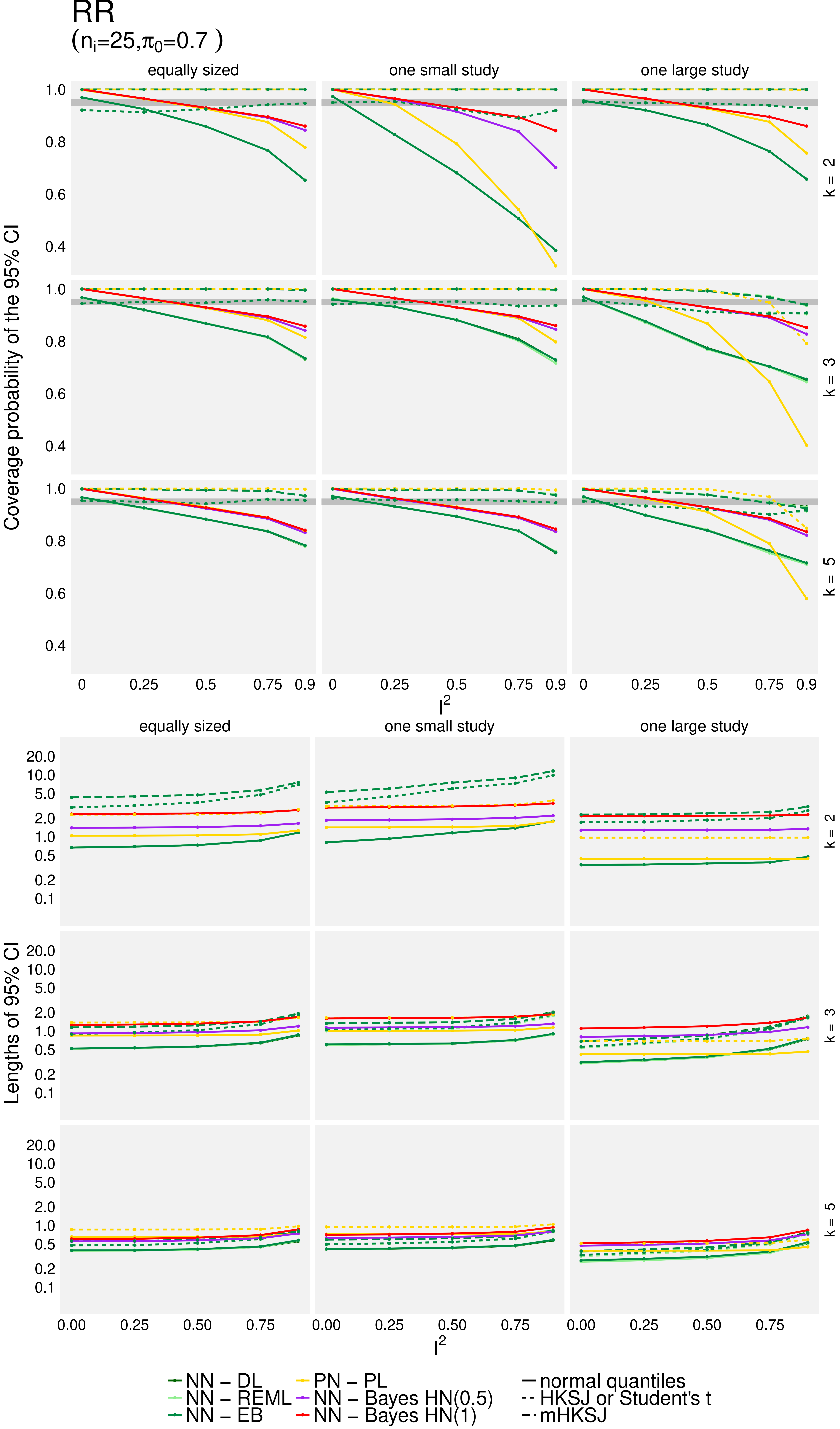

RR
(n_i=25,π_0=0.7)
equally sized
one small study
one large study
Coverage probability of the 95% CI
k = 2
k = 3
k = 5
1.0
0.8
0.6
0.4
0
0.25
0.5
0.75
0.9
I^2
Lengths of 95% CI
20.0
10.0
5.0
2.0
1.0
0.5
0.2
0.1
0.00
0.25
0.50
0.75
NN – DL
NN – REML
NN – EB
PN – PL
NN – Bayes HN(0.5)
NN – Bayes HN(1)
normal quantiles
HKSJ or Student's t
mHKSJ

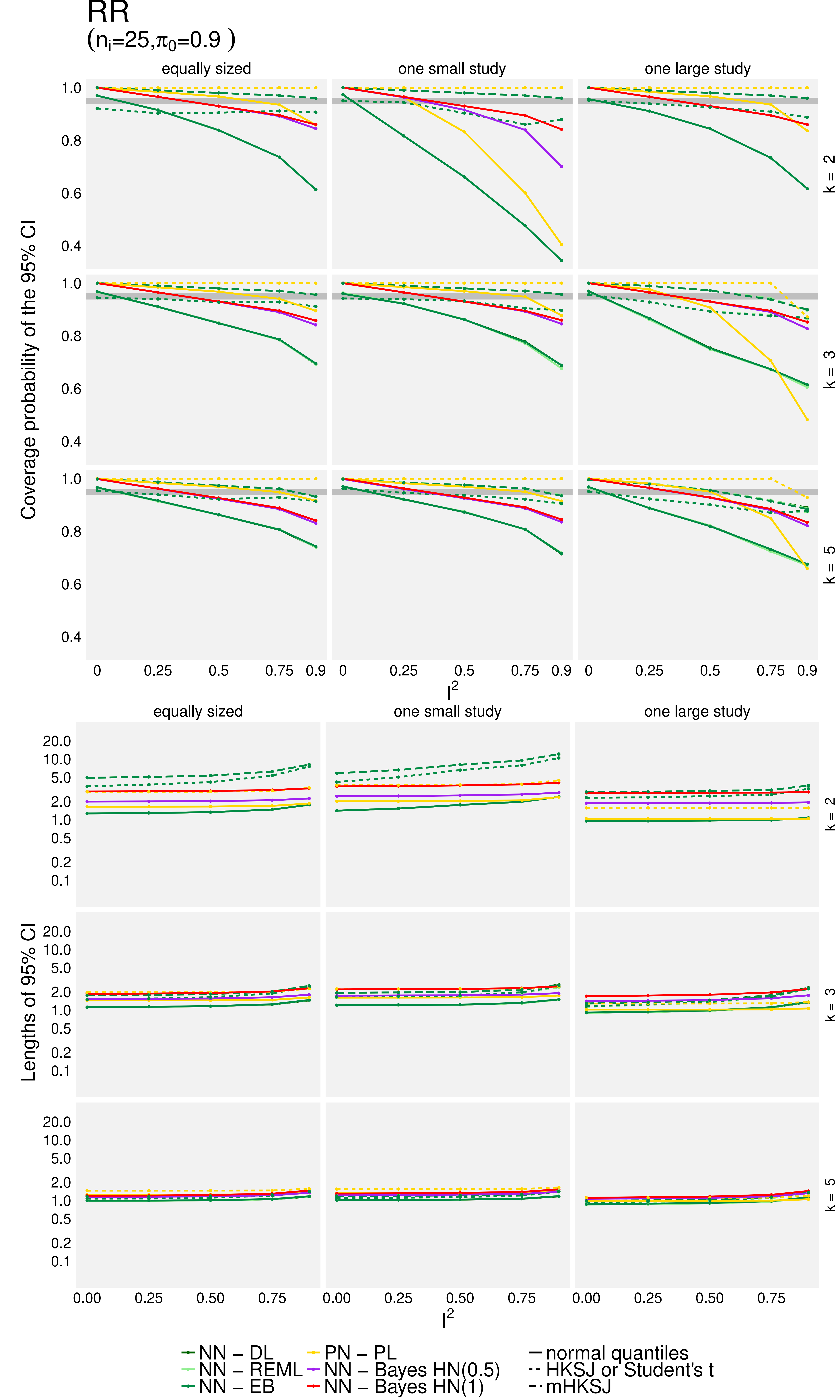

RR
(n_i=25,π_0=0.9)
equally sized
one small study
one large study
Coverage probability of the 95% CI
k = 2
k = 3
k = 5
I^2
Lengths of 95% CI
NN – DL
NN – REML
NN – EB
PN – PL
NN – Bayes HN(0.5)
NN – Bayes HN(1)
normal quantiles
HKSJ or Student's t
mHKSJ

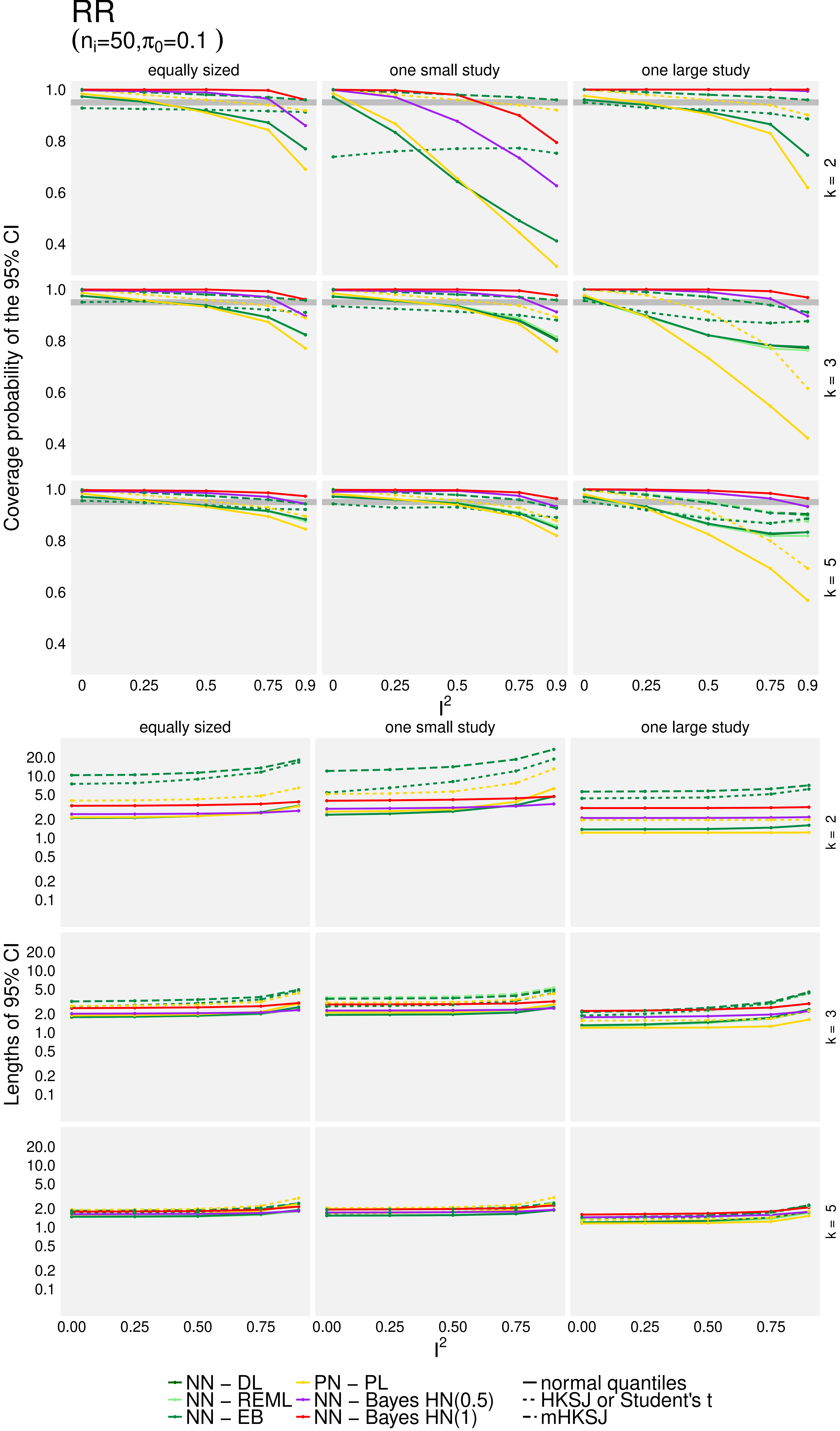
RR
(n_i=50,π_0=0.1)
equally sized
one small study
one large study
Coverage probability of the 95% CI
1.0
0.8
0.6
0.4
k = 2
k = 3
k = 5
0
0.25
0.5
0.75
0.9
I^2
Lengths of 95% CI
20.0
10.0
5.0
2.0
1.0
0.5
0.2
0.1
0.00
0.25
0.50
0.75
NN – DL
NN – REML
NN – EB
PN – PL
NN – Bayes HN(0.5)
NN – Bayes HN(1)
normal quantiles
HKSJ or Student's t
mHKSJ

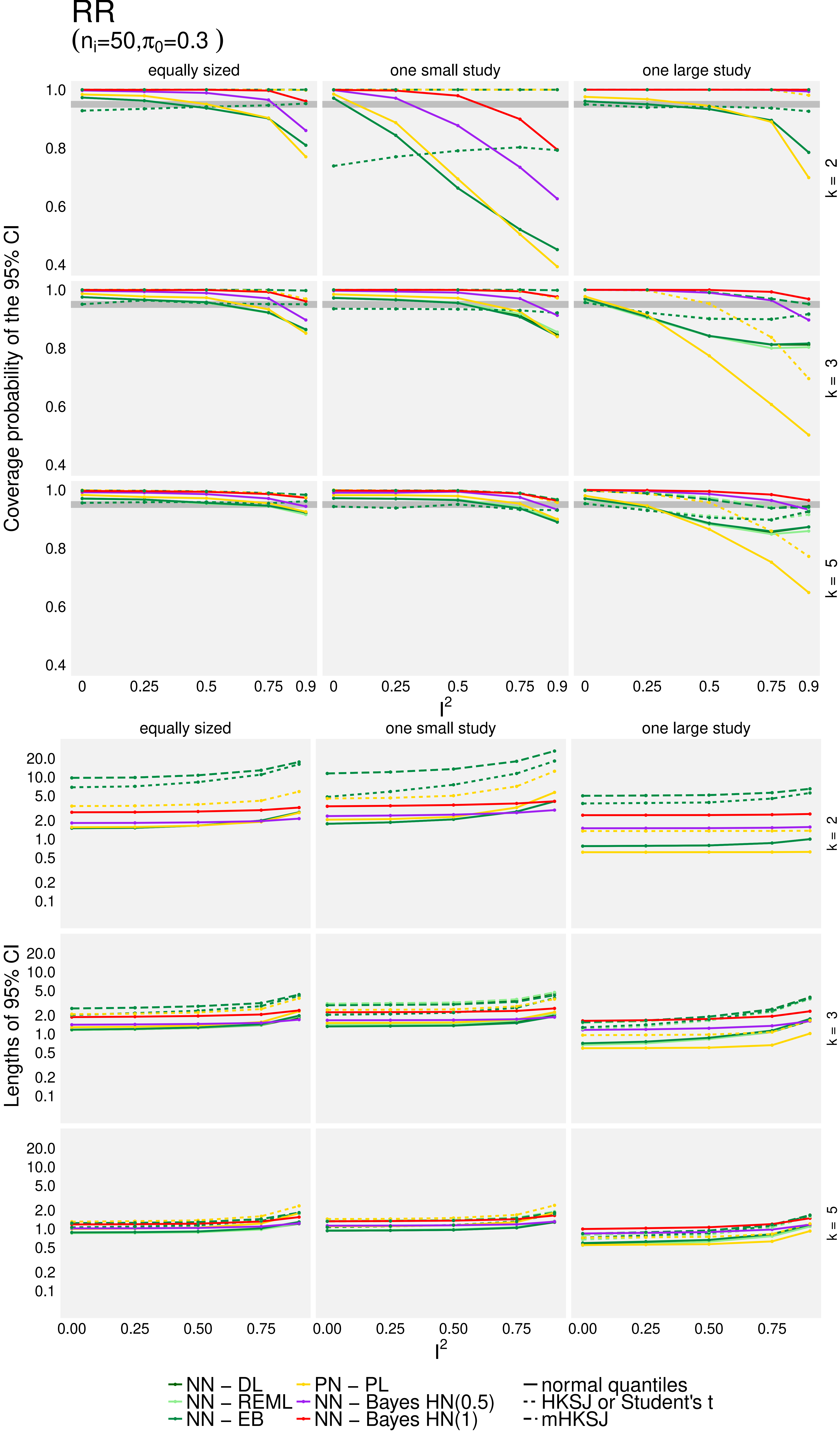

RR
(n_i=50,π_0=0.3)
equally sized
one small study
one large study
Coverage probability of the 95% CI
k = 2
k = 3
k = 5
1.0
0.8
0.6
0.4
0
0.25
0.5
0.75
0.9
I^2
Lengths of 95% CI
20.0
10.0
5.0
2.0
1.0
0.5
0.2
0.1
0.00
0.25
0.50
0.75
NN – DL
NN – REML
NN – EB
PN – PL
NN – Bayes HN(0.5)
NN – Bayes HN(1)
normal quantiles
HKSJ or Student's t
mHKSJ

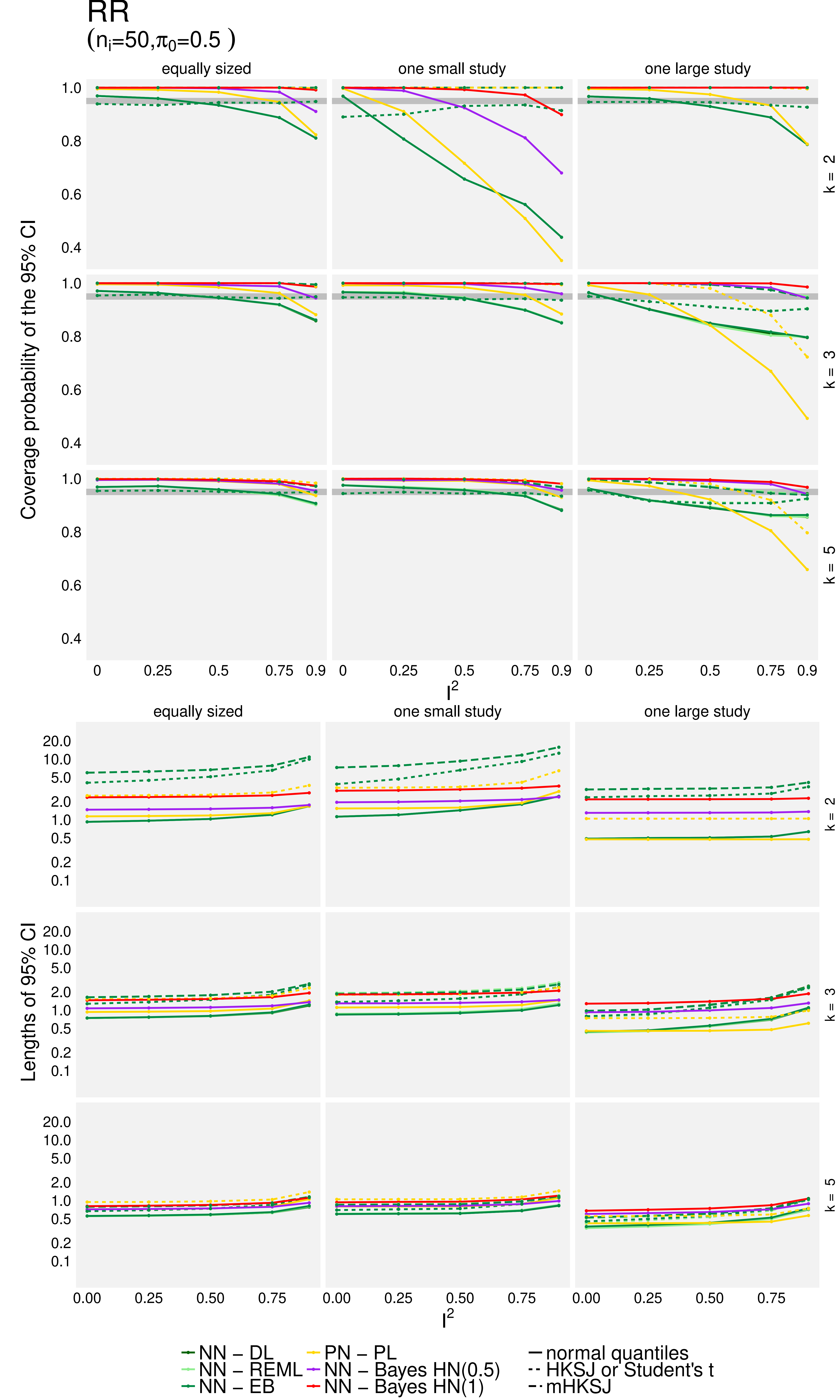

RR
$(n_i=50, \pi_0=0.5)$
equally sized
one small study
one large study
Coverage probability of the 95% CI
k = 2
k = 3
k = 5
I^2
Lengths of 95% CI
NN – DL
NN – REML
NN – EB
PN – PL
NN – Bayes HN(0.5)
NN – Bayes HN(1)
normal quantiles
HKSJ or Student's t
mHKSJ

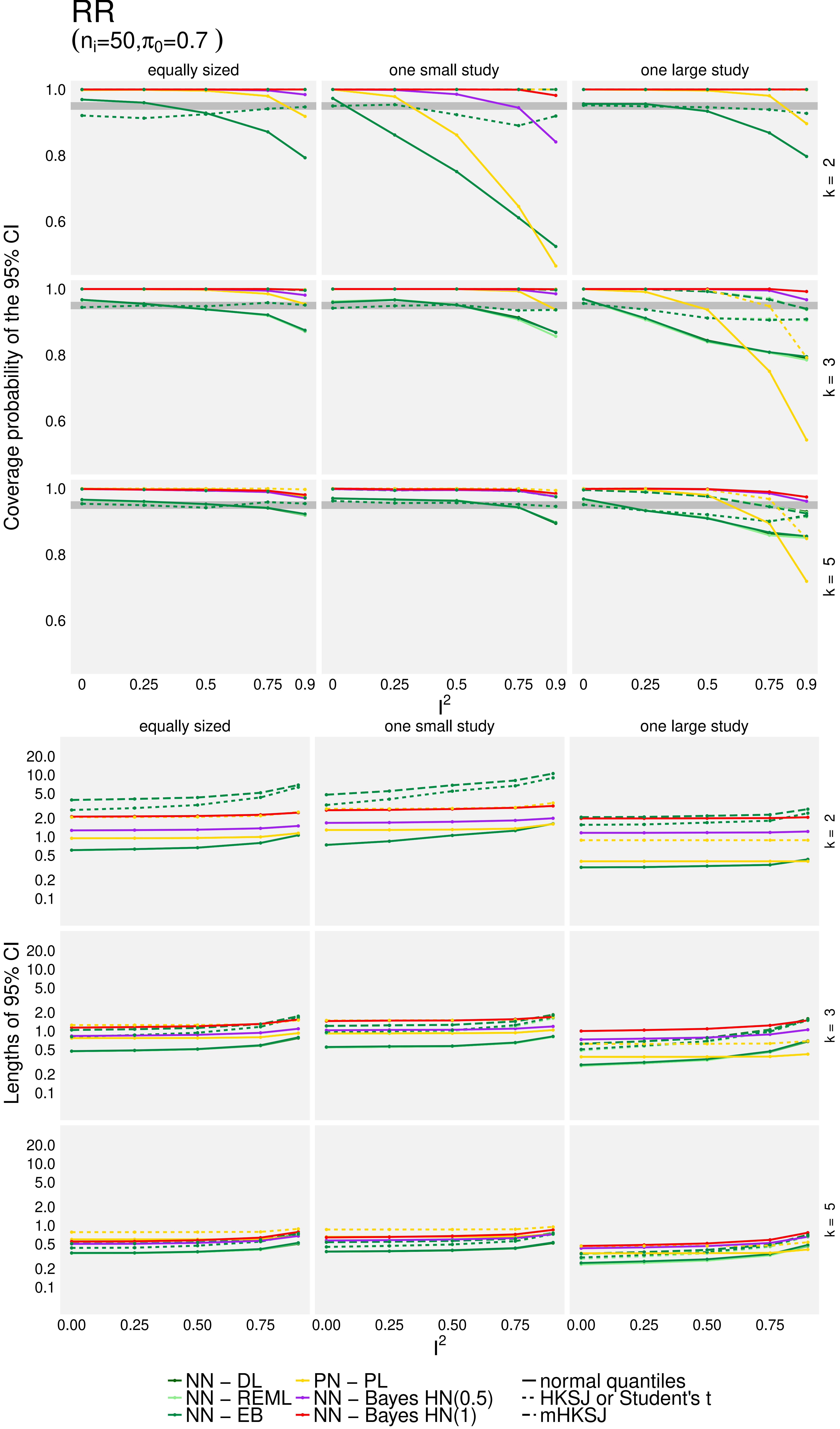
RR
(n_i=50,π_0=0.7)
equally sized
one small study
one large study
Coverage probability of the 95% CI
k = 2
k = 3
k = 5
I^2
Lengths of 95% CI
NN – DL
NN – REML
NN – EB
PN – PL
NN – Bayes HN(0.5)
NN – Bayes HN(1)
normal quantiles
HKSJ or Student's t
mHKSJ

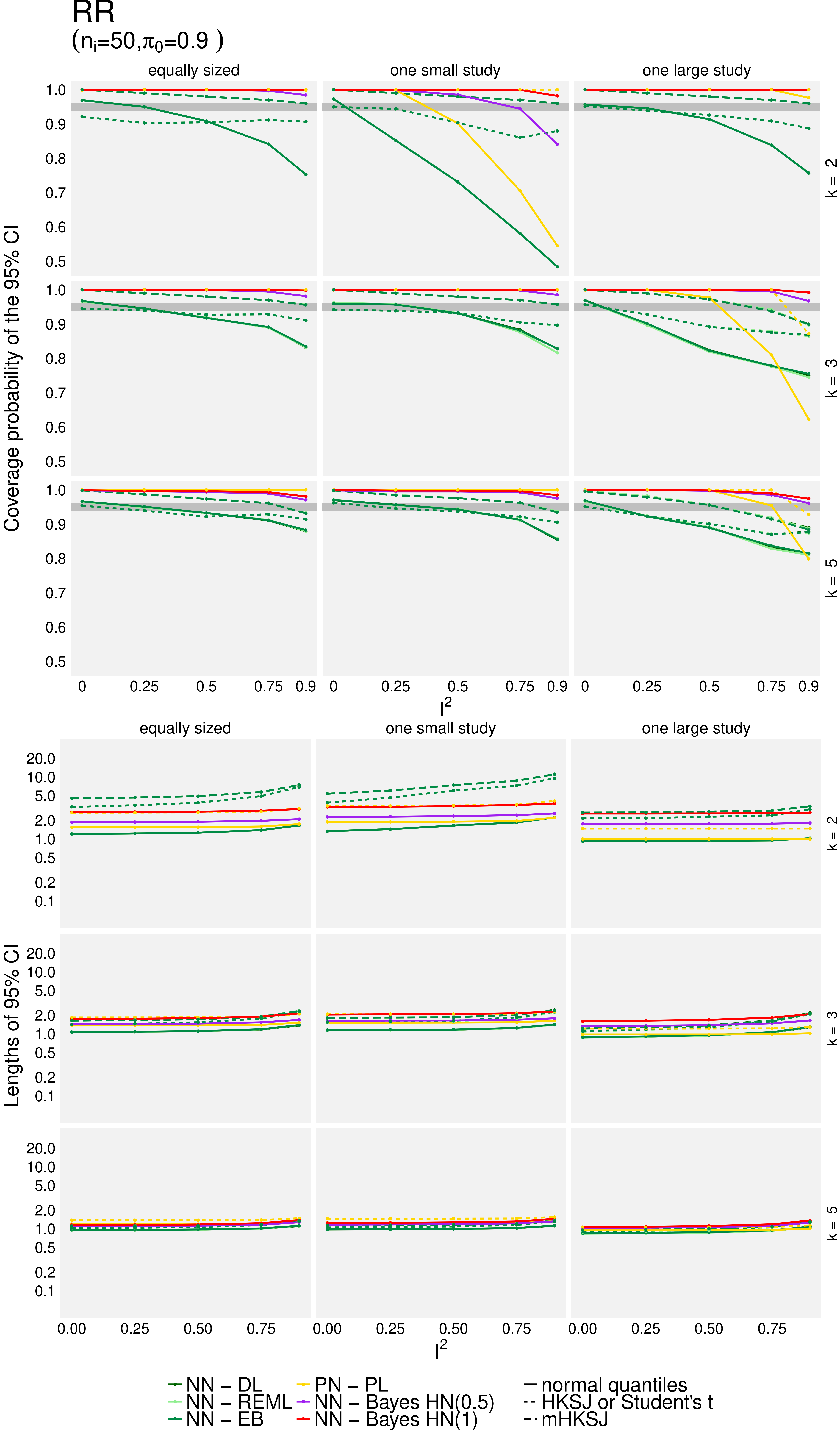
RR
(n_i=50,π_0=0.9)
equally sized
one small study
one large study
Coverage probability of the 95% CI
k = 2
k = 3
k = 5
1.0
0.9
0.8
0.7
0.6
0.5
0
0.25
0.5
0.75
0.9
I2
Lengths of 95% CI
20.0
10.0
5.0
2.0
1.0
0.5
0.2
0.1
0.00
0.25
0.50
0.75
NN – DL
NN – REML
NN – EB
PN – PL
NN – Bayes HN(0.5)
NN – Bayes HN(1)
normal quantiles
HKSJ or Student's t
mHKSJ

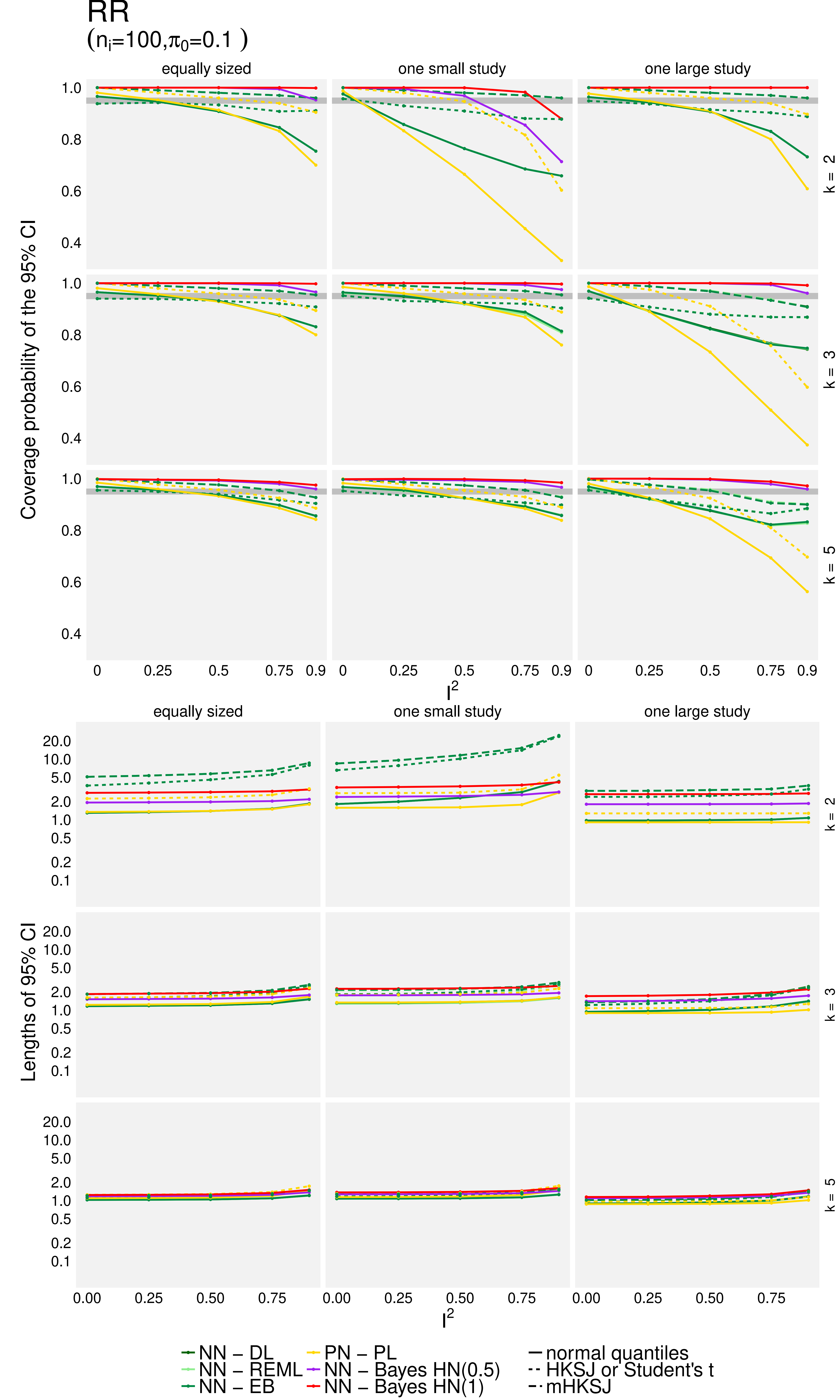

RR
(n_i=100,π_0=0.1)
equally sized
one small study
one large study
Coverage probability of the 95% CI
k = 2
k = 3
k = 5
I^2
Lengths of 95% CI
NN – DL
NN – REML
NN – EB
PN – PL
NN – Bayes HN(0.5)
NN – Bayes HN(1)
normal quantiles
HKSJ or Student's t
mHKSJ

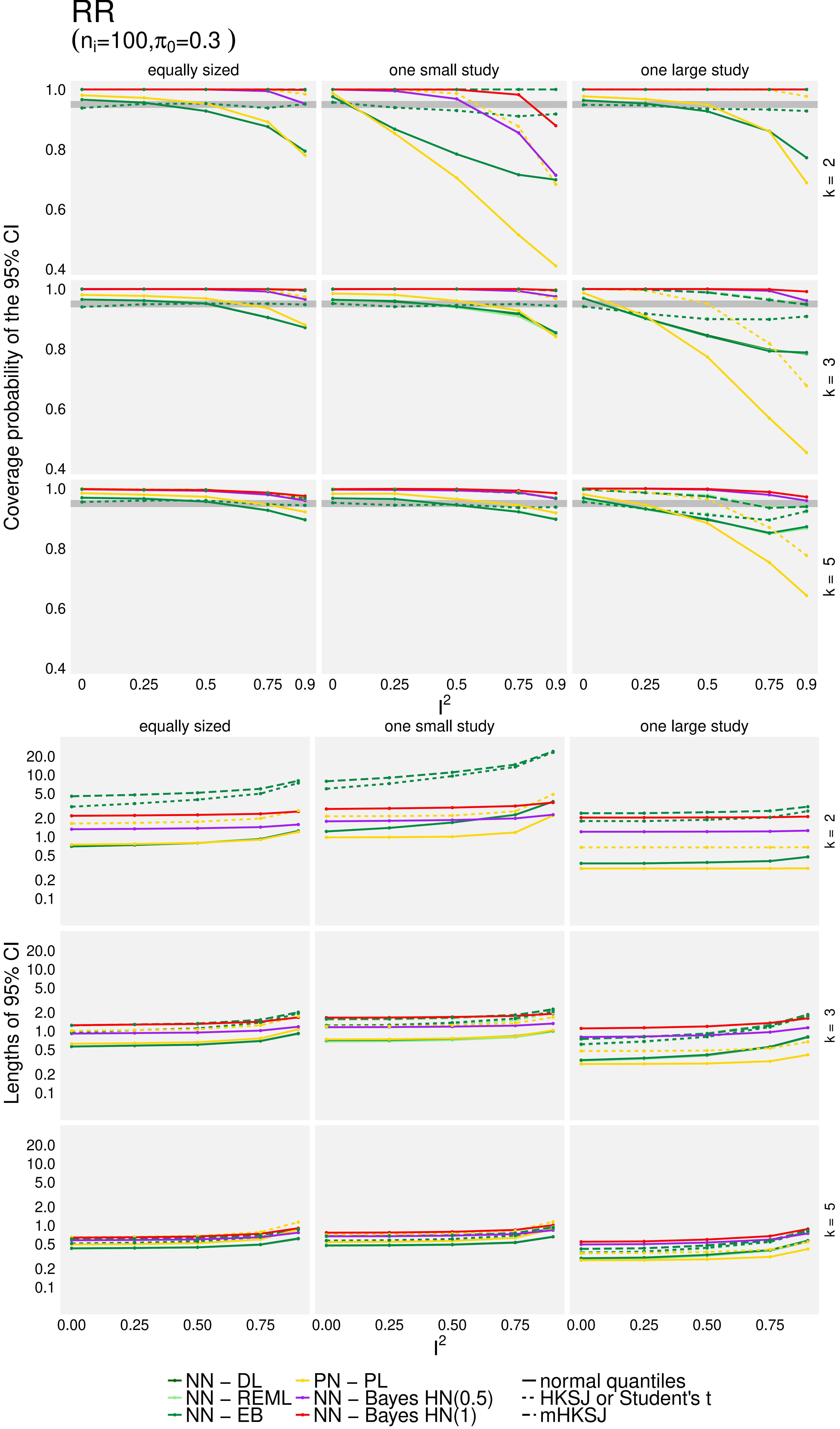
RR
(n_i=100,π_0=0.3)
equally sized
one small study
one large study
Coverage probability of the 95% CI
k = 2
k = 3
k = 5
1.0
0.8
0.6
0.4
0
0.25
0.5
0.75
0.9
I^2
Lengths of 95% CI
20.0
10.0
5.0
2.0
1.0
0.5
0.2
0.1
0.00
0.25
0.50
0.75
NN – DL
NN – REML
NN – EB
PN – PL
NN – Bayes HN(0.5)
NN – Bayes HN(1)
normal quantiles
HKSJ or Student's t
mHKSJ

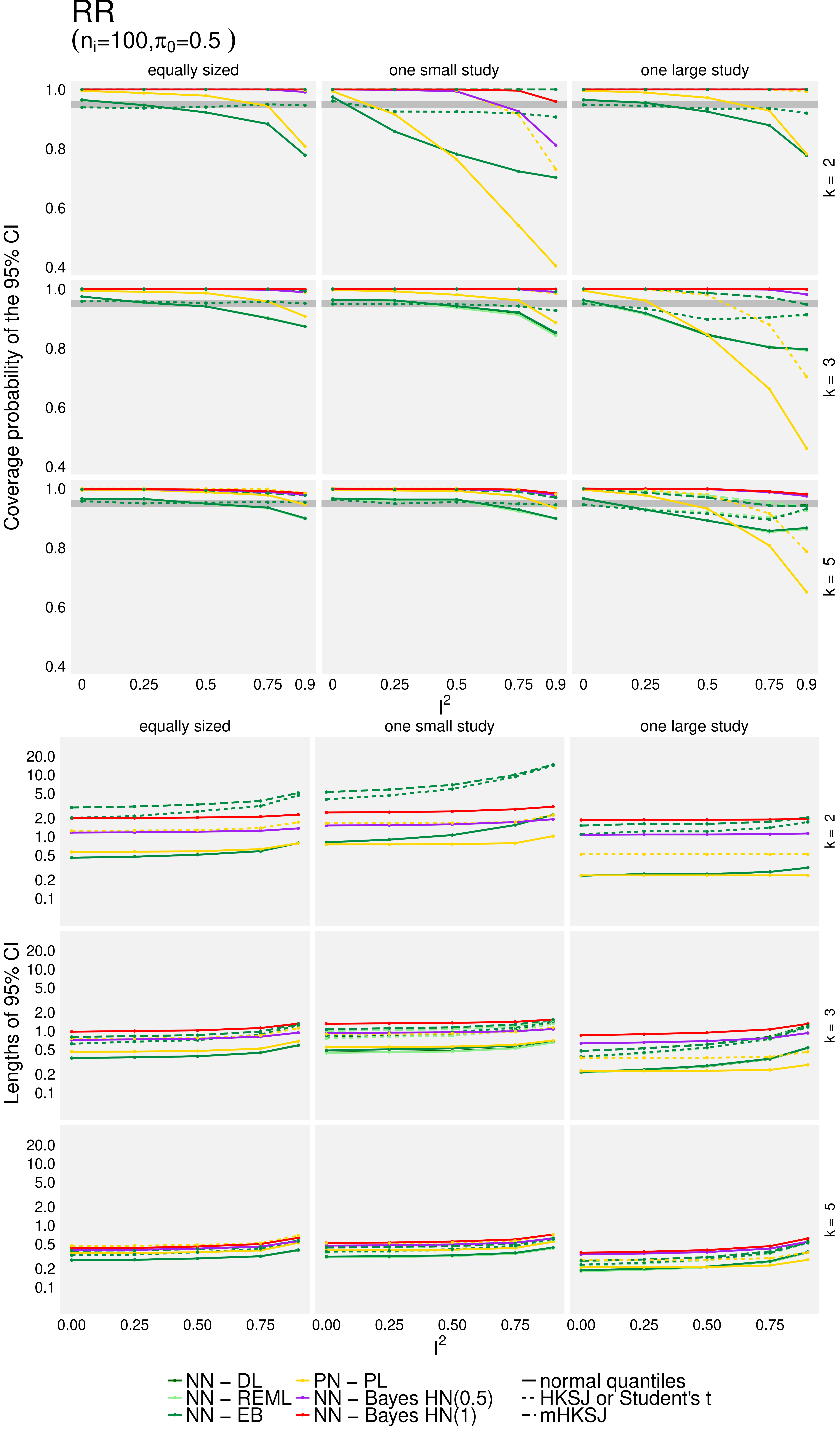

RR
(n_i=100,π_0=0.5)
equally sized
one small study
one large study
Coverage probability of the 95% CI
k = 2
k = 3
k = 5
1.0
0.8
0.6
0.4
0
0.25
0.5
0.75
0.9
I^2
Lengths of 95% CI
20.0
10.0
5.0
2.0
1.0
0.5
0.2
0.1
0.00
0.25
0.50
0.75
NN – DL
NN – REML
NN – EB
PN – PL
NN – Bayes HN(0.5)
NN – Bayes HN(1)
normal quantiles
HKSJ or Student's t
mHKSJ

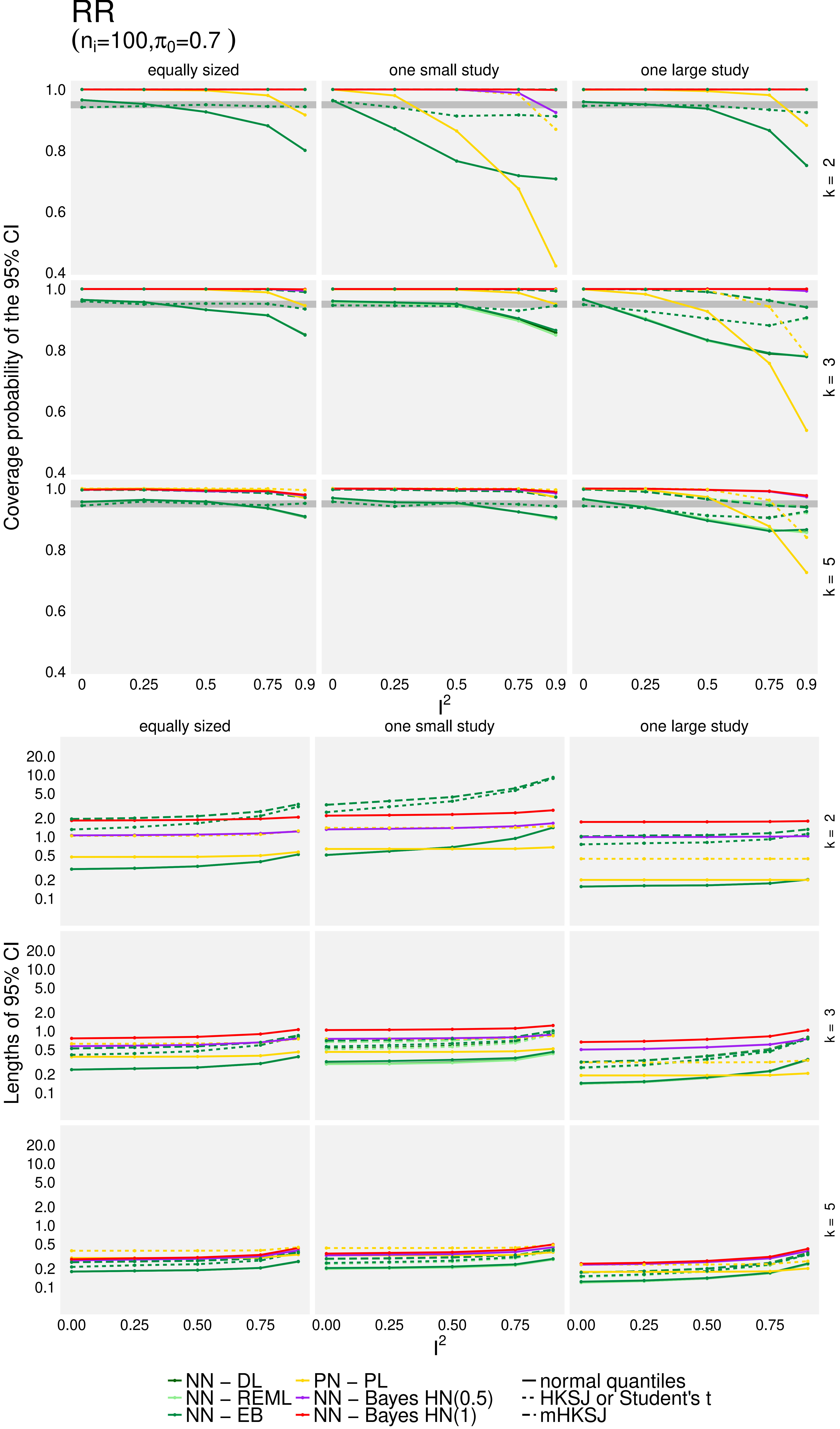

RR
(n_i=100,π_0=0.7)
equally sized
one small study
one large study
Coverage probability of the 95% CI
k = 2
k = 3
k = 5
1.0
0.8
0.6
0.4
0
0.25
0.5
0.75
0.9
I^2
Lengths of 95% CI
20.0
10.0
5.0
2.0
1.0
0.5
0.2
0.1
0.00
0.25
0.50
0.75
NN – DL
NN – REML
NN – EB
PN – PL
NN – Bayes HN(0.5)
NN – Bayes HN(1)
normal quantiles
HKSJ or Student's t
mHKSJ

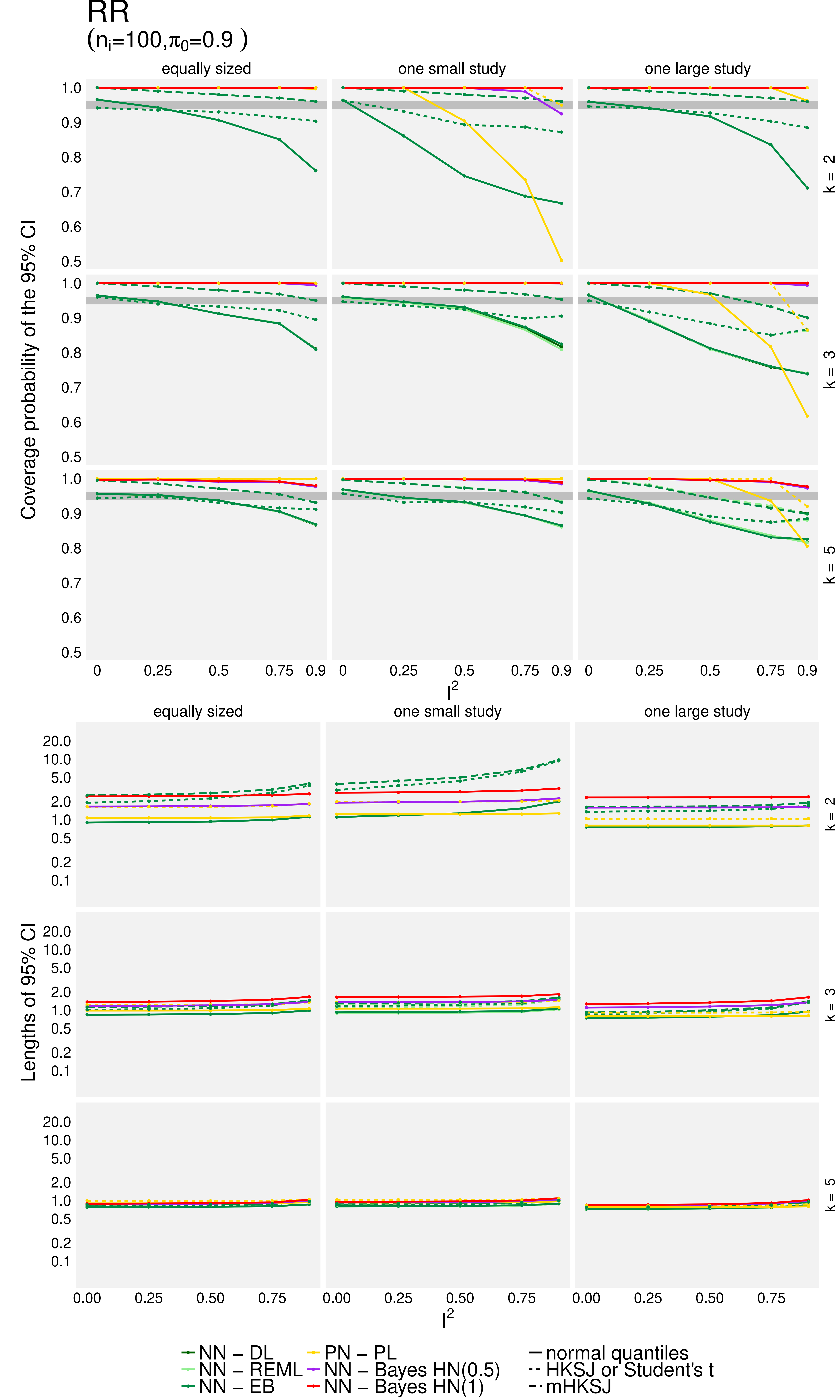

RR
(n_i=100,π_0=0.9)
equally sized
one small study
one large study
Coverage probability of the 95% CI
1.0
0.9
0.8
0.7
0.6
0.5
k = 2
k = 3
k = 5
0
0.25
0.5
0.75
0.9
I²
Lengths of 95% CI
20.0
10.0
5.0
2.0
1.0
0.5
0.2
0.1
0.00
0.25
0.50
0.75
NN – DL
NN – REML
NN – EB
PN – PL
NN – Bayes HN(0.5)
NN – Bayes HN(1)
normal quantiles
HKSJ or Student's t
mHKSJ

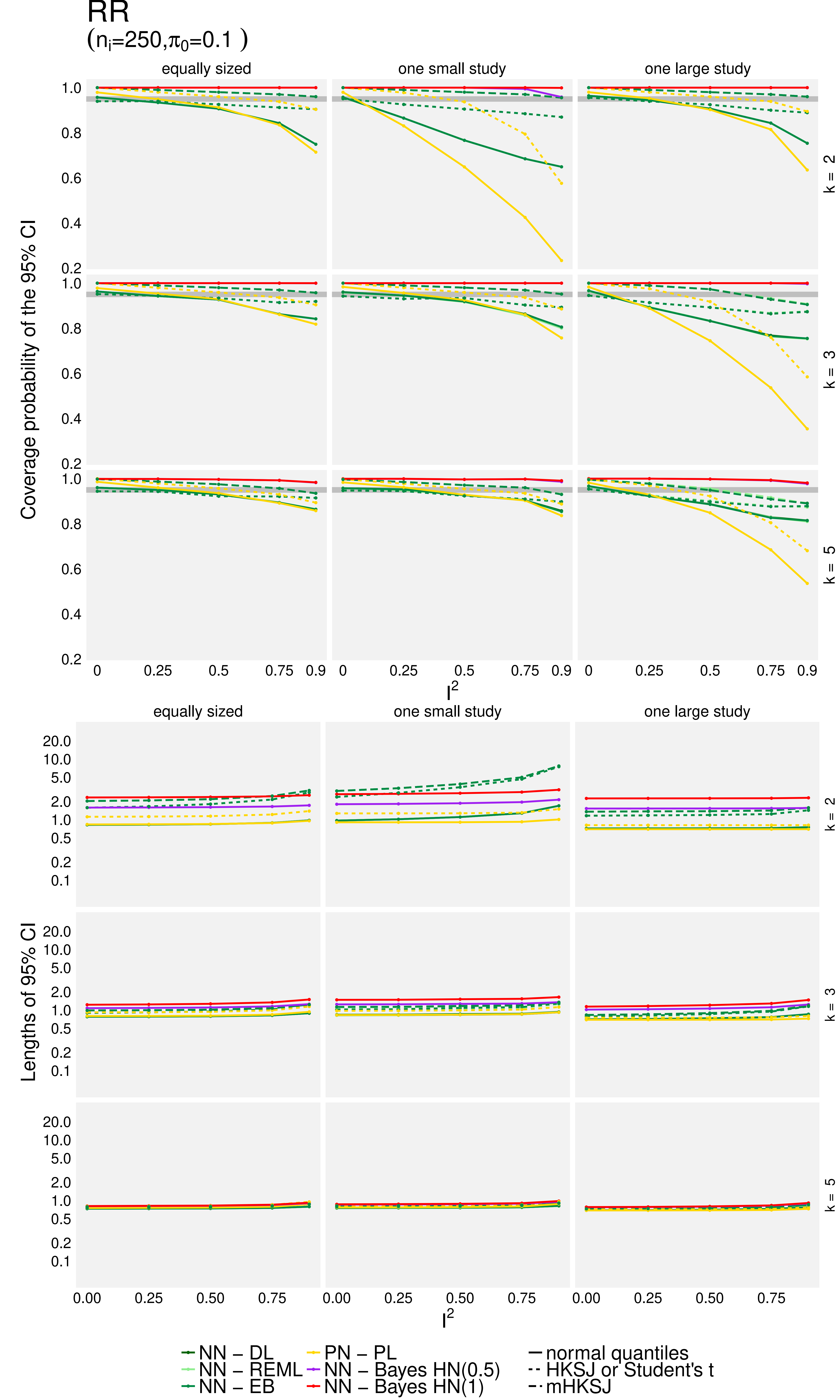
RR
(n_i=250,π_0=0.1)
equally sized
one small study
one large study
Coverage probability of the 95% CI
k = 2
k = 3
k = 5
I²
Lengths of 95% CI
NN – DL
NN – REML
NN – EB
PN – PL
NN – Bayes HN(0.5)
NN – Bayes HN(1)
normal quantiles
HKSJ or Student's t
mHKSJ

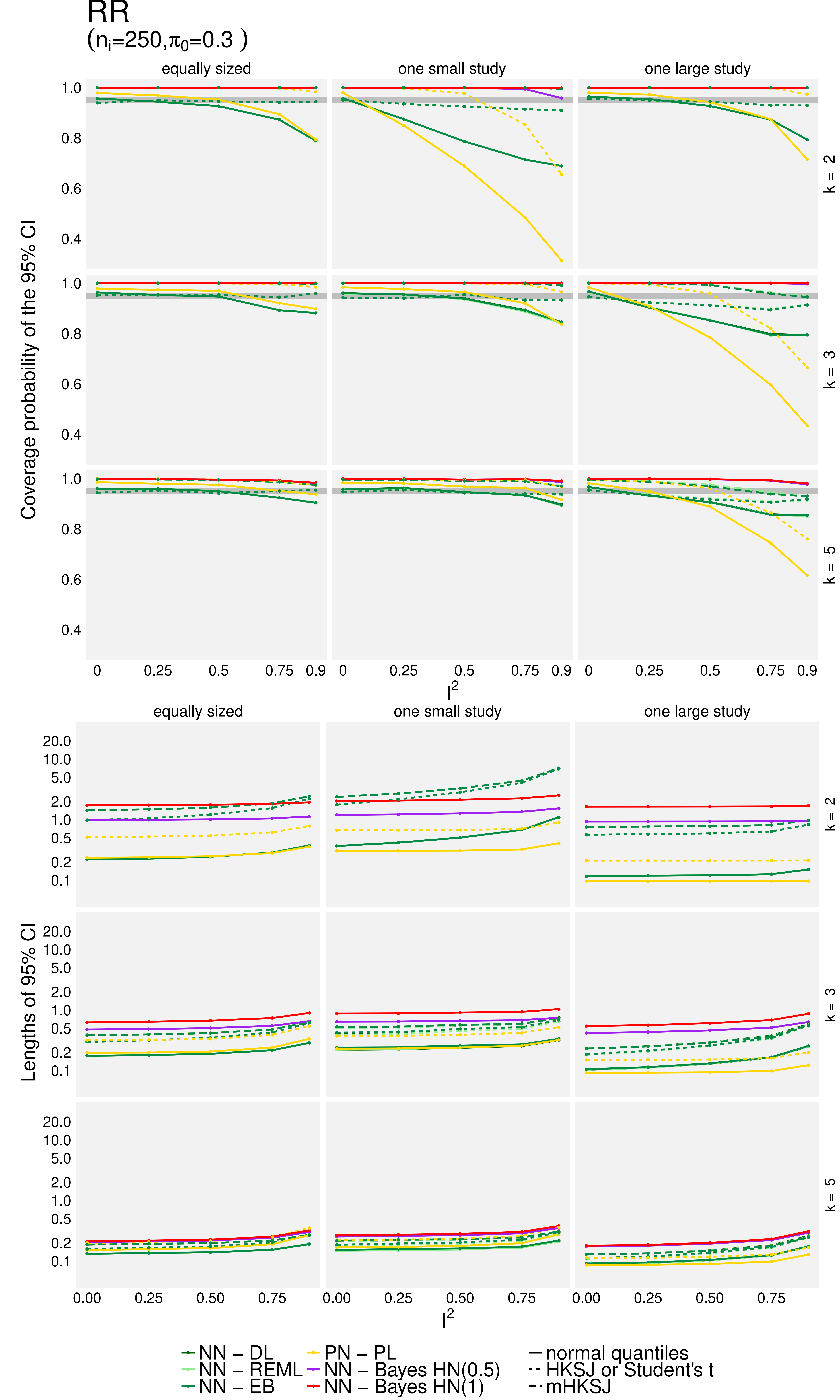

RR
(n_i=250,π_0=0.3)
equally sized
one small study
one large study
Coverage probability of the 95% CI
k = 2
k = 3
k = 5
I^2
Lengths of 95% CI
NN – DL
NN – REML
NN – EB
PN – PL
NN – Bayes HN(0.5)
NN – Bayes HN(1)
normal quantiles
HKSJ or Student's t
mHKSJ

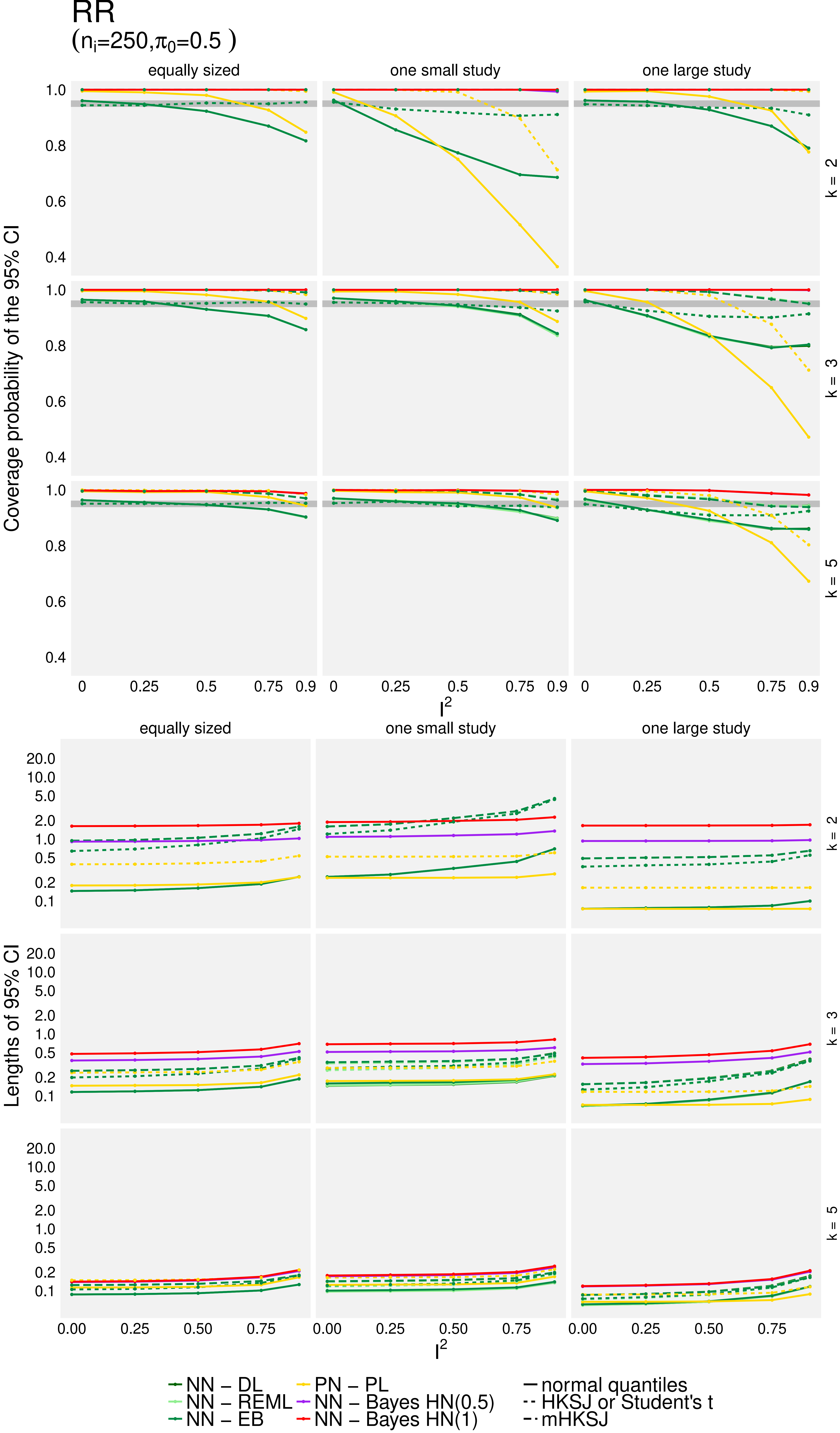
RR
(n_i=250,π_0=0.5)
equally sized
one small study
one large study
Coverage probability of the 95% CI
k = 2
k = 3
k = 5
1.0
0.8
0.6
0.4
0
0.25
0.5
0.75
0.9
I^2
Lengths of 95% CI
20.0
10.0
5.0
2.0
1.0
0.5
0.2
0.1
0.00
0.25
0.50
0.75
NN – DL
NN – REML
NN – EB
PN – PL
NN – Bayes HN(0.5)
NN – Bayes HN(1)
normal quantiles
HKSJ or Student's t
mHKSJ

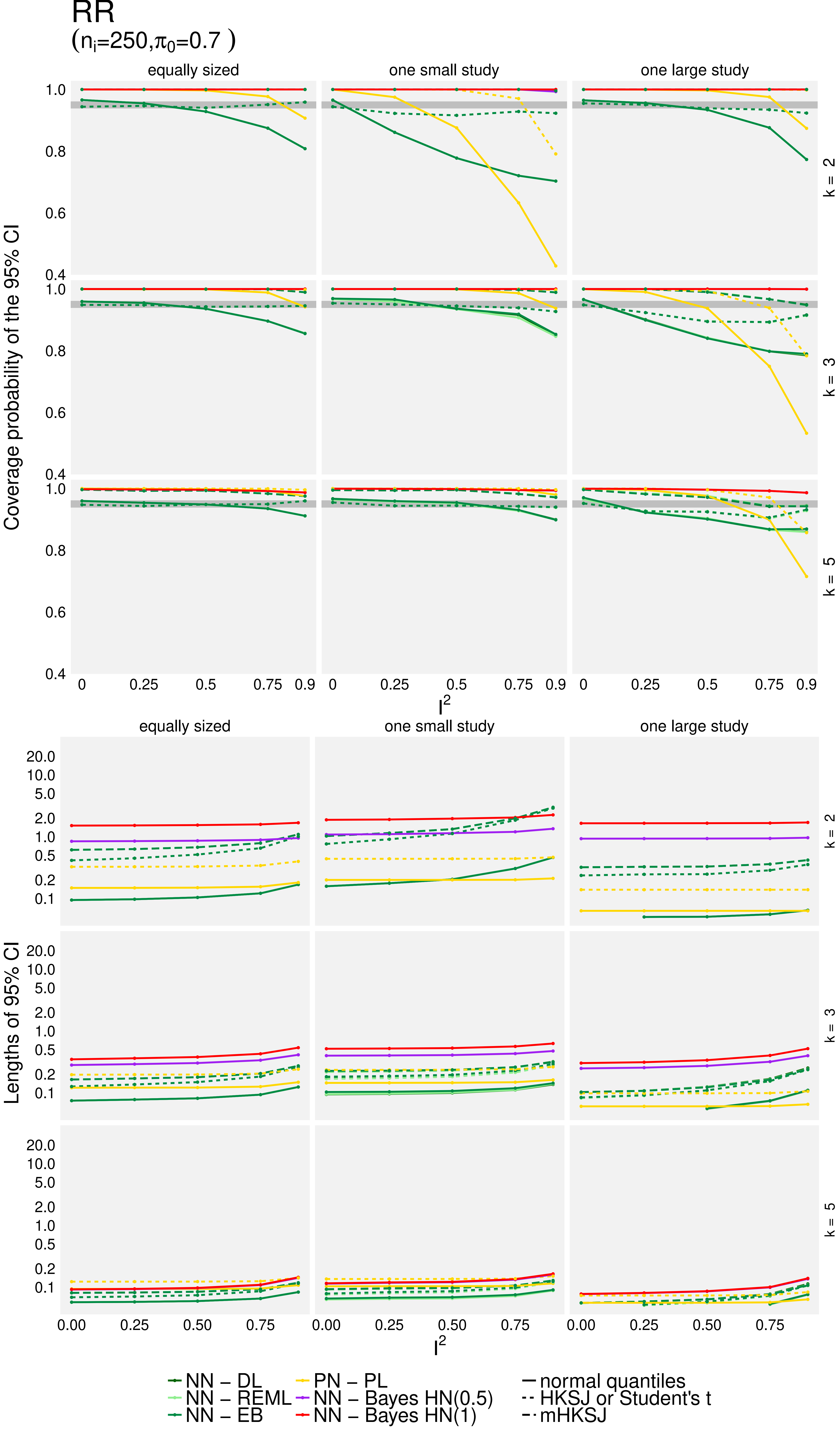

RR
(n_i=250,π_0=0.7)
equally sized
one small study
one large study
Coverage probability of the 95% CI
k = 2
k = 3
k = 5
I^2
Lengths of 95% CI
NN – DL
NN – REML
NN – EB
PN – PL
NN – Bayes HN(0.5)
NN – Bayes HN(1)
normal quantiles
HKSJ or Student's t
mHKSJ

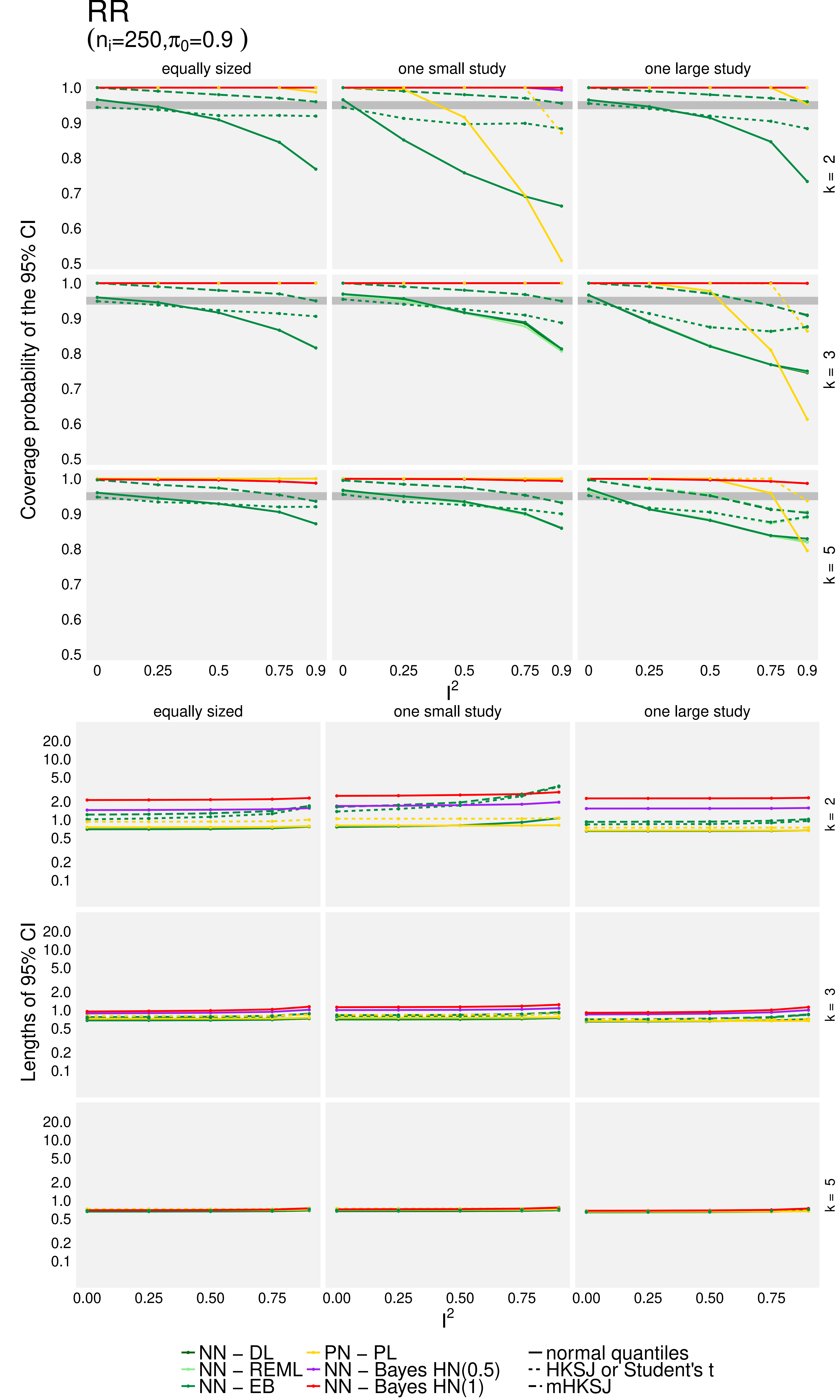

RR
(n_i=250,π_0=0.9)
equally sized
one small study
one large study
Coverage probability of the 95% CI
Lengths of 95% CI
k = 2
k = 3
k = 5
I^2
NN – DL
NN – REML
NN – EB
PN – PL
NN – Bayes HN(0.5)
NN – Bayes HN(1)
normal quantiles
HKSJ or Student's t
mHKSJ

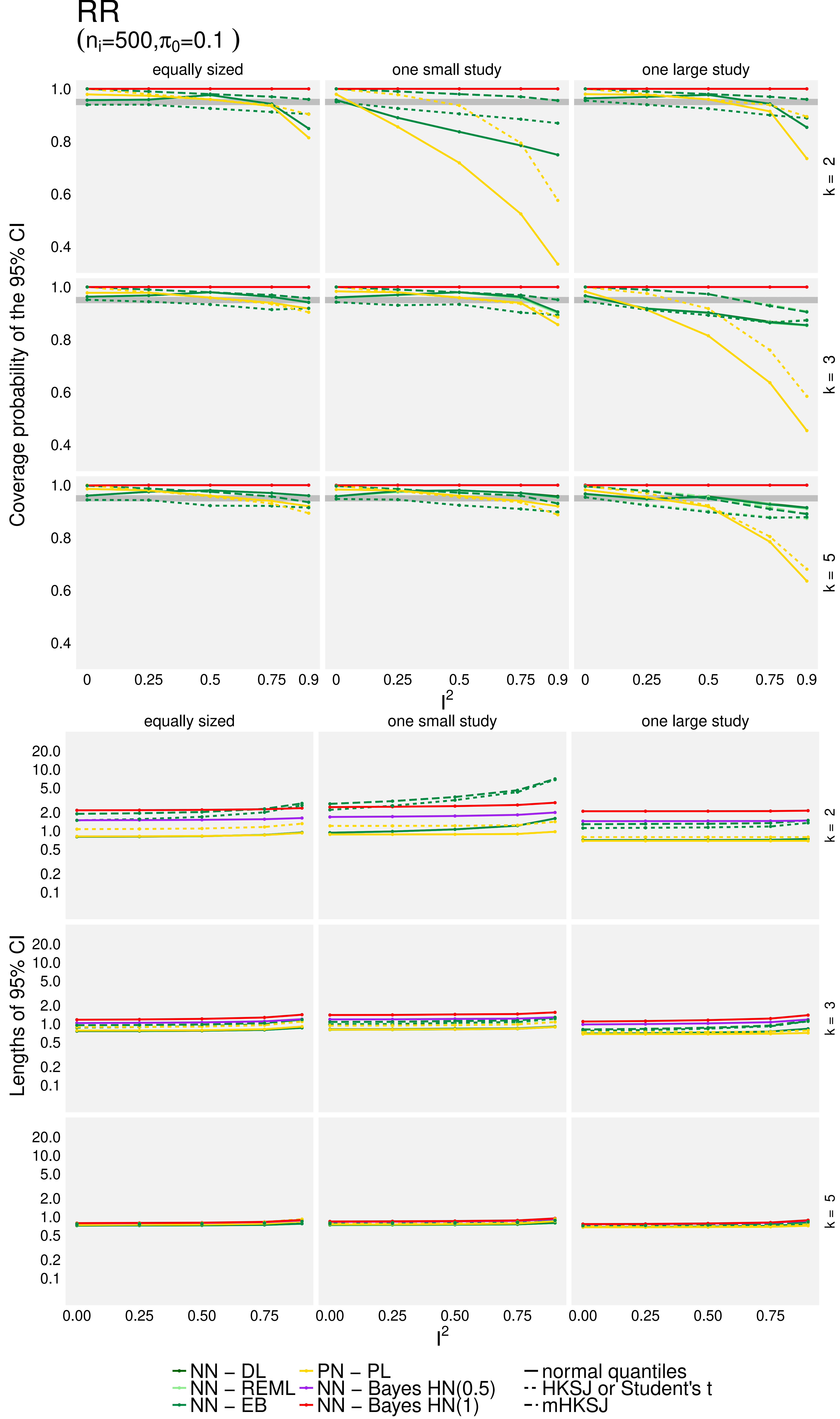

RR
(n_i=500,π_0=0.1)
equally sized
one small study
one large study
Coverage probability of the 95% CI
k = 2
k = 3
k = 5
I^2
Lengths of 95% CI
NN – DL
NN – REML
NN – EB
PN – PL
NN – Bayes HN(0.5)
NN – Bayes HN(1)
normal quantiles
HKSJ or Student's t
mHKSJ

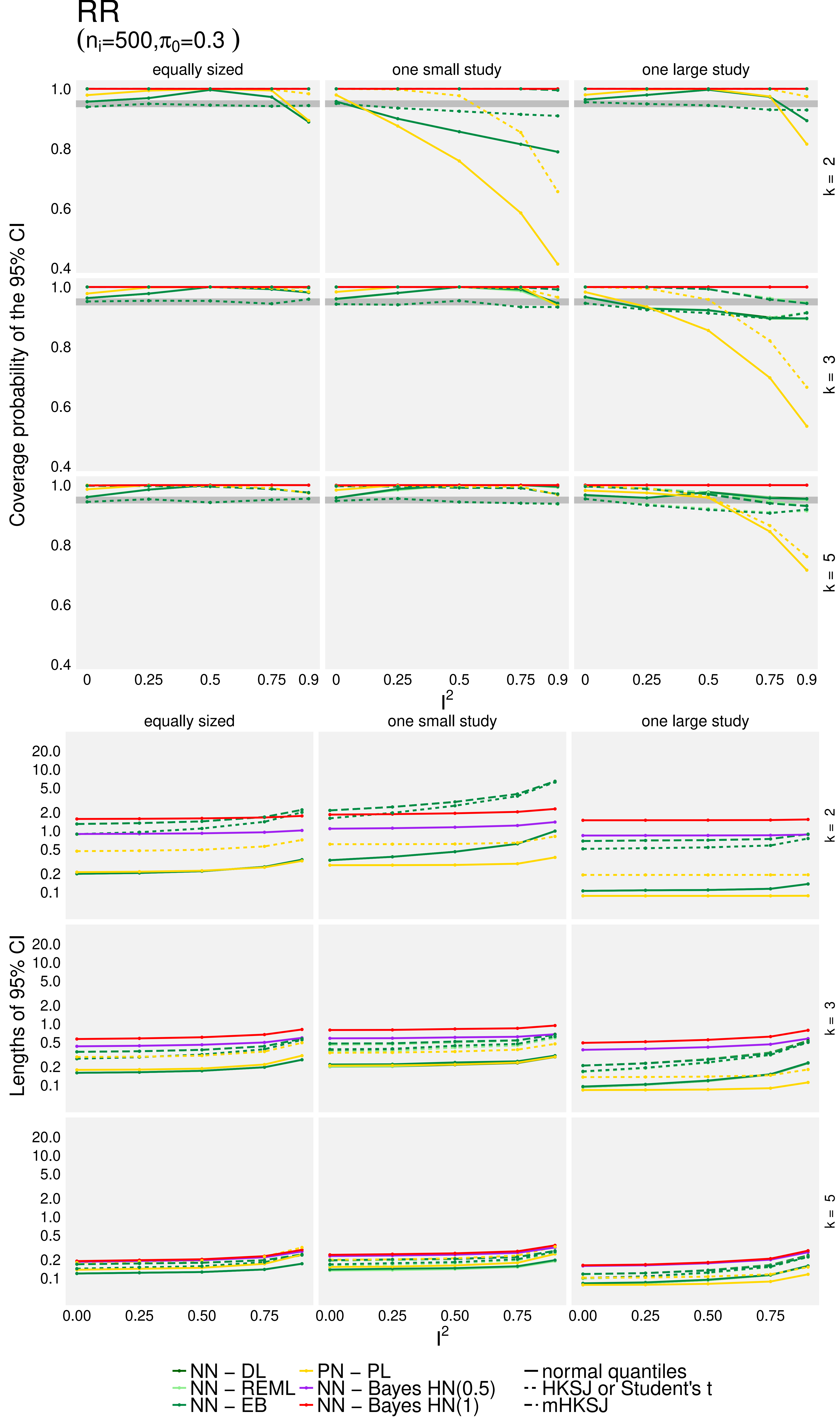

RR
(n_i=500,π_0=0.3)
equally sized
one small study
one large study
Coverage probability of the 95% CI
1.0
0.8
0.6
0.4
k = 2
k = 3
k = 5
0
0.25
0.5
0.75
0.9
I^2
Lengths of 95% CI
20.0
10.0
5.0
2.0
1.0
0.5
0.2
0.1
0.00
0.25
0.50
0.75
NN – DL
NN – REML
NN – EB
PN – PL
NN – Bayes HN(0.5)
NN – Bayes HN(1)
normal quantiles
HKSJ or Student's t
mHKSJ

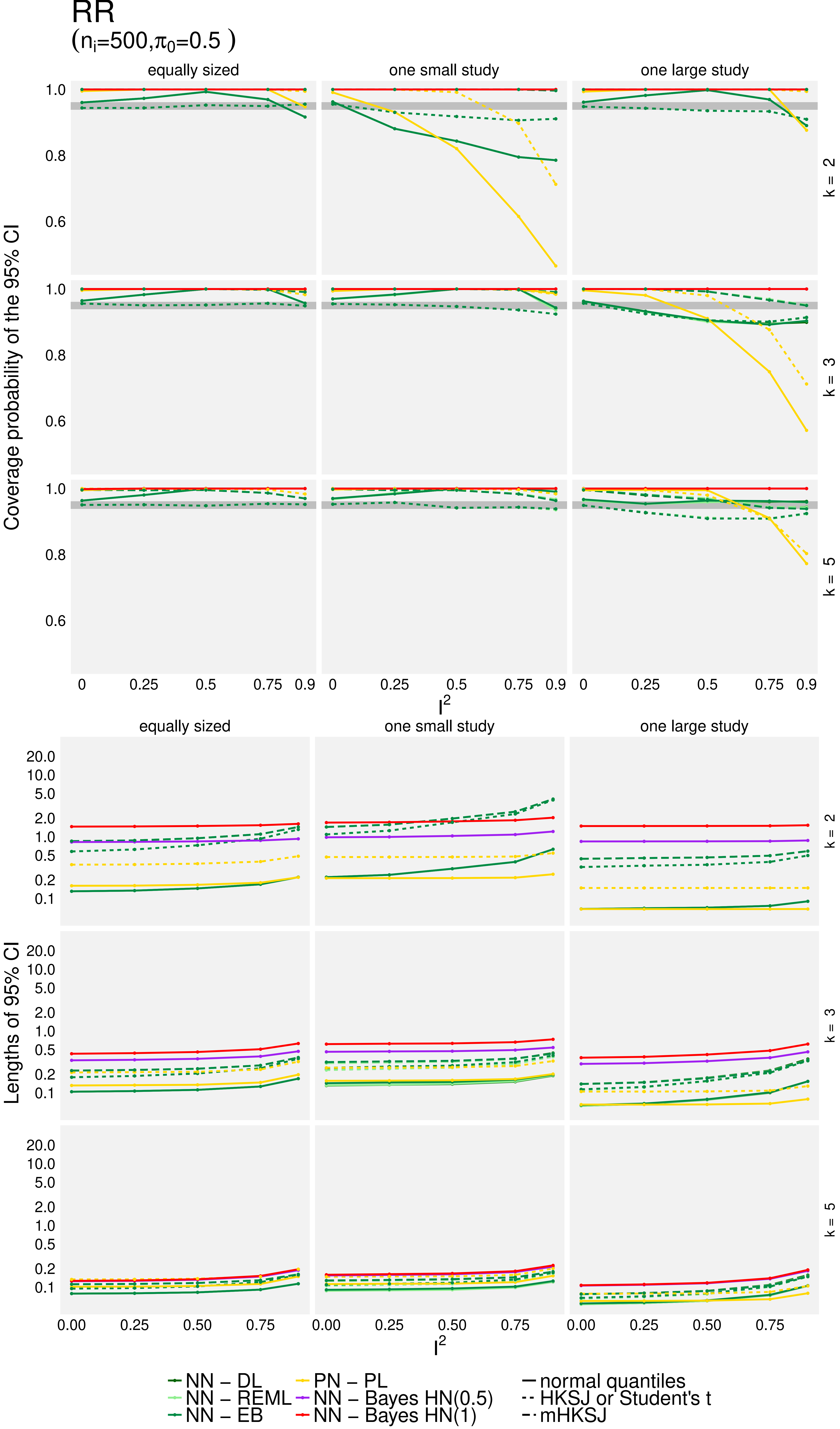

RR
(n_i=500,π_0=0.5)
equally sized
one small study
one large study
Coverage probability of the 95% CI
1.0
0.8
0.6
k = 2
k = 3
k = 5
0
0.25
0.5
0.75
0.9
I^2
Lengths of 95% CI
20.0
10.0
5.0
2.0
1.0
0.5
0.2
0.1
0.00
0.25
0.50
0.75
NN – DL
NN – REML
NN – EB
PN – PL
NN – Bayes HN(0.5)
NN – Bayes HN(1)
normal quantiles
HKSJ or Student's t
mHKSJ

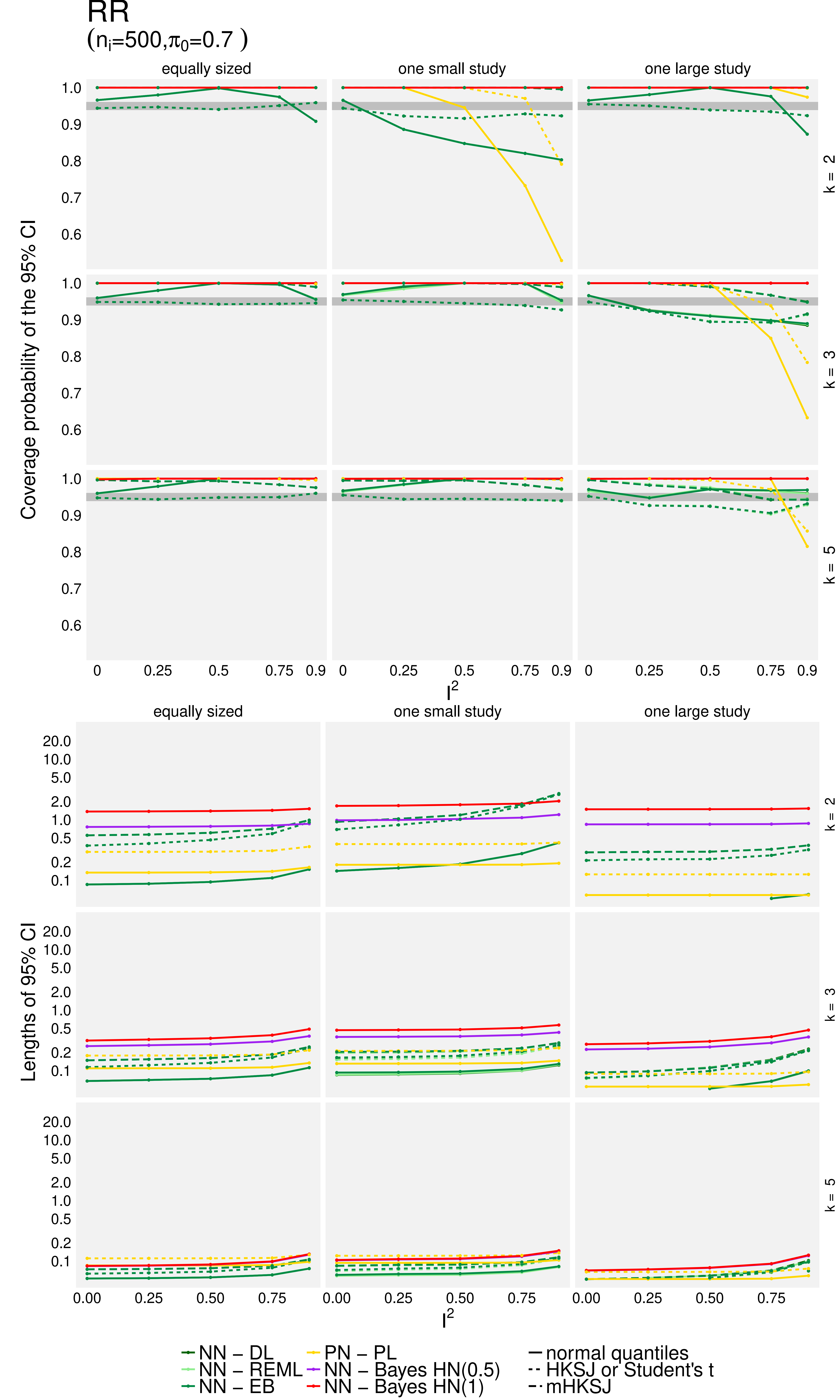
RR
(n_i=500,π_0=0.7)
equally sized
one small study
one large study
Coverage probability of the 95% CI
k = 2
k = 3
k = 5
I^2
Lengths of 95% CI
NN – DL
NN – REML
NN – EB
PN – PL
NN – Bayes HN(0.5)
NN – Bayes HN(1)
normal quantiles
HKSJ or Student's t
mHKSJ

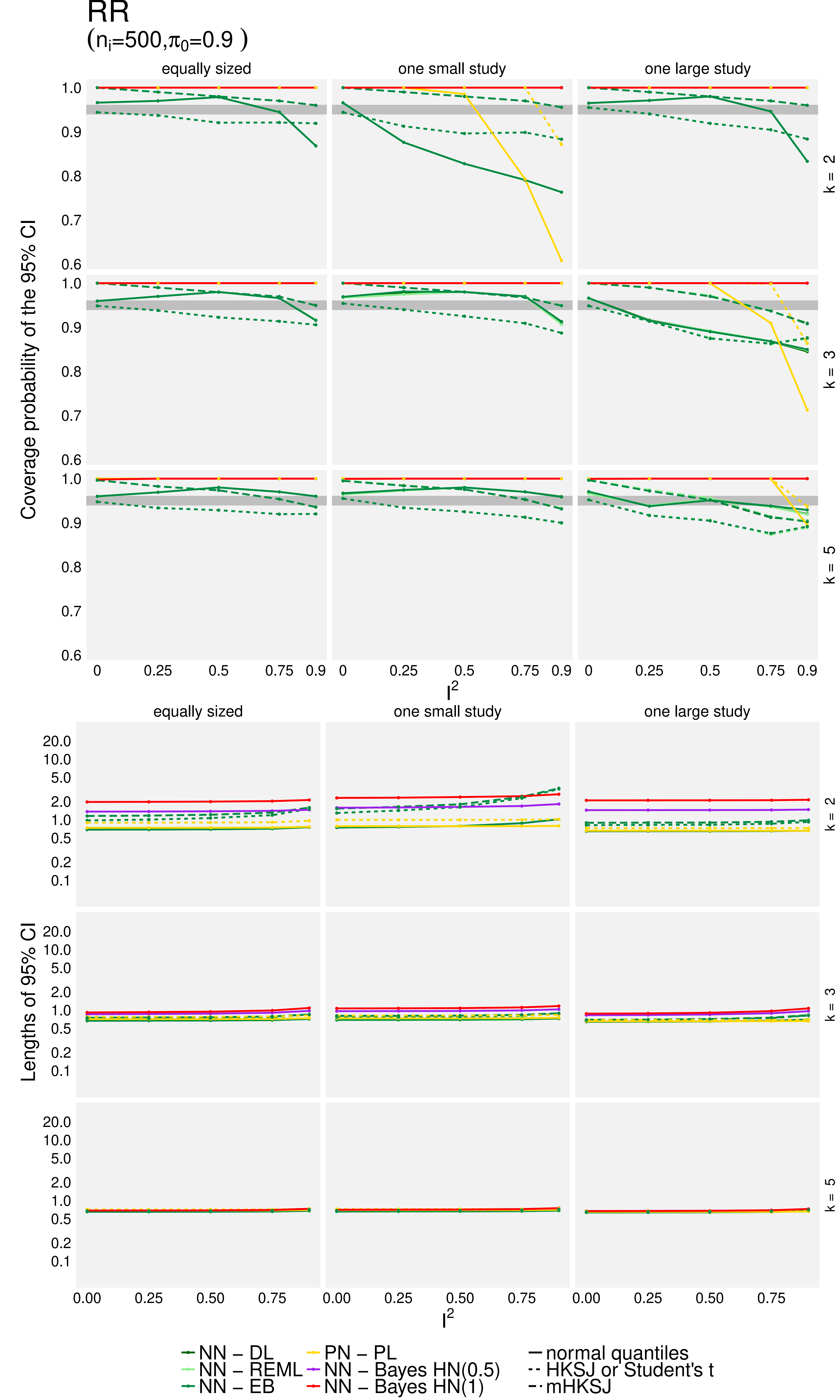

RR
(n_i=500,π_0=0.9)
equally sized
one small study
one large study
Coverage probability of the 95% CI
1.0
0.9
0.8
0.7
0.6
k = 2
k = 3
k = 5
0
0.25
0.5
0.75
0.9
I^2
Lengths of 95% CI
20.0
10.0
5.0
2.0
1.0
0.5
0.2
0.1
0.00
0.25
0.50
0.75
NN – DL
NN – REML
NN – EB
PN – PL
NN – Bayes HN(0.5)
NN – Bayes HN(1)
normal quantiles
HKSJ or Student's t
mHKSJ

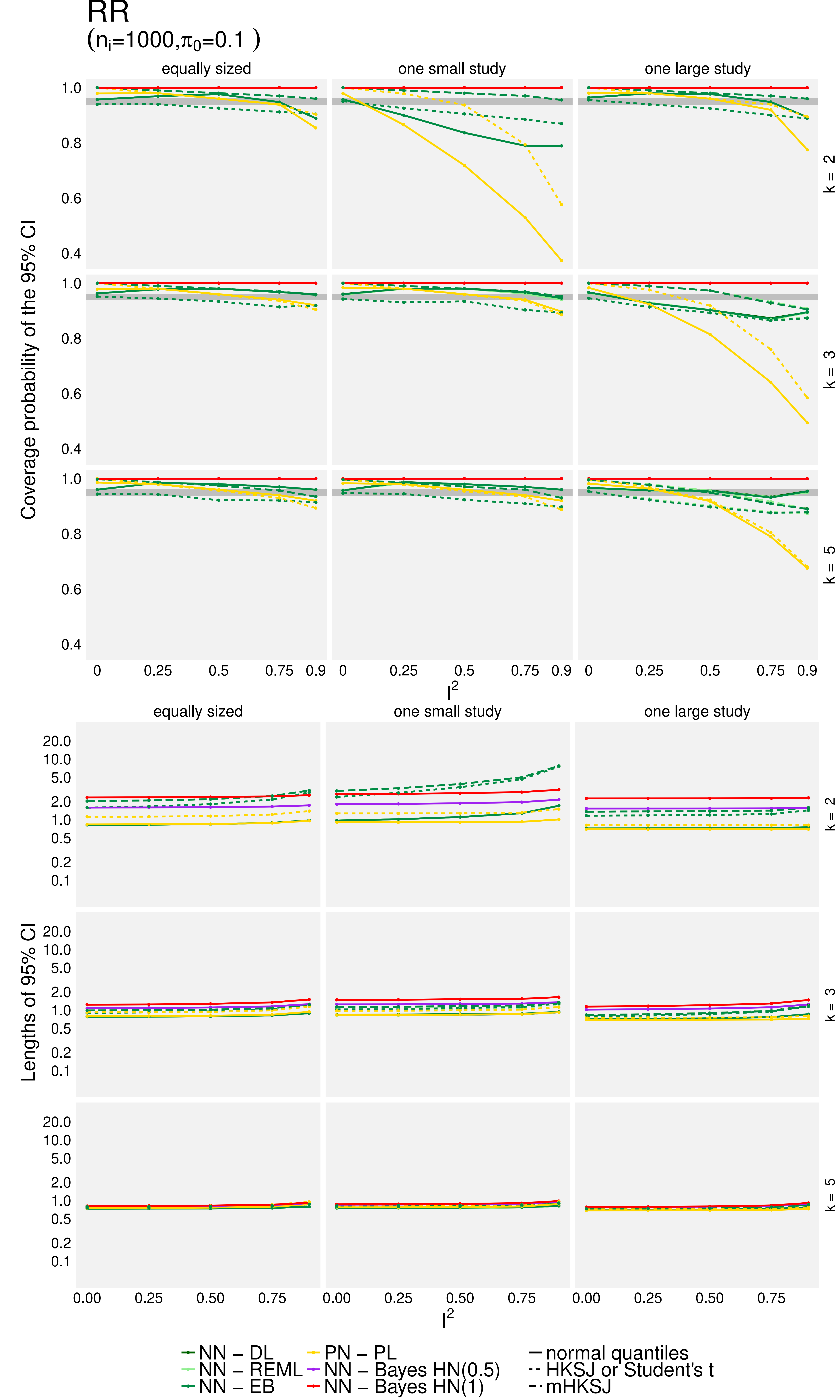

RR
(n_i=1000,π_0=0.1)
equally sized
one small study
one large study
Coverage probability of the 95% CI
k = 2
k = 3
k = 5
1.0
0.8
0.6
0.4
0
0.25
0.5
0.75
0.9
I^2
Lengths of 95% CI
20.0
10.0
5.0
2.0
1.0
0.5
0.2
0.1
0.00
0.25
0.50
0.75
NN – DL
NN – REML
NN – EB
PN – PL
NN – Bayes HN(0.5)
NN – Bayes HN(1)
normal quantiles
HKSJ or Student's t
mHKSJ

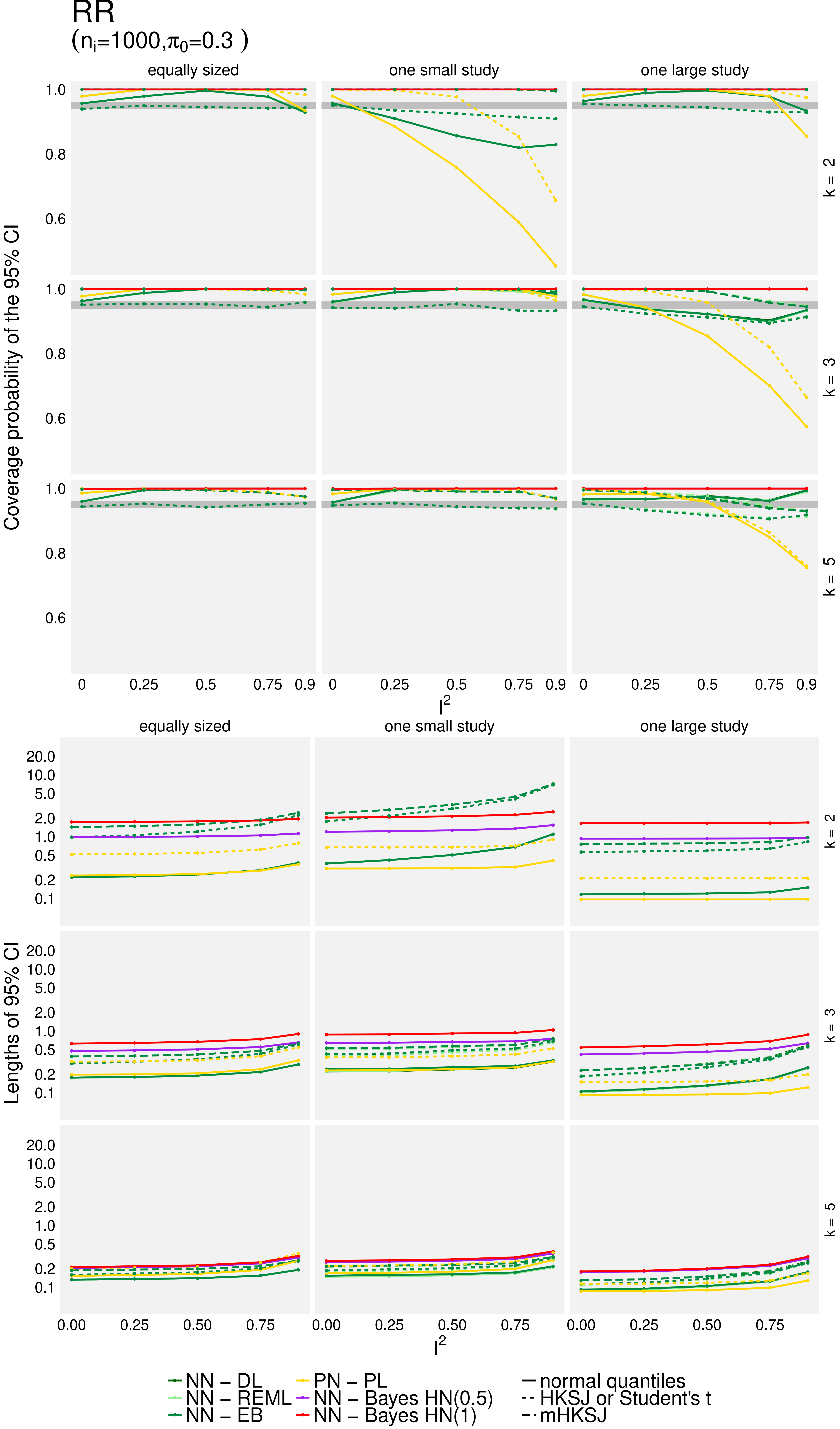

RR
(n_i=1000,π_0=0.3)
equally sized
one small study
one large study
Coverage probability of the 95% CI
k = 2
k = 3
k = 5
1.0
0.8
0.6
0
0.25
0.5
0.75
0.9
I^2
Lengths of 95% CI
20.0
10.0
5.0
2.0
1.0
0.5
0.2
0.1
0.00
0.25
0.50
0.75
NN – DL
NN – REML
NN – EB
PN – PL
NN – Bayes HN(0.5)
NN – Bayes HN(1)
normal quantiles
HKSJ or Student's t
mHKSJ

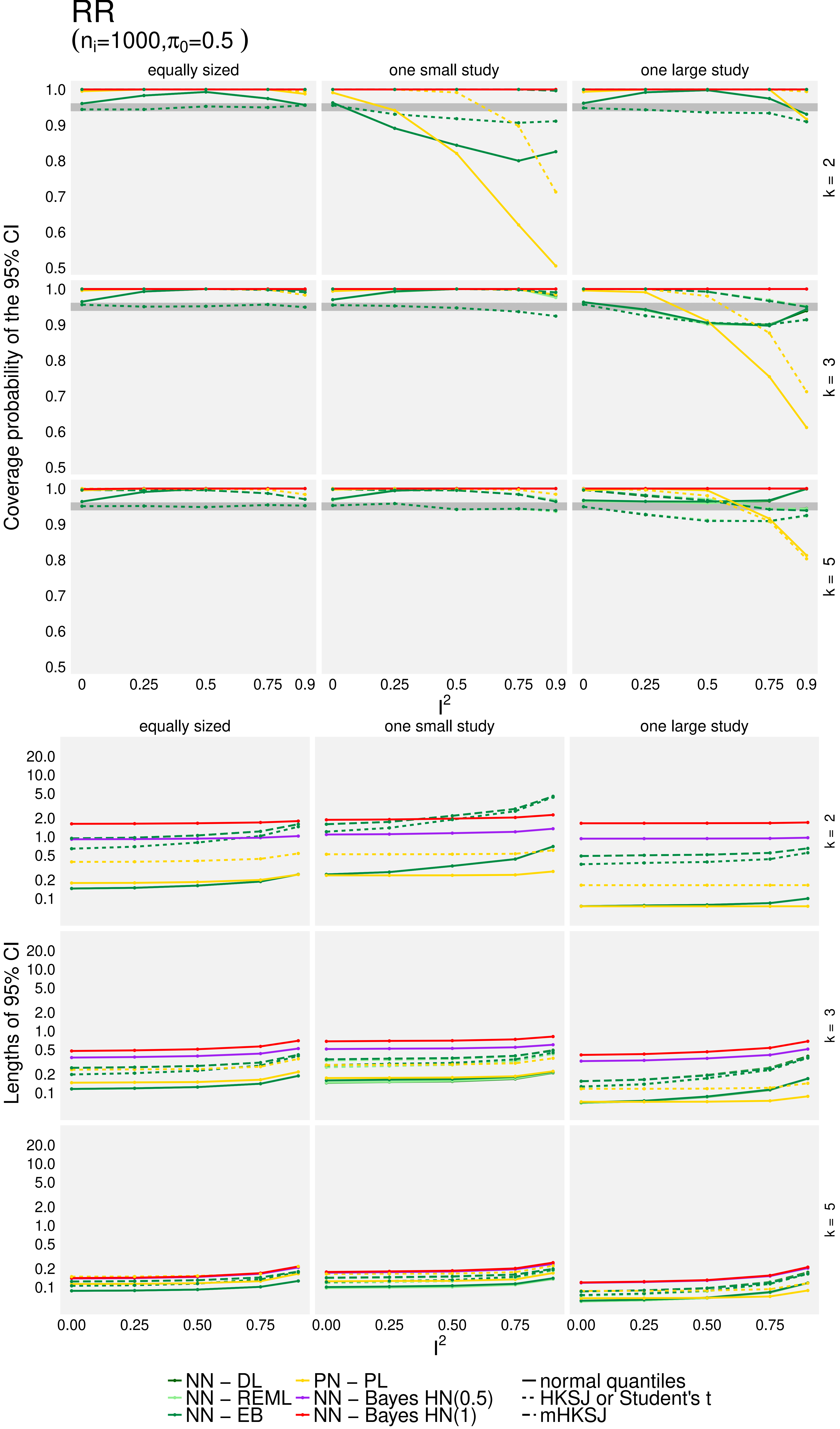

RR
(n_i=1000,π_0=0.5)
equally sized
one small study
one large study
Coverage probability of the 95% CI
k = 2
k = 3
k = 5
1.0
0.9
0.8
0.7
0.6
0.5
0
0.25
0.5
0.75
0.9
I^2
Lengths of 95% CI
20.0
10.0
5.0
2.0
1.0
0.5
0.2
0.1
0.00
0.25
0.50
0.75
NN – DL
NN – REML
NN – EB
PN – PL
NN – Bayes HN(0.5)
NN – Bayes HN(1)
normal quantiles
HKSJ or Student's t
mHKSJ

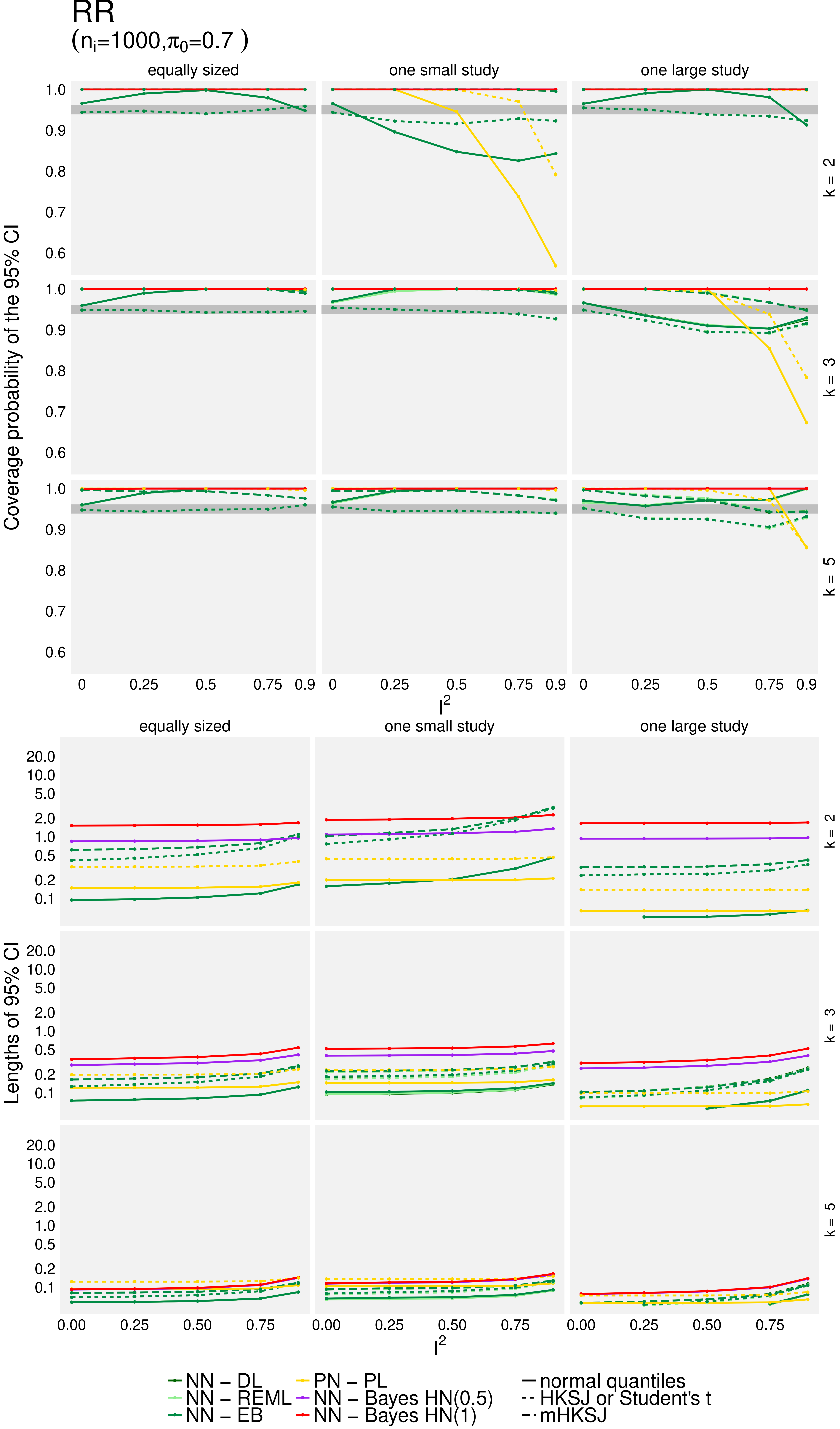
RR
(n_i=1000,π_0=0.7)
equally sized
one small study
one large study
Coverage probability of the 95% CI
k = 2
k = 3
k = 5
I^2
Lengths of 95% CI
NN – DL
NN – REML
NN – EB
PN – PL
NN – Bayes HN(0.5)
NN – Bayes HN(1)
normal quantiles
HKSJ or Student's t
mHKSJ

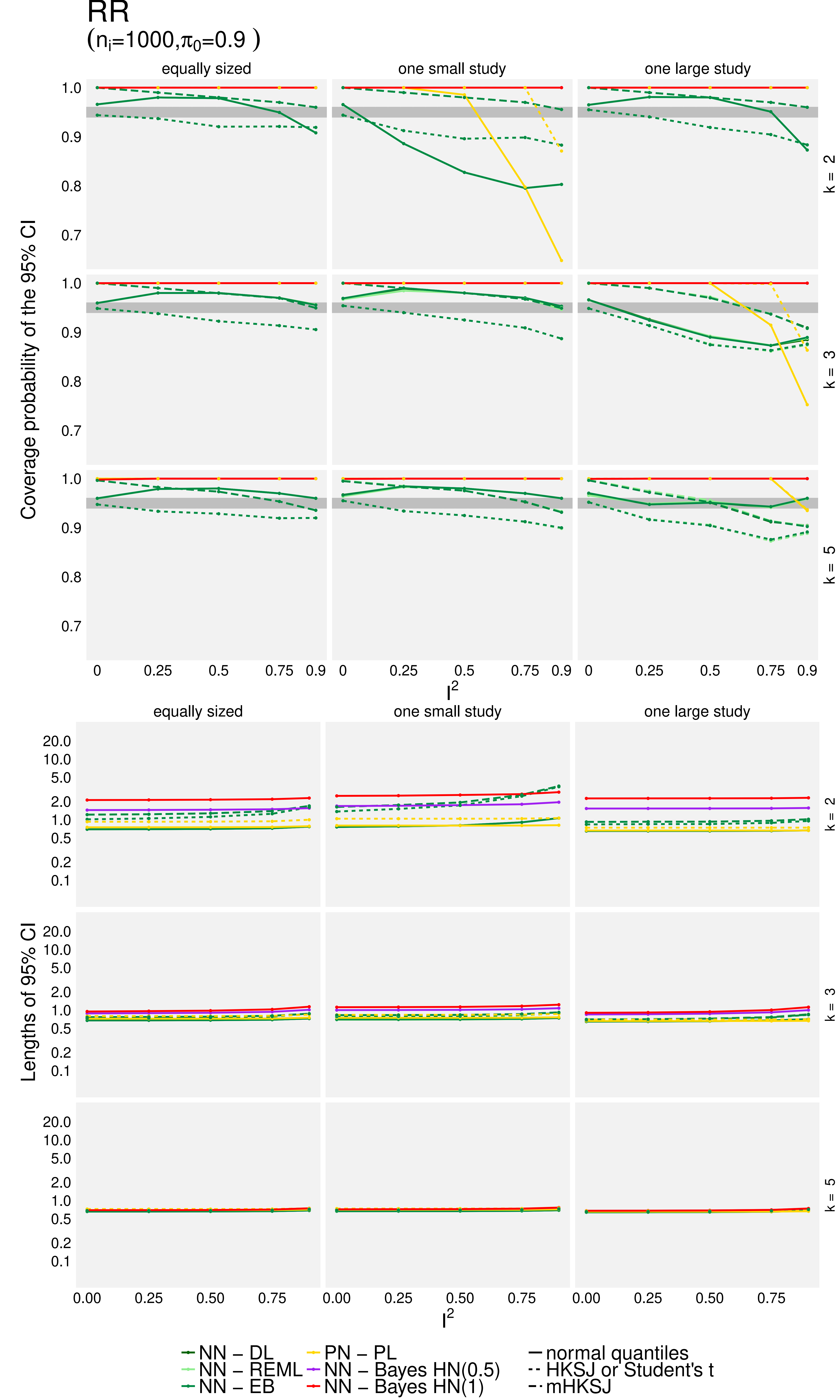

RR
(n_i=1000,π_0=0.9)
equally sized
one small study
one large study
Coverage probability of the 95% CI
k = 2
k = 3
k = 5
1.0
0.9
0.8
0.7
0
0.25
0.5
0.75
0.9
I^2
Lengths of 95% CI
20.0
10.0
5.0
2.0
1.0
0.5
0.2
0.1
0.00
0.25
0.50
0.75
NN – DL
NN – REML
NN – EB
PN – PL
NN – Bayes HN(0.5)
NN – Bayes HN(1)
normal quantiles
HKSJ or Student's t
mHKSJ
