# Supplementary material for: Likelihood-based random-effects meta-analysis with few studies: empirical and simulation studies
Source: BMC Med Res Methodol. 2019 Jan 11;19:16. doi: 10.1186/s12874-018-0618-3 (PMC6330405; doi:10.1186/s12874-018-0618-3)
Supplement: Supplementary file 3 — Supplement-2.pdf: The plots analogous to Fig. 5, for all simulation scenarios. (PDF 826 kb) [file 12874_2018_618_MOESM3_ESM.pdf]

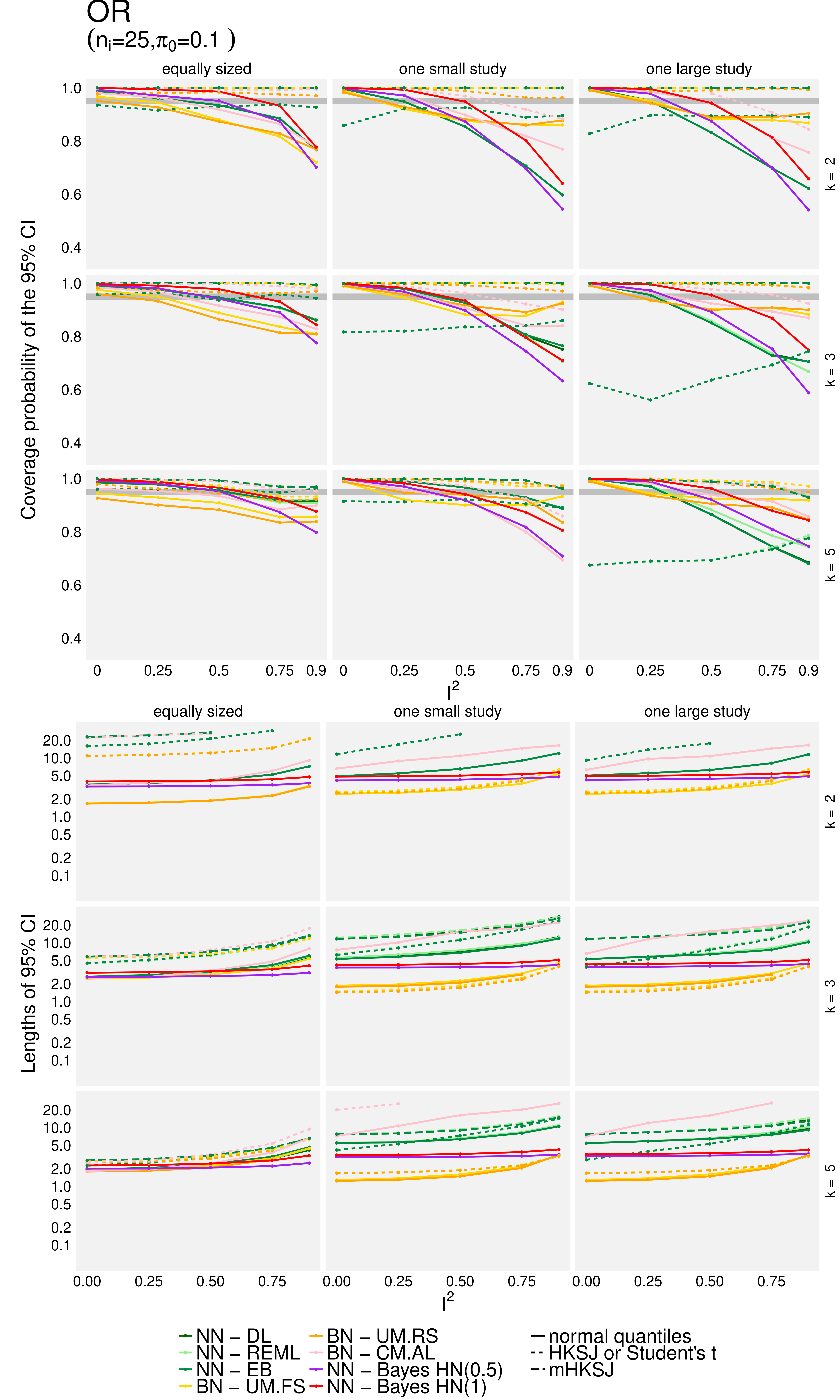

OR
(n_i=25,π_0=0.1)
equally sized
one small study
one large study
Coverage probability of the 95% CI
Lengths of 95% CI
k = 2
k = 3
k = 5
I^2
NN – DL
NN – REML
NN – EB
BN – UM.FS
BN – UM.RS
BN – CM.AL
NN – Bayes HN(0.5)
NN – Bayes HN(1)
normal quantiles
HKSJ or Student's t
mHKSJ

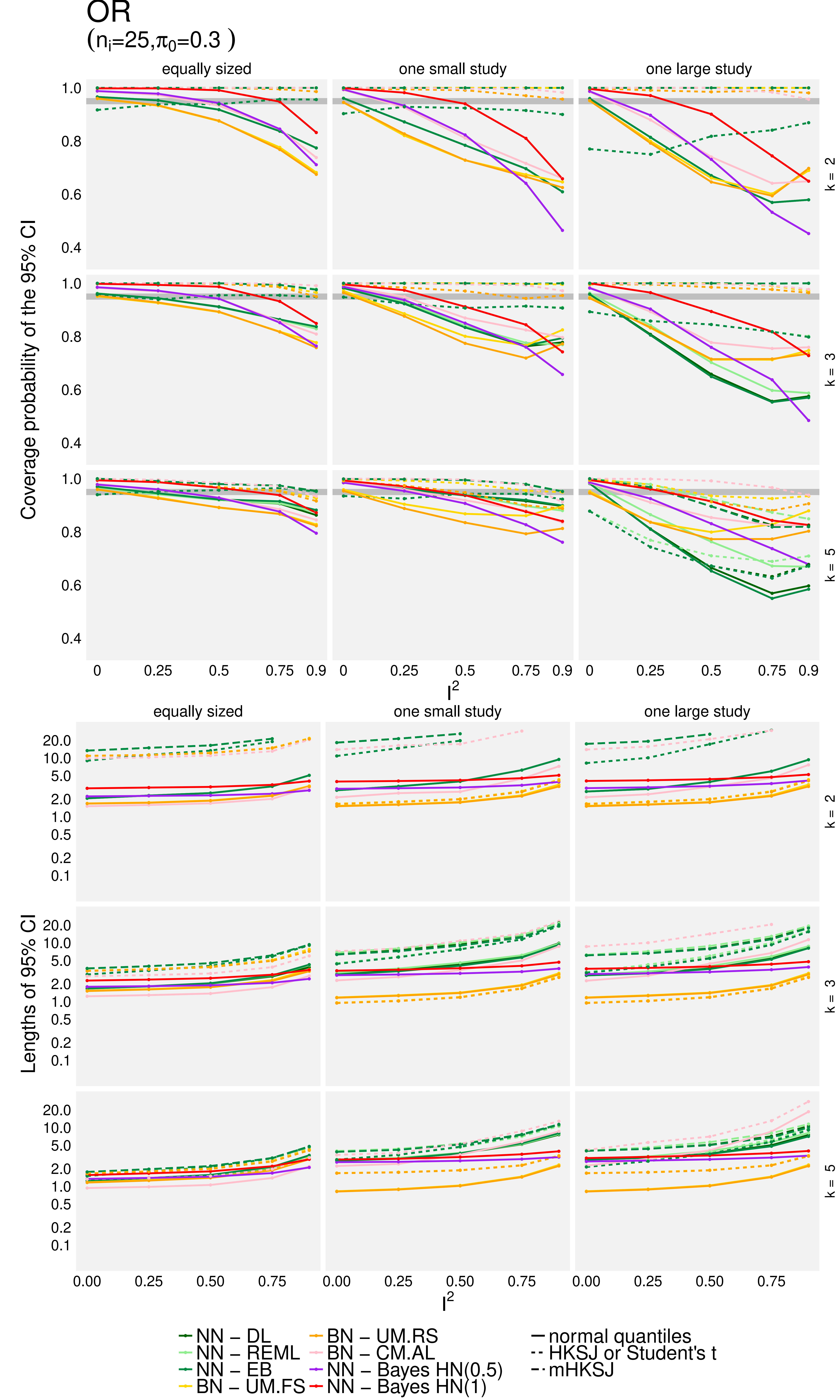

OR
(n_i=25,π_0=0.3)
equally sized
one small study
one large study
Coverage probability of the 95% CI
k = 2
k = 3
k = 5
1.0
0.8
0.6
0.4
0
0.25
0.5
0.75
0.9
I^2
Lengths of 95% CI
20.0
10.0
5.0
2.0
1.0
0.5
0.2
0.1
0.00
0.25
0.50
0.75
NN – DL
NN – REML
NN – EB
BN – UM.FS
BN – UM.RS
BN – CM.AL
NN – Bayes HN(0.5)
NN – Bayes HN(1)
normal quantiles
HKSJ or Student's t
mHKSJ

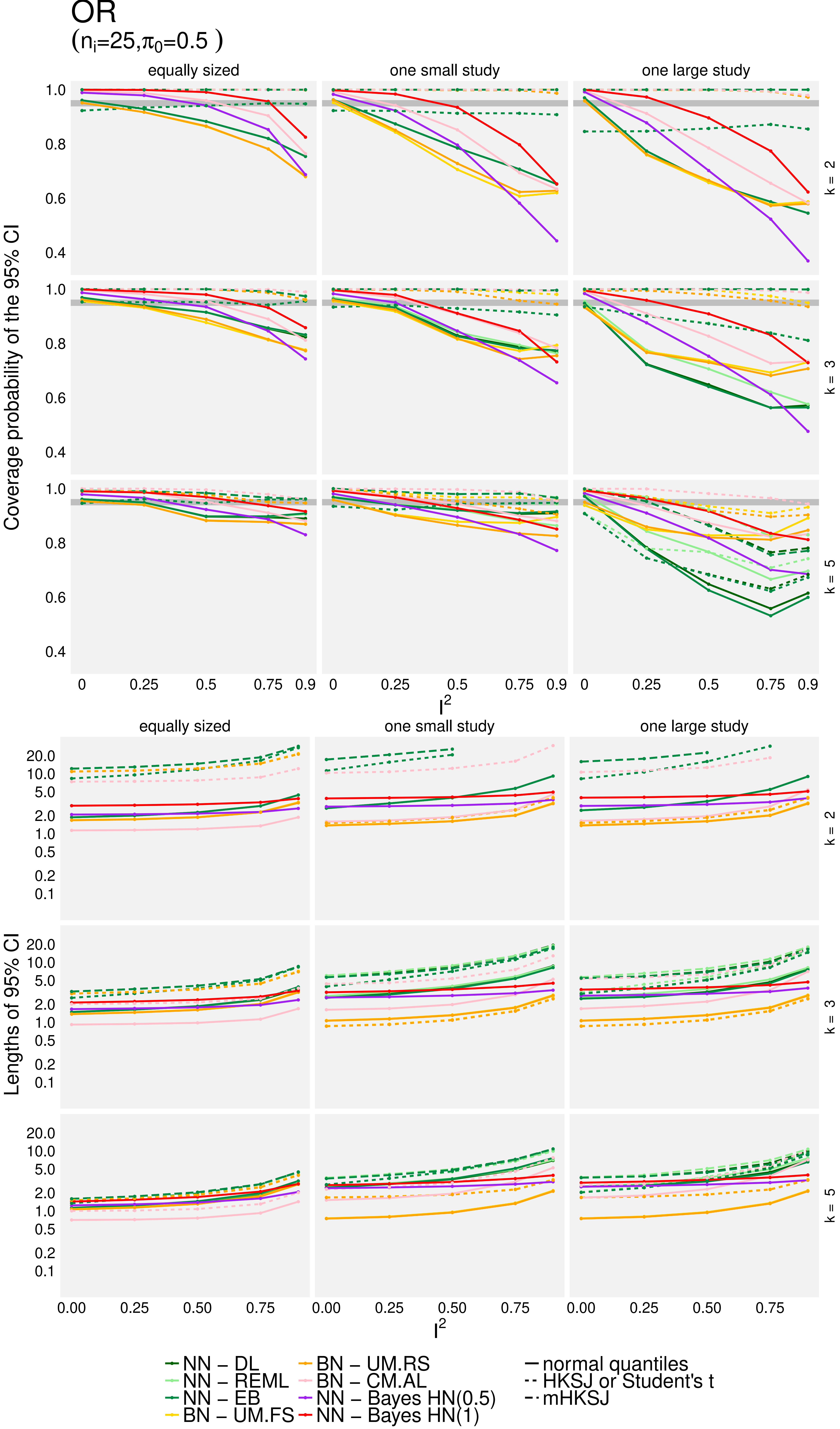

OR
(n_i=25,π_0=0.5)
equally sized
one small study
one large study
Coverage probability of the 95% CI
k = 2
k = 3
k = 5
I^2
Lengths of 95% CI
NN – DL
NN – REML
NN – EB
BN – UM.FS
BN – UM.RS
BN – CM.AL
NN – Bayes HN(0.5)
NN – Bayes HN(1)
normal quantiles
HKSJ or Student's t
mHKSJ

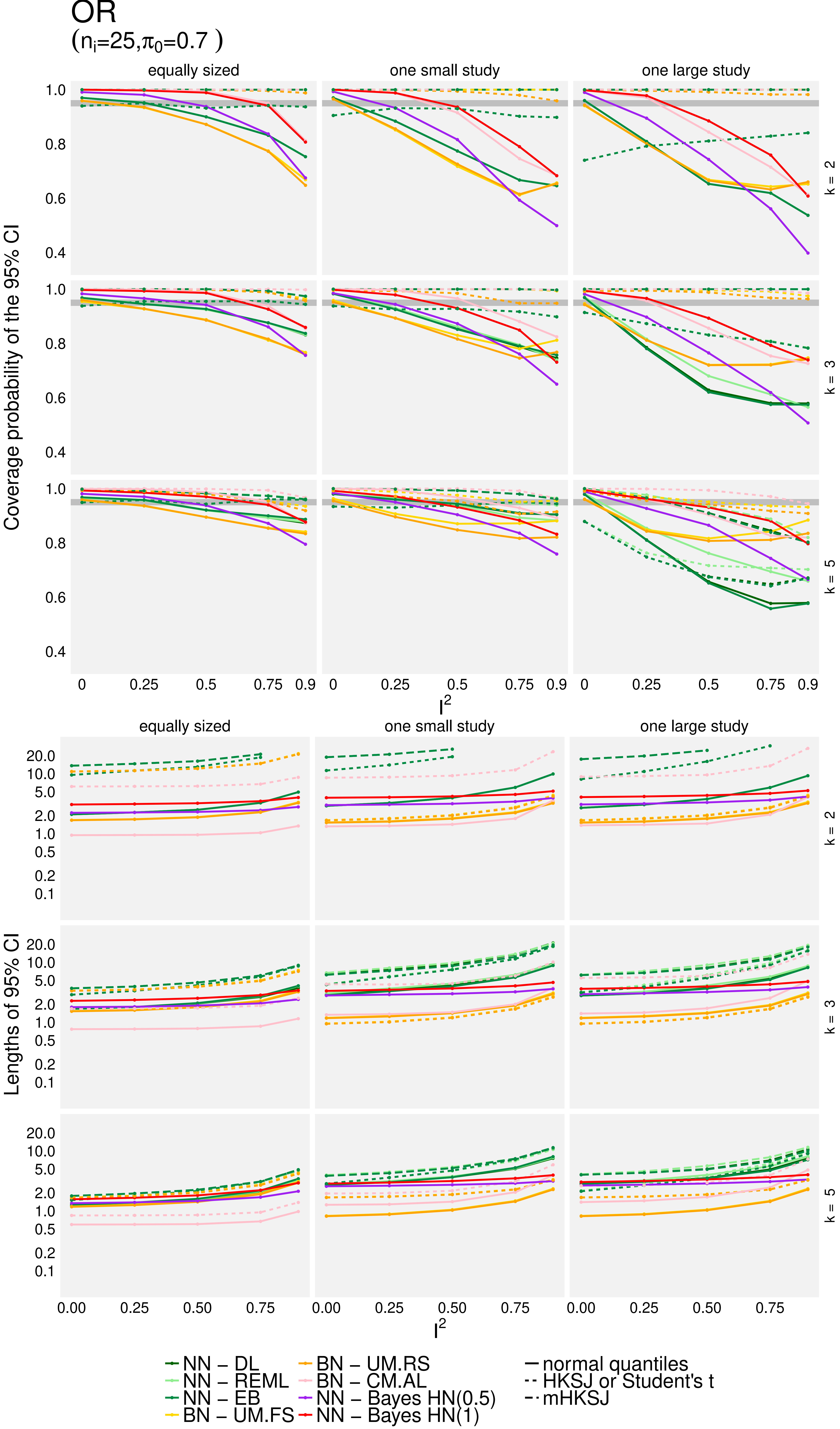

OR
(n_i=25,π_0=0.7)
equally sized
one small study
one large study
Coverage probability of the 95% CI
k = 2
k = 3
k = 5
I^2
Lengths of 95% CI
NN – DL
NN – REML
NN – EB
BN – UM.FS
BN – UM.RS
BN – CM.AL
NN – Bayes HN(0.5)
NN – Bayes HN(1)
normal quantiles
HKSJ or Student's t
mHKSJ

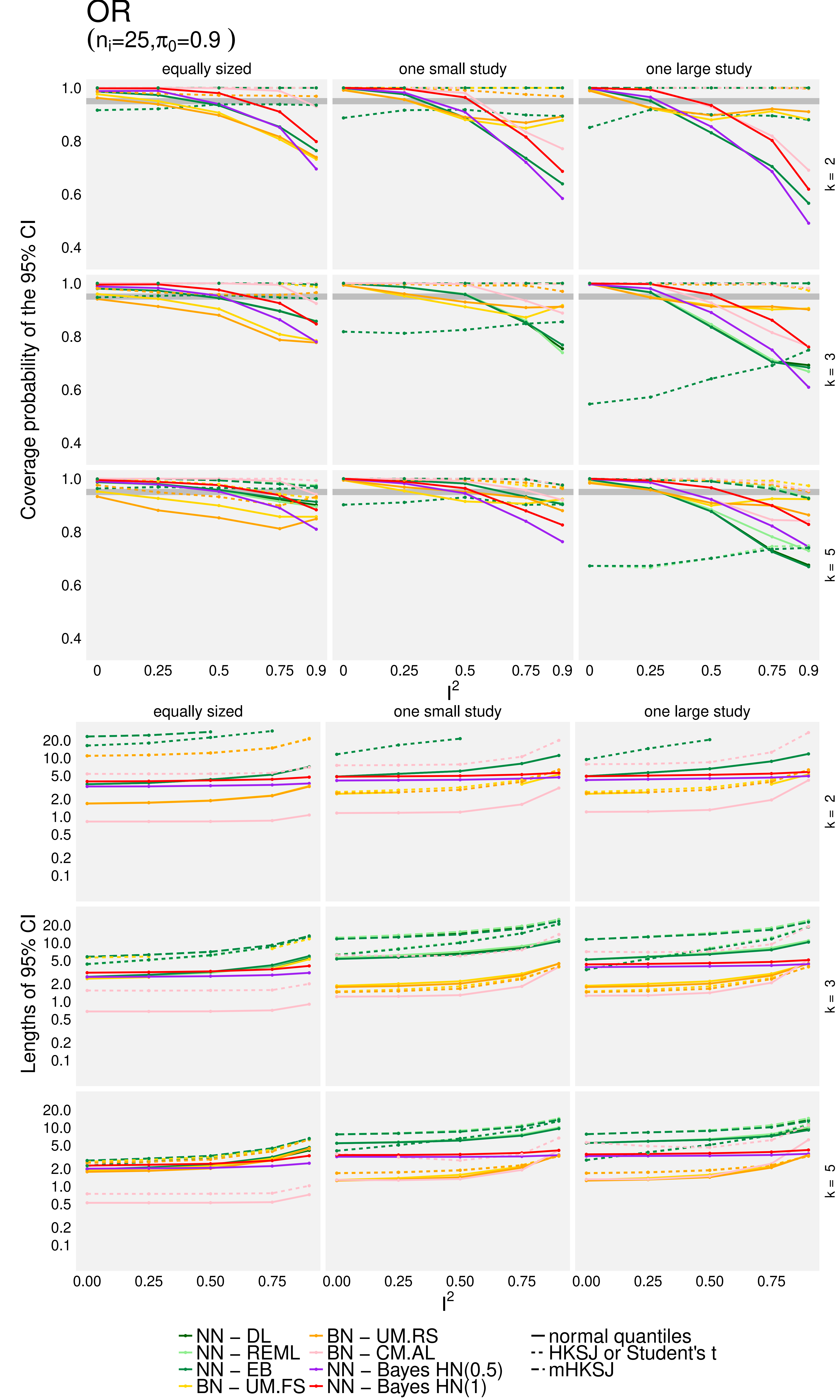
OR
(n_i=25,π_0=0.9)
equally sized
one small study
one large study
Coverage probability of the 95% CI
k = 2
k = 3
k = 5
1.0
0.8
0.6
0.4
0 0.25 0.5 0.75 0.9
I^2
Lengths of 95% CI
20.0
10.0
5.0
2.0
1.0
0.5
0.2
0.1
0.00 0.25 0.50 0.75
NN – DL
NN – REML
NN – EB
BN – UM.FS
BN – UM.RS
BN – CM.AL
NN – Bayes HN(0.5)
NN – Bayes HN(1)
normal quantiles
HKSJ or Student's t
mHKSJ

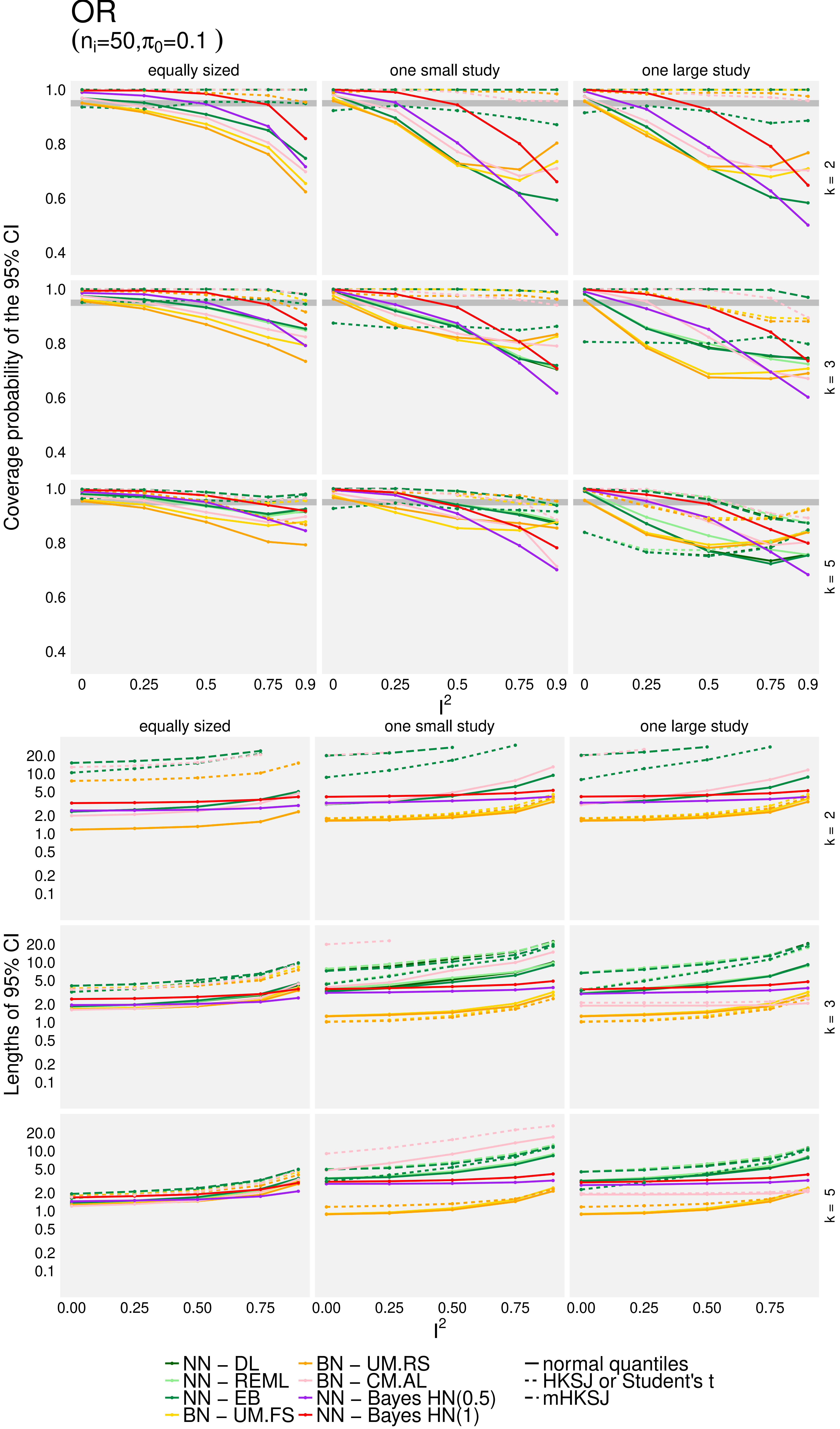
OR
(n_i=50,π_0=0.1)
equally sized
one small study
one large study
Coverage probability of the 95% CI
k = 2
k = 3
k = 5
I^2
Lengths of 95% CI
NN – DL
NN – REML
NN – EB
BN – UM.FS
BN – UM.RS
BN – CM.AL
NN – Bayes HN(0.5)
NN – Bayes HN(1)
normal quantiles
HKSJ or Student's t
mHKSJ

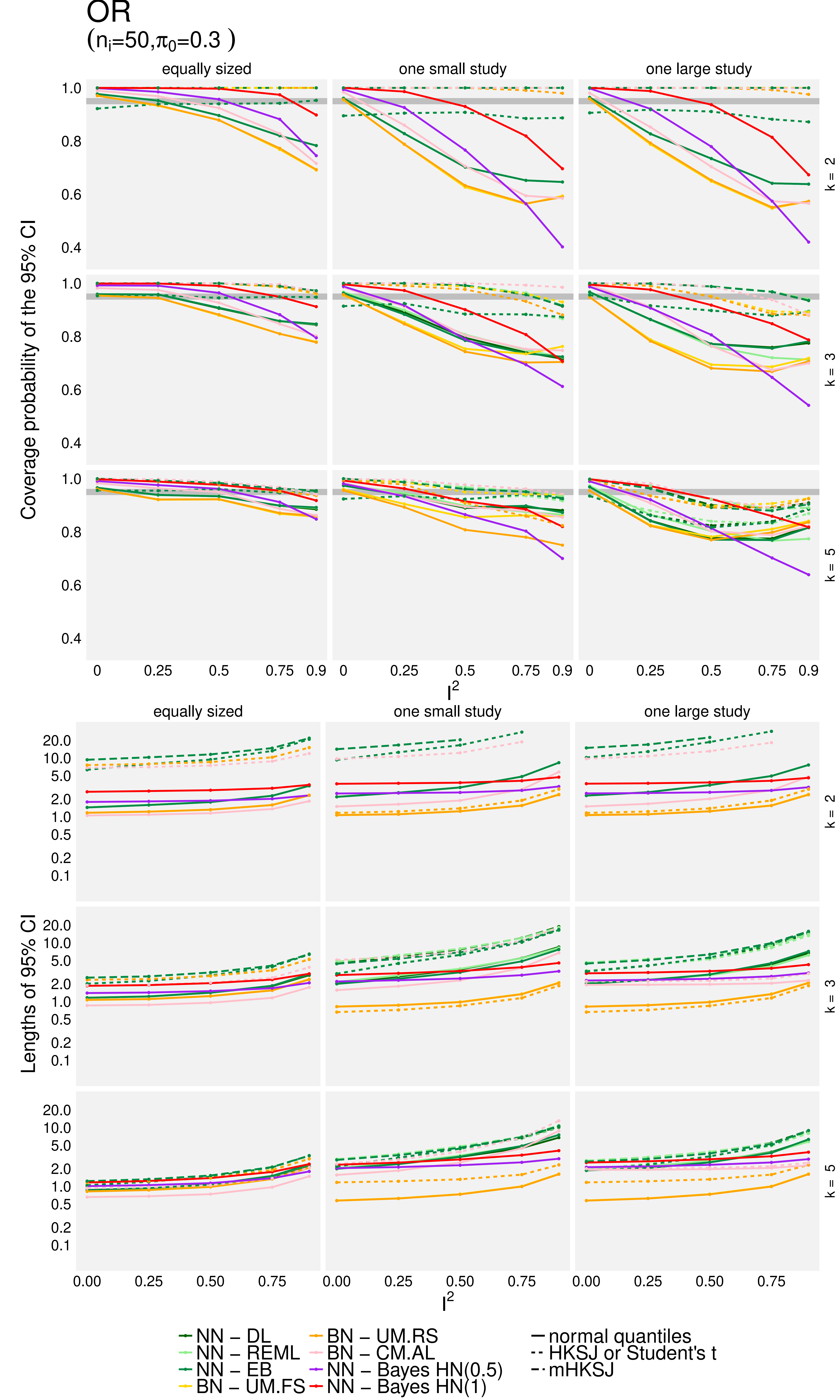
OR
(n_i=50,π_0=0.3)
equally sized
one small study
one large study
Coverage probability of the 95% CI
1.0
0.8
0.6
0.4
k = 2
k = 3
k = 5
0
0.25
0.5
0.75
0.9
I^2
Lengths of 95% CI
20.0
10.0
5.0
2.0
1.0
0.5
0.2
0.1
0.00
0.25
0.50
0.75
NN – DL
NN – REML
NN – EB
BN – UM.FS
BN – UM.RS
BN – CM.AL
NN – Bayes HN(0.5)
NN – Bayes HN(1)
normal quantiles
HKSJ or Student's t
mHKSJ

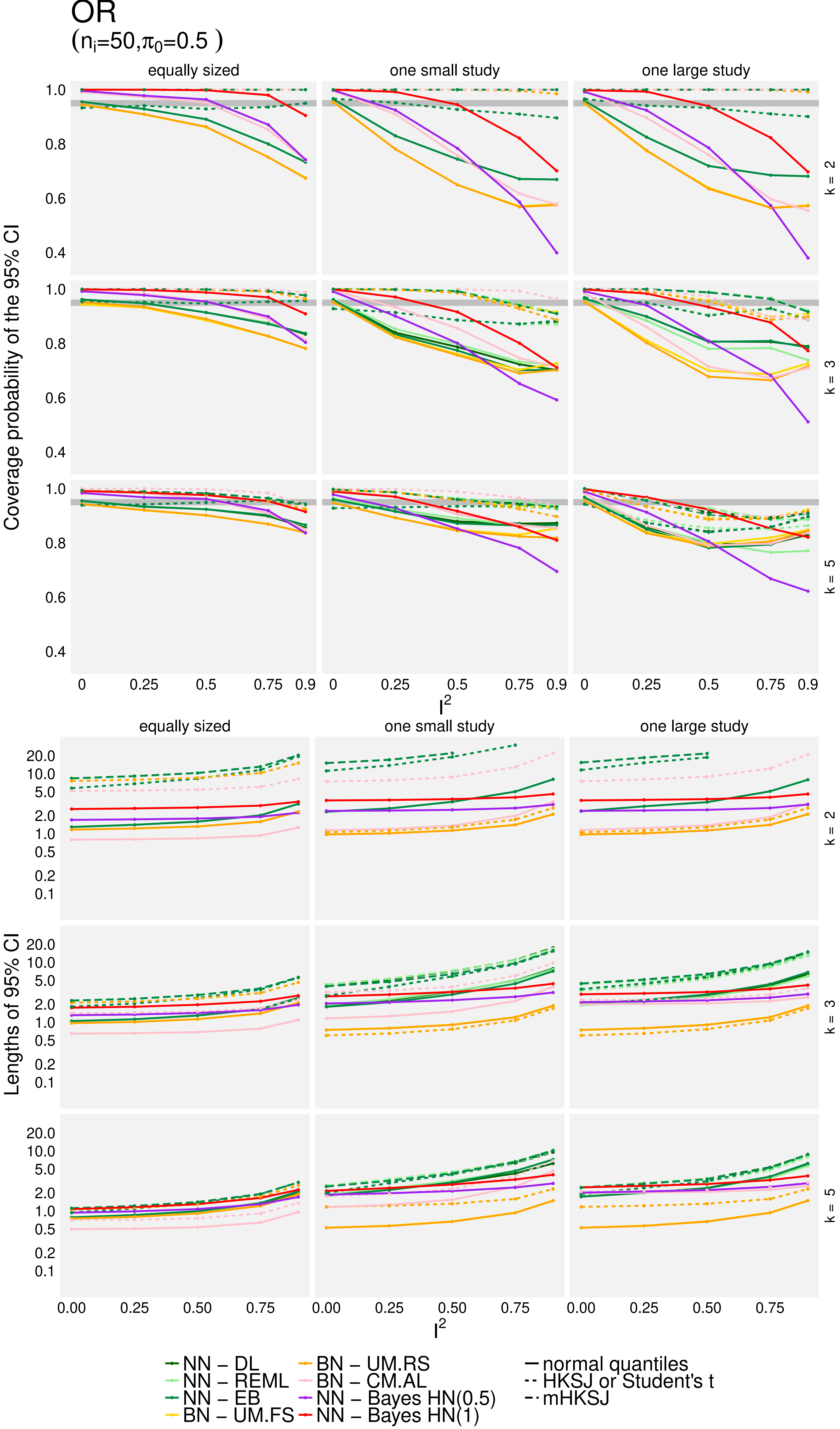

OR
(n_i=50,π_0=0.5)
equally sized
one small study
one large study
Coverage probability of the 95% CI
k = 2
k = 3
k = 5
I²
Lengths of 95% CI
NN – DL
NN – REML
NN – EB
BN – UM.FS
BN – UM.RS
BN – CM.AL
NN – Bayes HN(0.5)
NN – Bayes HN(1)
normal quantiles
HKSJ or Student's t
mHKSJ

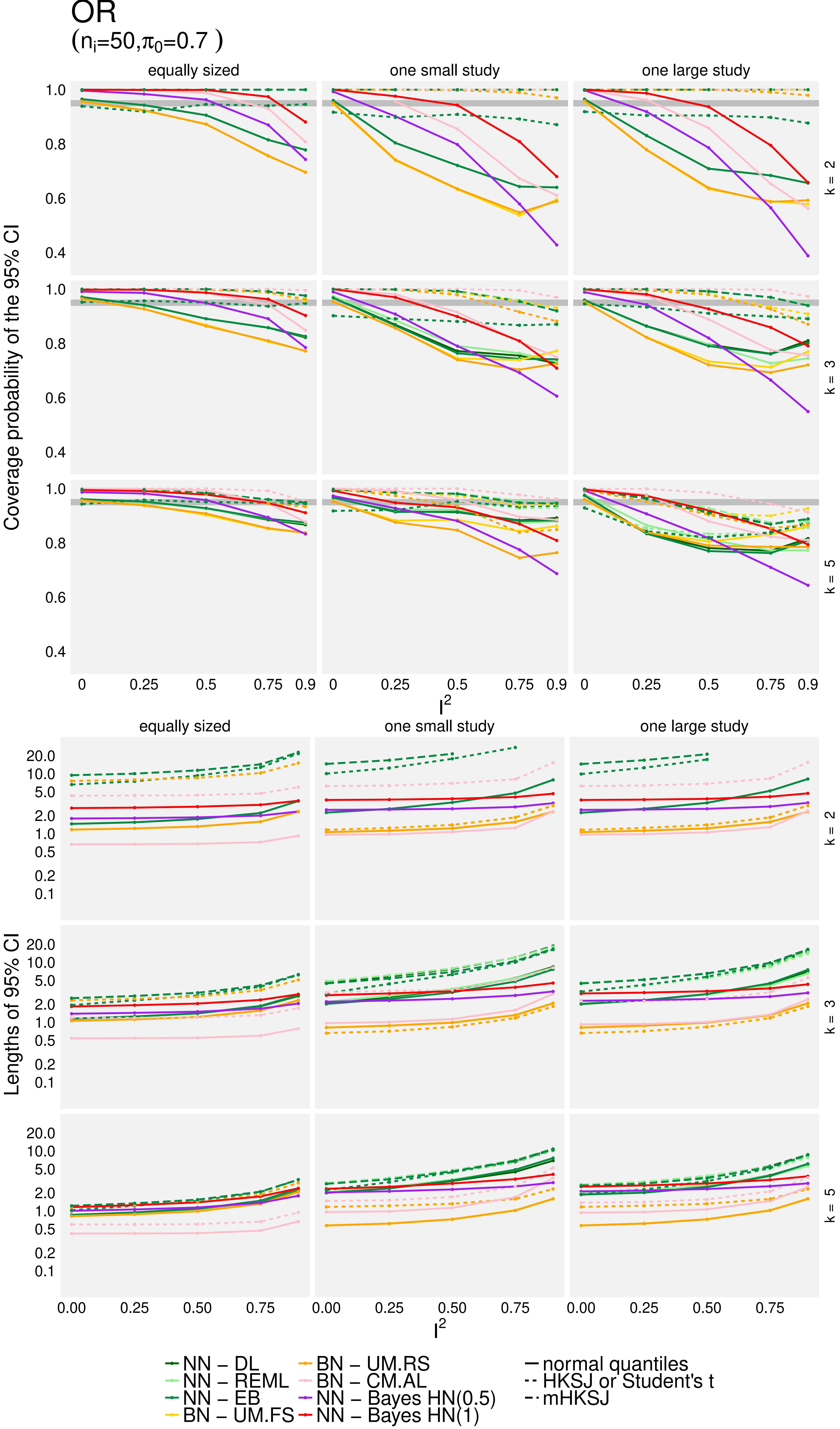

OR
(n_i=50,π_0=0.7)
equally sized
one small study
one large study
Coverage probability of the 95% CI
Lengths of 95% CI
k = 2
k = 3
k = 5
I^2
NN – DL
NN – REML
NN – EB
BN – UM.FS
BN – UM.RS
BN – CM.AL
NN – Bayes HN(0.5)
NN – Bayes HN(1)
normal quantiles
HKSJ or Student's t
mHKSJ

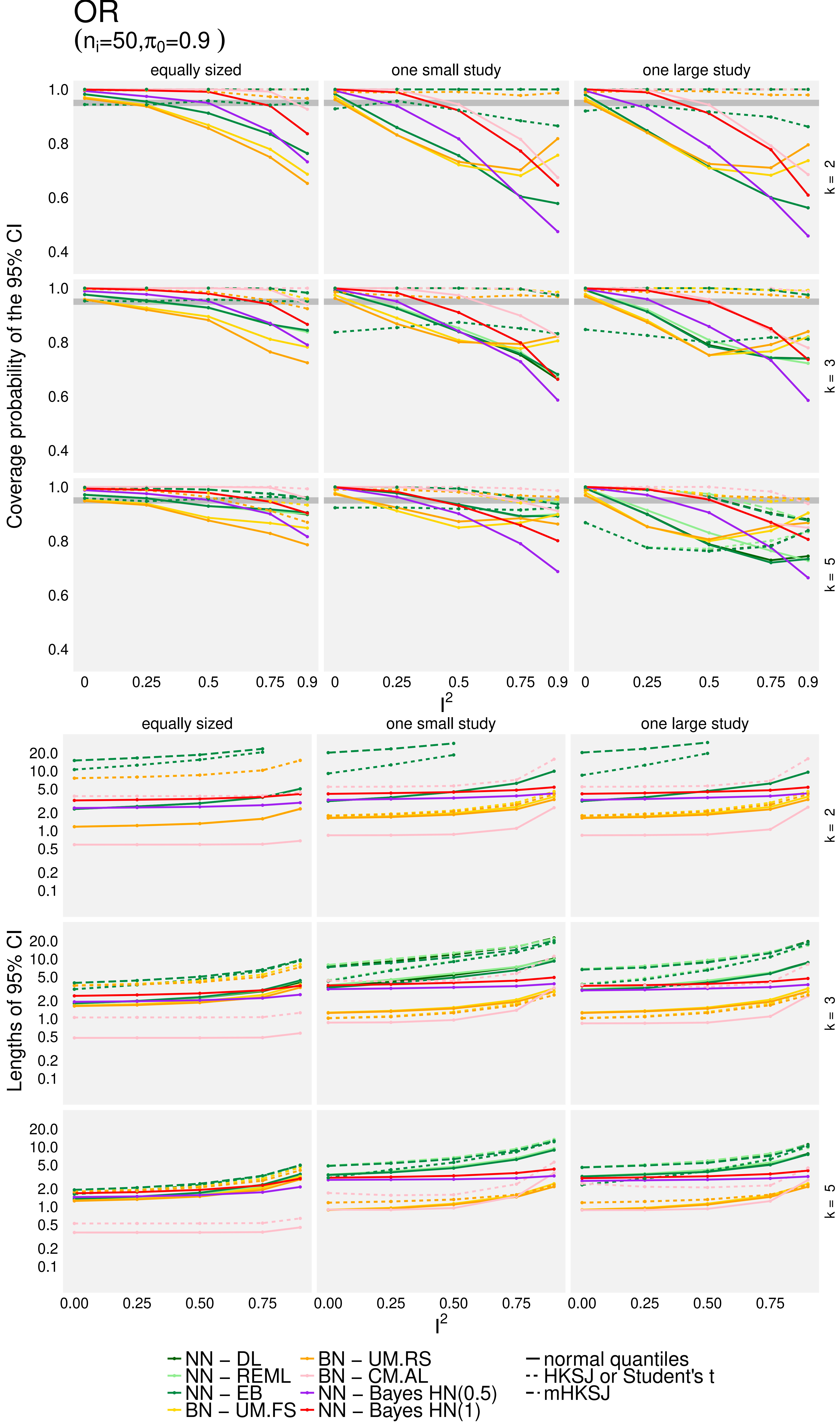
OR
(n_i=50, π_0=0.9)
equally sized
one small study
one large study
Coverage probability of the 95% CI
Lengths of 95% CI
k = 2
k = 3
k = 5
I^2
NN – DL
NN – REML
NN – EB
BN – UM.FS
BN – UM.RS
BN – CM.AL
NN – Bayes HN(0.5)
NN – Bayes HN(1)
normal quantiles
HKSJ or Student's t
mHKSJ

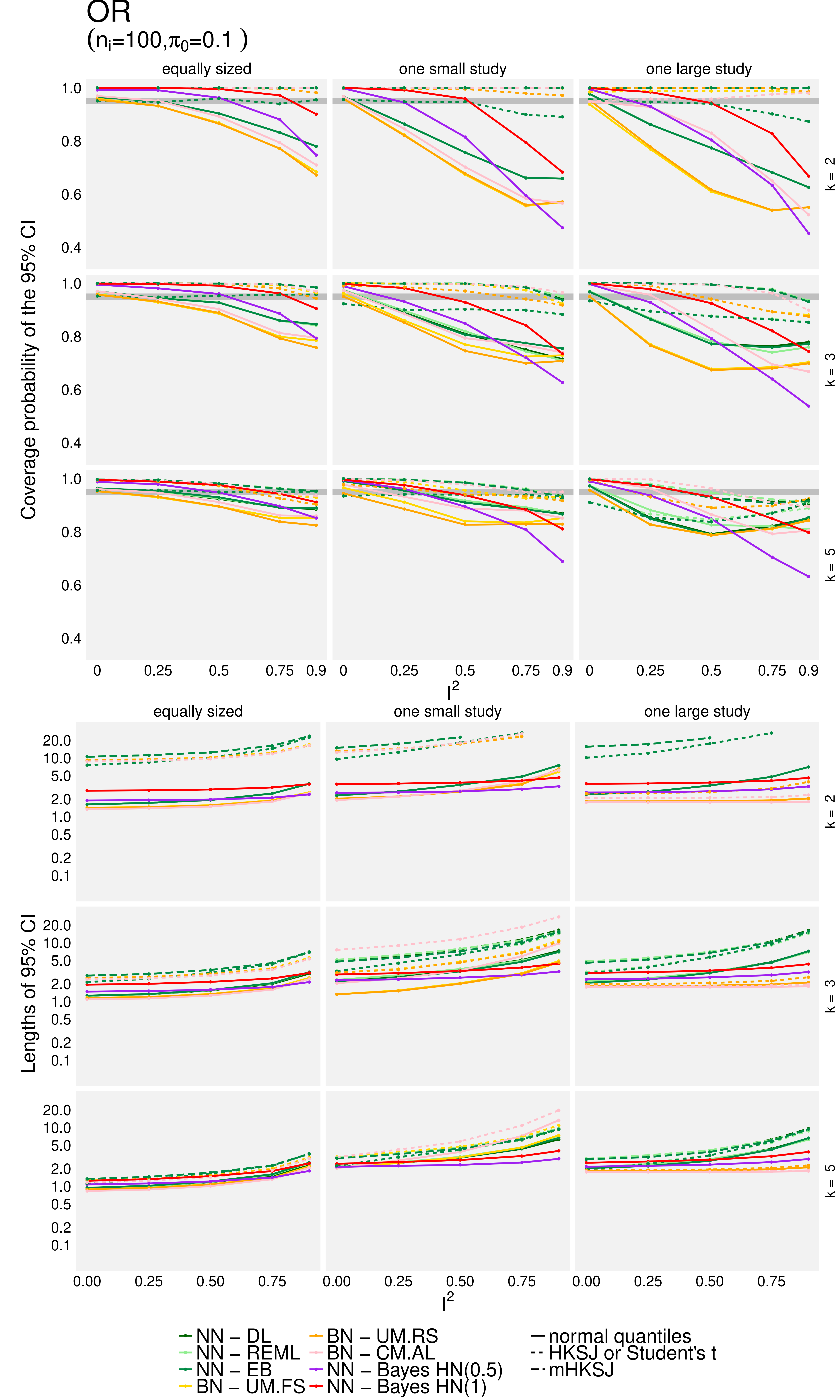
OR
(n_i=100,π_0=0.1)
equally sized
one small study
one large study
Coverage probability of the 95% CI
k = 2
k = 3
k = 5
I^2
Lengths of 95% CI
NN – DL
NN – REML
NN – EB
BN – UM.FS
BN – UM.RS
BN – CM.AL
NN – Bayes HN(0.5)
NN – Bayes HN(1)
normal quantiles
HKSJ or Student's t
mHKSJ

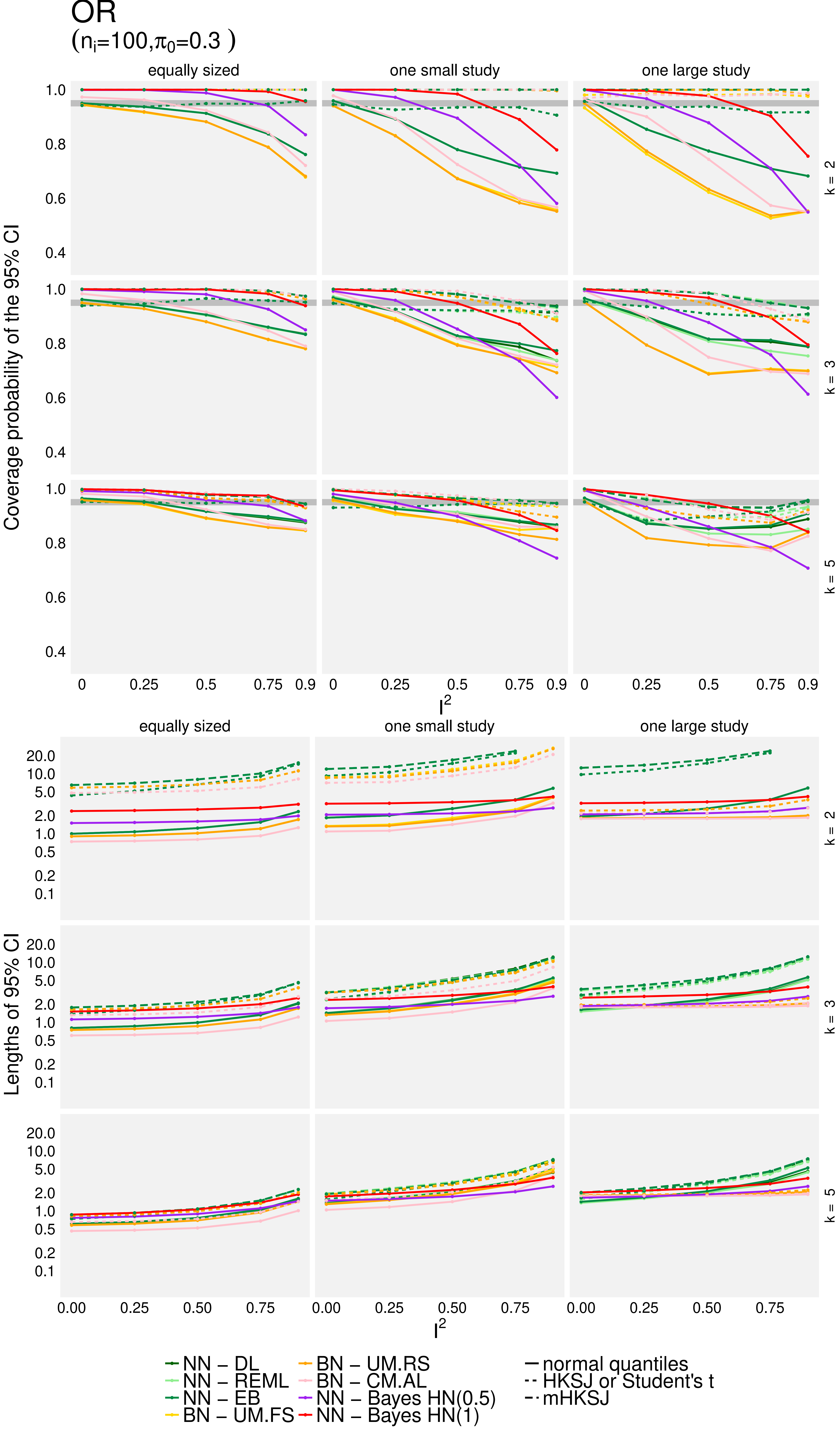
OR
(n_i=100,π_0=0.3)
equally sized
one small study
one large study
Coverage probability of the 95% CI
k = 2
k = 3
k = 5
I^2
Lengths of 95% CI
NN – DL
NN – REML
NN – EB
BN – UM.FS
BN – UM.RS
BN – CM.AL
NN – Bayes HN(0.5)
NN – Bayes HN(1)
normal quantiles
HKSJ or Student's t
mHKSJ

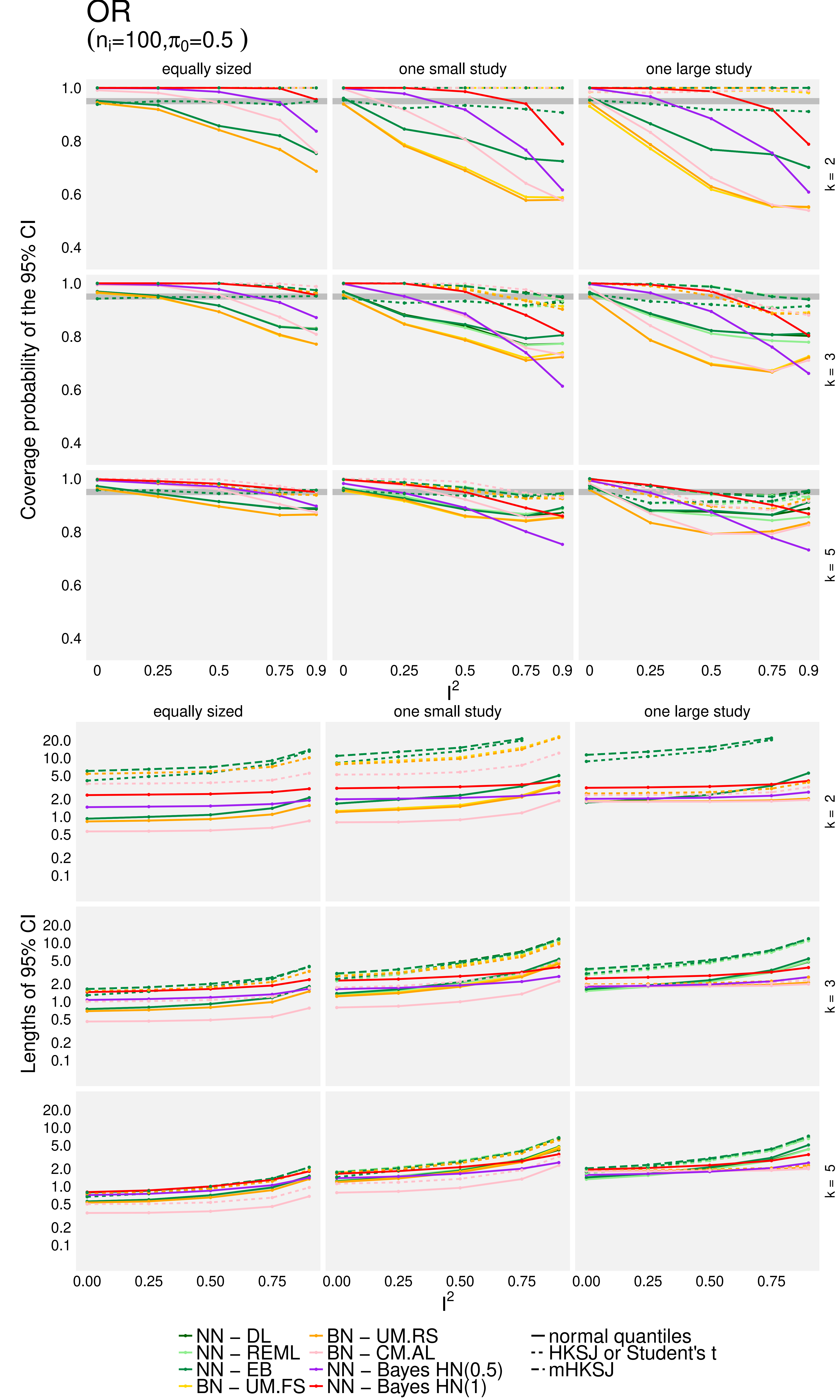

OR
(n_i=100,π_0=0.5)
equally sized
one small study
one large study
Coverage probability of the 95% CI
k = 2
k = 3
k = 5
I^2
Lengths of 95% CI
NN – DL
NN – REML
NN – EB
BN – UM.FS
BN – UM.RS
BN – CM.AL
NN – Bayes HN(0.5)
NN – Bayes HN(1)
normal quantiles
HKSJ or Student's t
mHKSJ

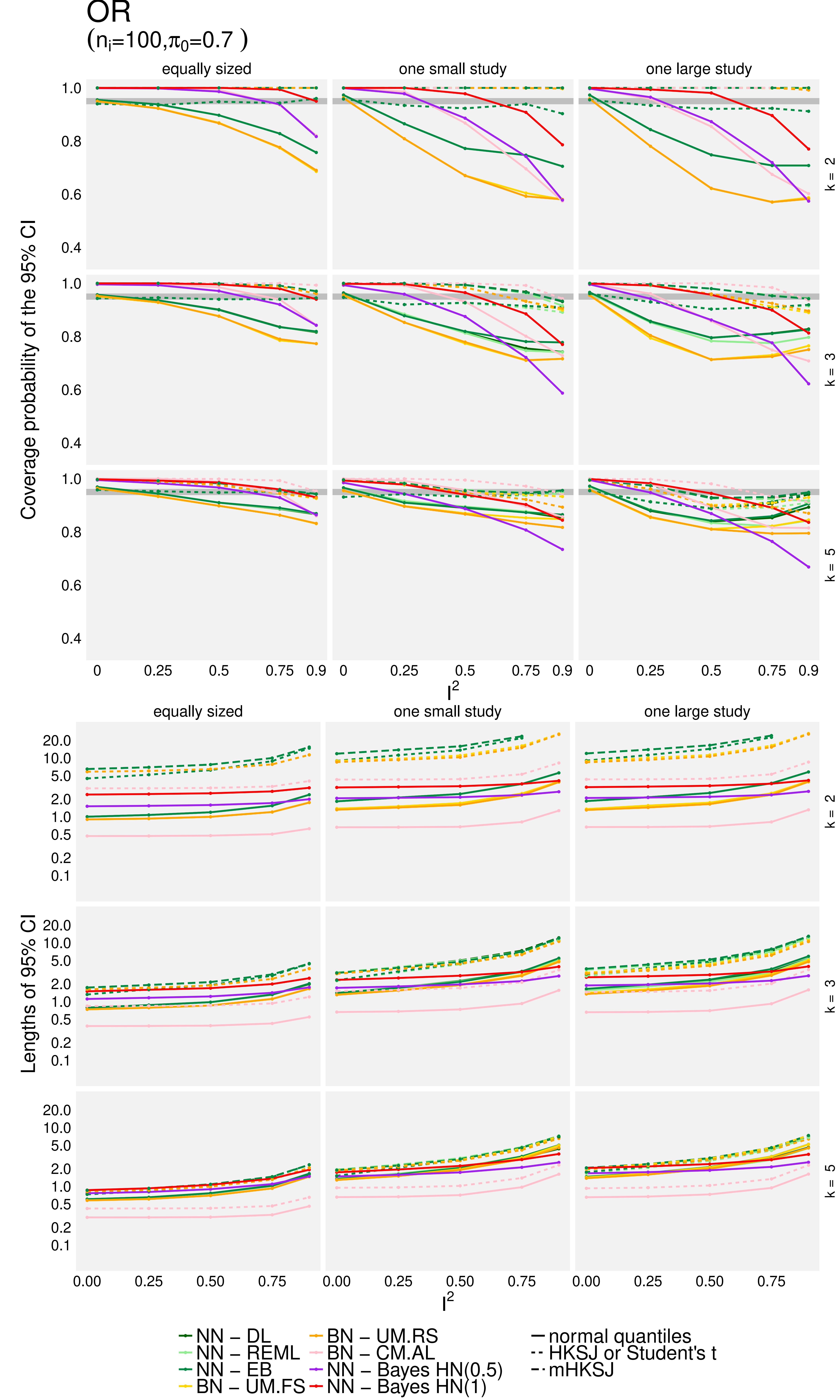

OR
(n_i=100,π_0=0.7)
equally sized
one small study
one large study
Coverage probability of the 95% CI
1.0
0.8
0.6
0.4
k = 2
k = 3
k = 5
0
0.25
0.5
0.75
0.9
I^2
Lengths of 95% CI
20.0
10.0
5.0
2.0
1.0
0.5
0.2
0.1
0.00
0.25
0.50
0.75
NN – DL
NN – REML
NN – EB
BN – UM.FS
BN – UM.RS
BN – CM.AL
NN – Bayes HN(0.5)
NN – Bayes HN(1)
normal quantiles
HKSJ or Student's t
mHKSJ

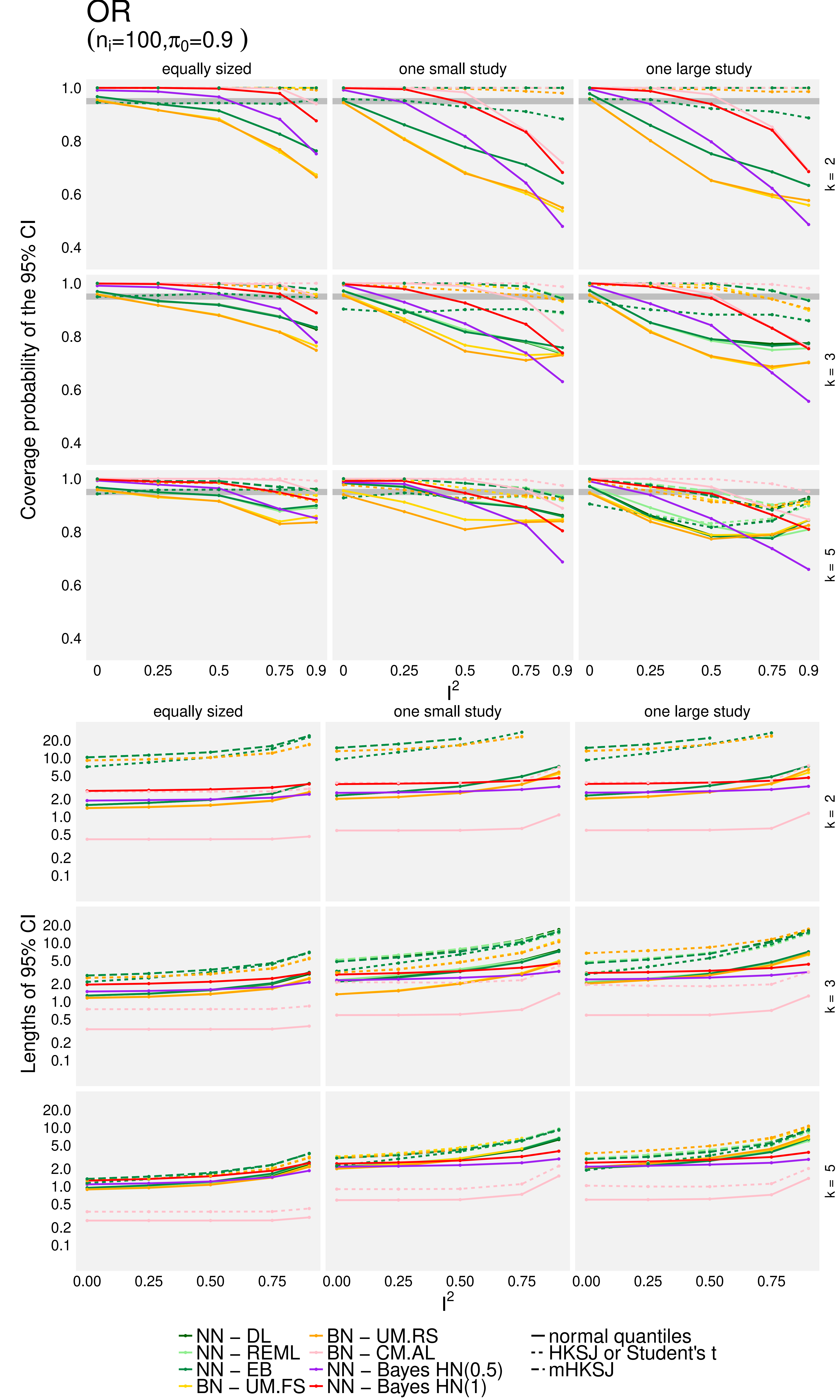

OR
(n_i=100,π_0=0.9)
equally sized
one small study
one large study
Coverage probability of the 95% CI
k = 2
k = 3
k = 5
I²
Lengths of 95% CI
NN – DL
NN – REML
NN – EB
BN – UM.FS
BN – UM.RS
BN – CM.AL
NN – Bayes HN(0.5)
NN – Bayes HN(1)
normal quantiles
HKSJ or Student's t
mHKSJ

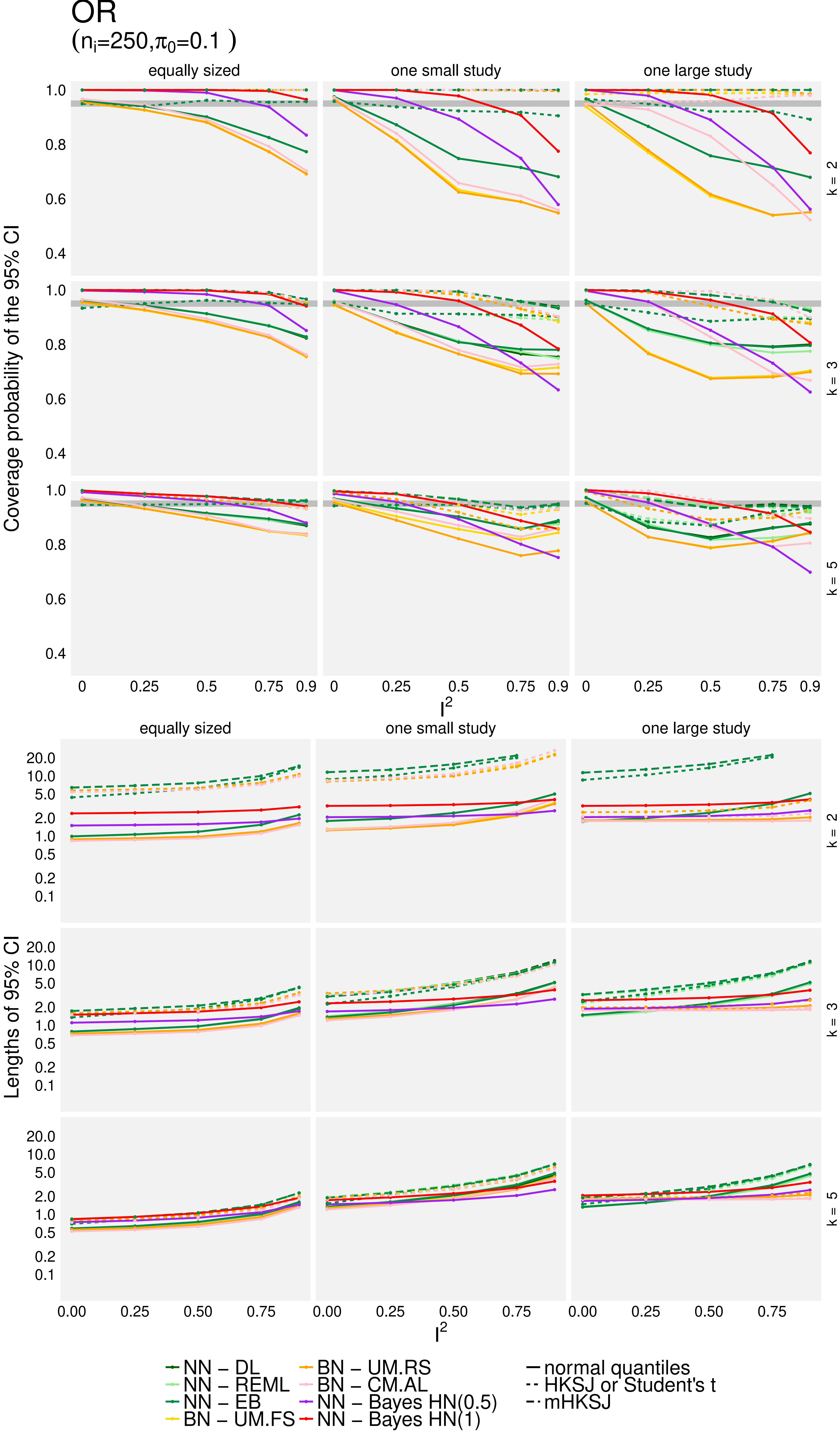
OR
(n_i=250,π_0=0.1)
equally sized
one small study
one large study
Coverage probability of the 95% CI
1.0
0.8
0.6
0.4
k = 2
k = 3
k = 5
0
0.25
0.5
0.75
0.9
I^2
Lengths of 95% CI
20.0
10.0
5.0
2.0
1.0
0.5
0.2
0.1
0.00
0.25
0.50
0.75
NN – DL
NN – REML
NN – EB
BN – UM.FS
BN – UM.RS
BN – CM.AL
NN – Bayes HN(0.5)
NN – Bayes HN(1)
normal quantiles
HKSJ or Student's t
mHKSJ

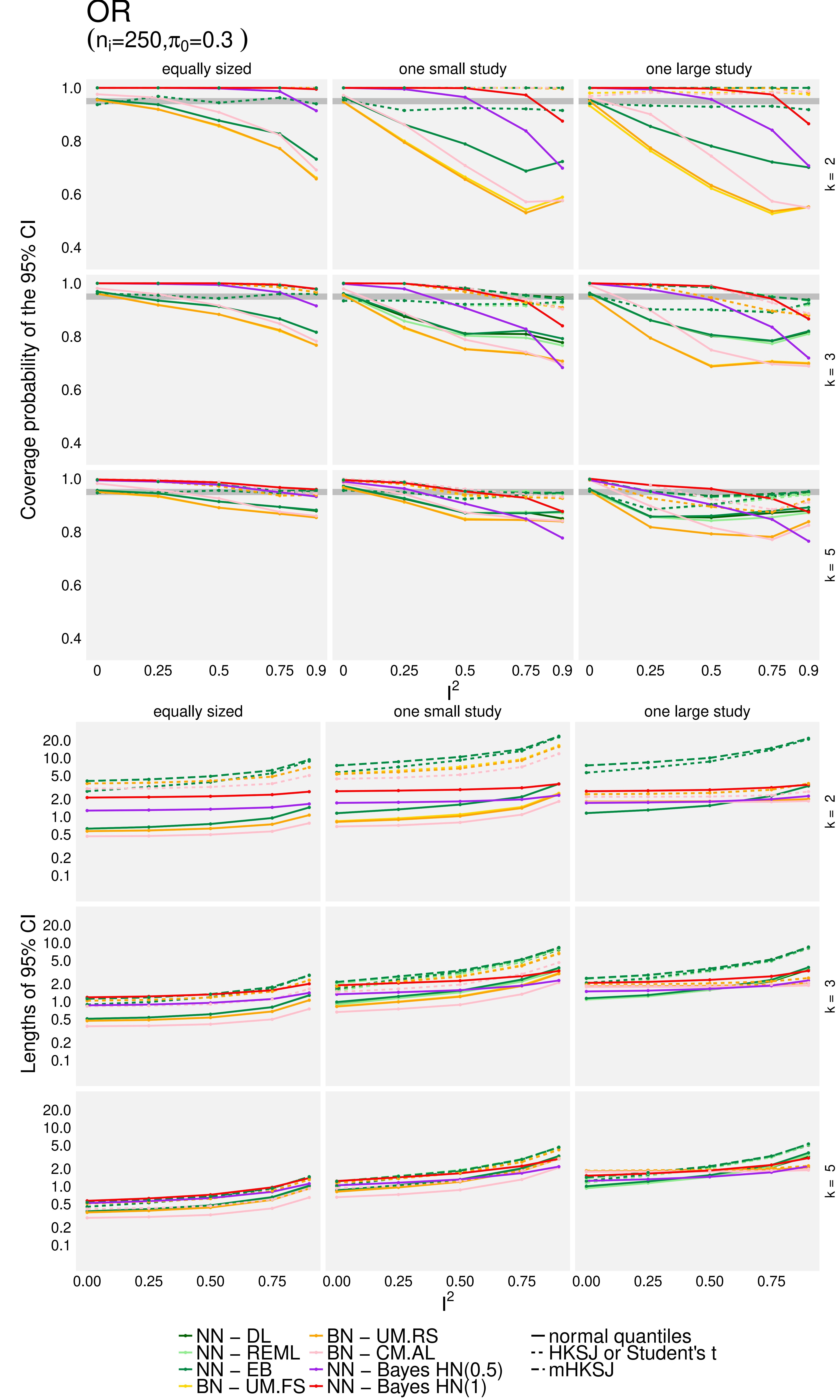

OR
(n_i=250,π_0=0.3)
equally sized
one small study
one large study
Coverage probability of the 95% CI
1.0
0.8
0.6
0.4
k = 2
k = 3
k = 5
0
0.25
0.5
0.75
0.9
I^2
Lengths of 95% CI
20.0
10.0
5.0
2.0
1.0
0.5
0.2
0.1
0.00
0.25
0.50
0.75
NN – DL
NN – REML
NN – EB
BN – UM.FS
BN – UM.RS
BN – CM.AL
NN – Bayes HN(0.5)
NN – Bayes HN(1)
normal quantiles
HKSJ or Student's t
mHKSJ

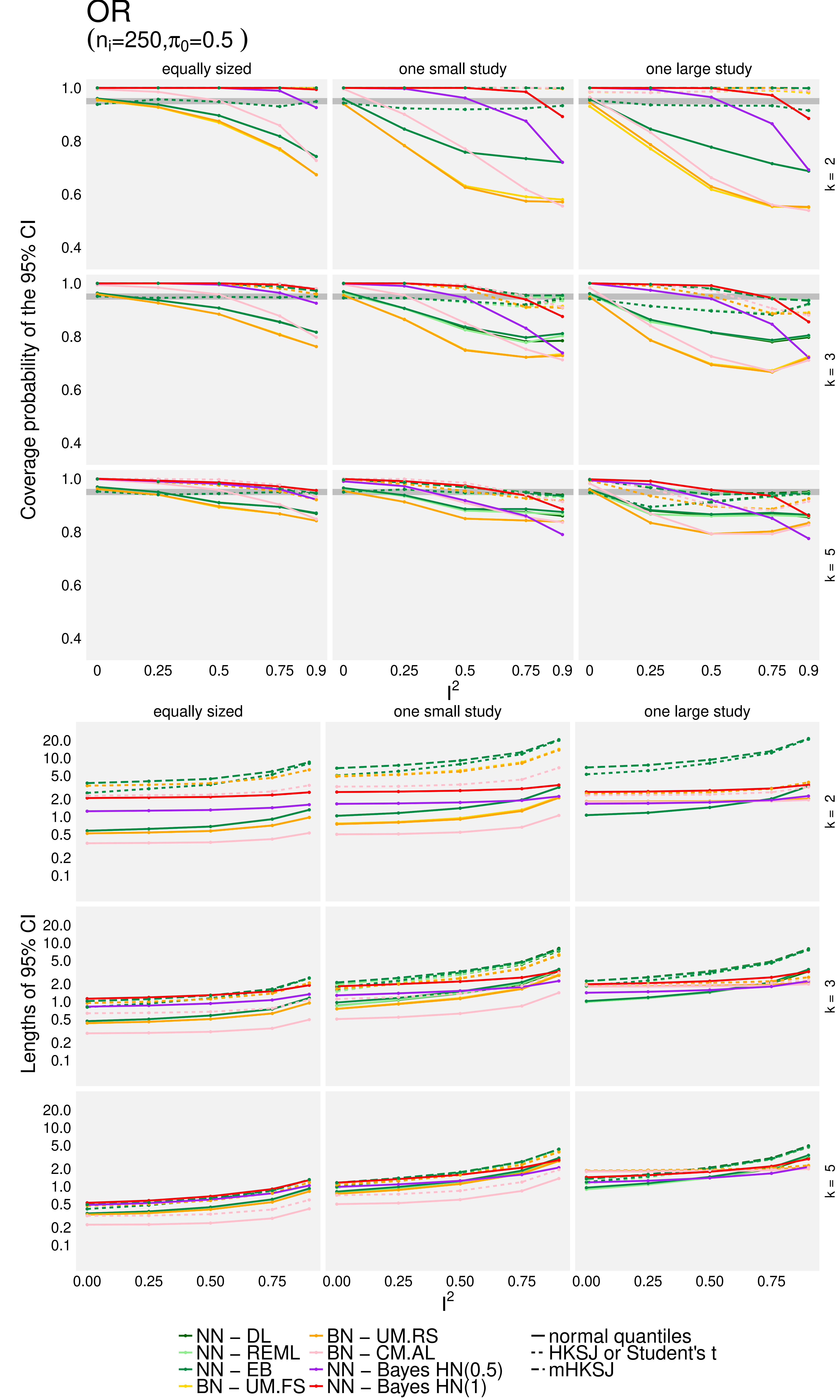
OR
(n_i=250,π_0=0.5)
equally sized
one small study
one large study
Coverage probability of the 95% CI
Lengths of 95% CI
k = 2
k = 3
k = 5
I²
NN – DL
NN – REML
NN – EB
BN – UM.FS
BN – UM.RS
BN – CM.AL
NN – Bayes HN(0.5)
NN – Bayes HN(1)
normal quantiles
HKSJ or Student's t
mHKSJ

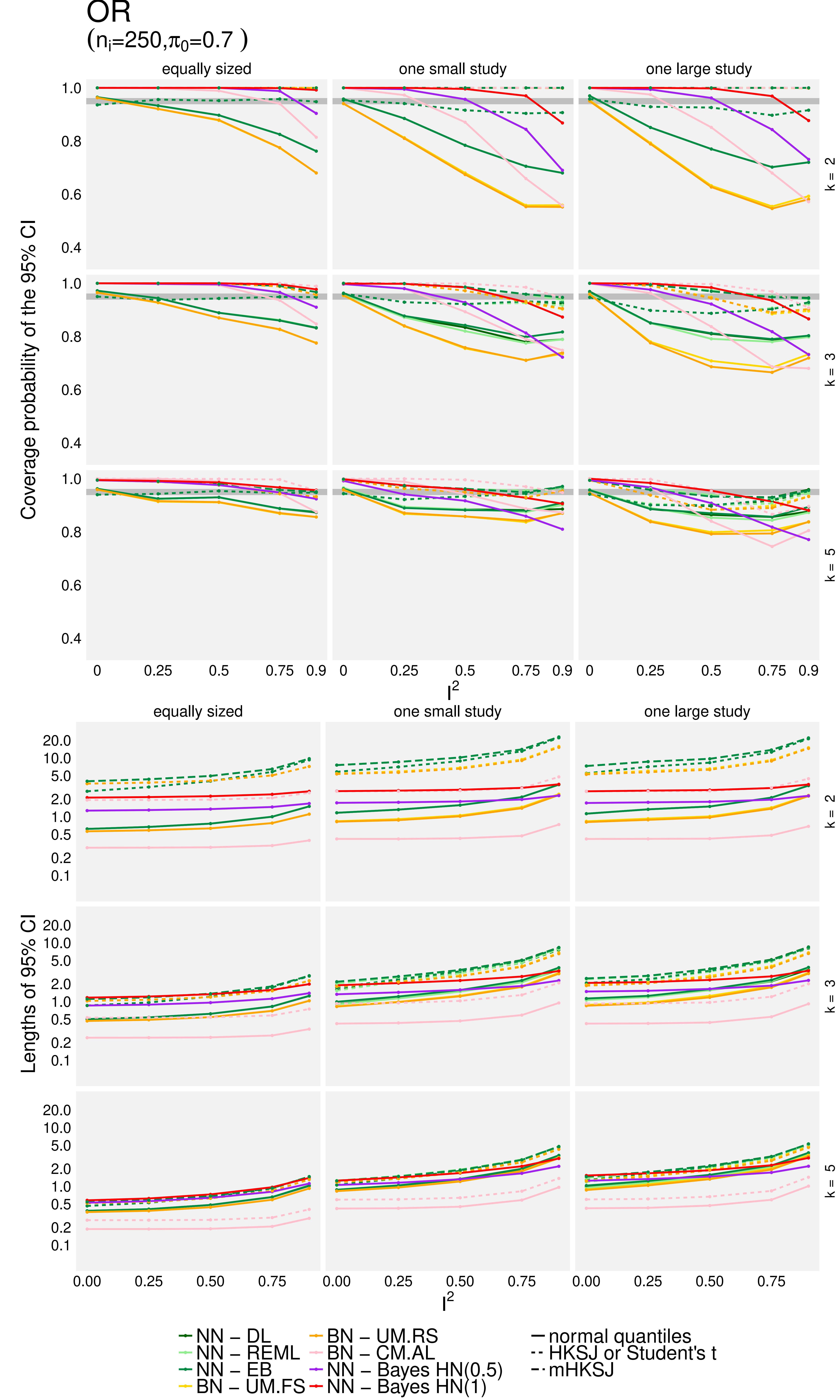

OR
(n_i=250,π_0=0.7)
equally sized
one small study
one large study
Coverage probability of the 95% CI
Lengths of 95% CI
k = 2
k = 3
k = 5
I^2
NN – DL
NN – REML
NN – EB
BN – UM.FS
BN – UM.RS
BN – CM.AL
NN – Bayes HN(0.5)
NN – Bayes HN(1)
normal quantiles
HKSJ or Student's t
mHKSJ

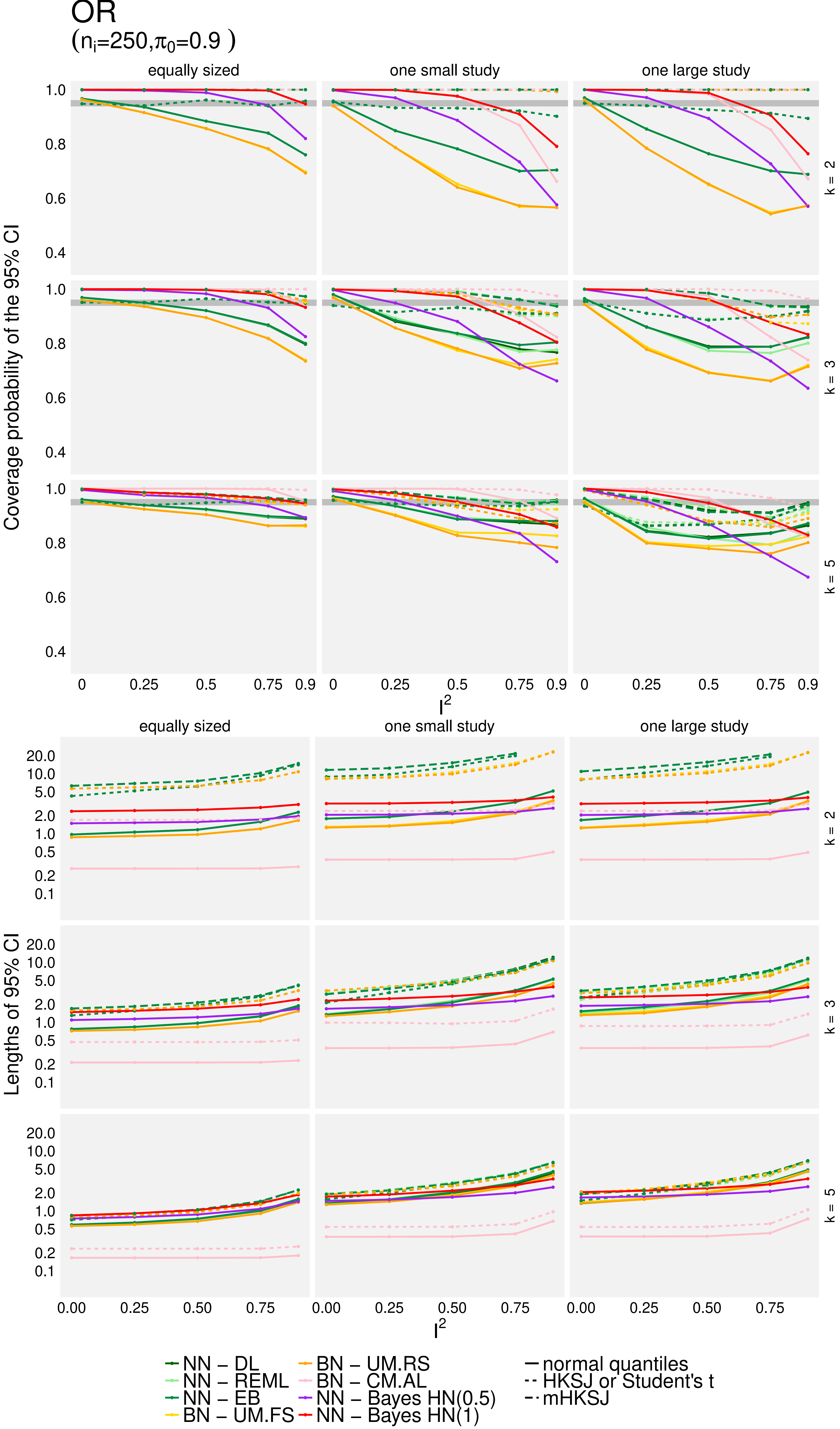
OR
(n_i=250,π_0=0.9)
equally sized
one small study
one large study
Coverage probability of the 95% CI
1.0
0.8
0.6
0.4
k = 2
k = 3
k = 5
0
0.25
0.5
0.75
0.9
I2
Lengths of 95% CI
20.0
10.0
5.0
2.0
1.0
0.5
0.2
0.1
0.00
0.25
0.50
0.75
NN – DL
NN – REML
NN – EB
BN – UM.FS
BN – UM.RS
BN – CM.AL
NN – Bayes HN(0.5)
NN – Bayes HN(1)
normal quantiles
HKSJ or Student's t
mHKSJ

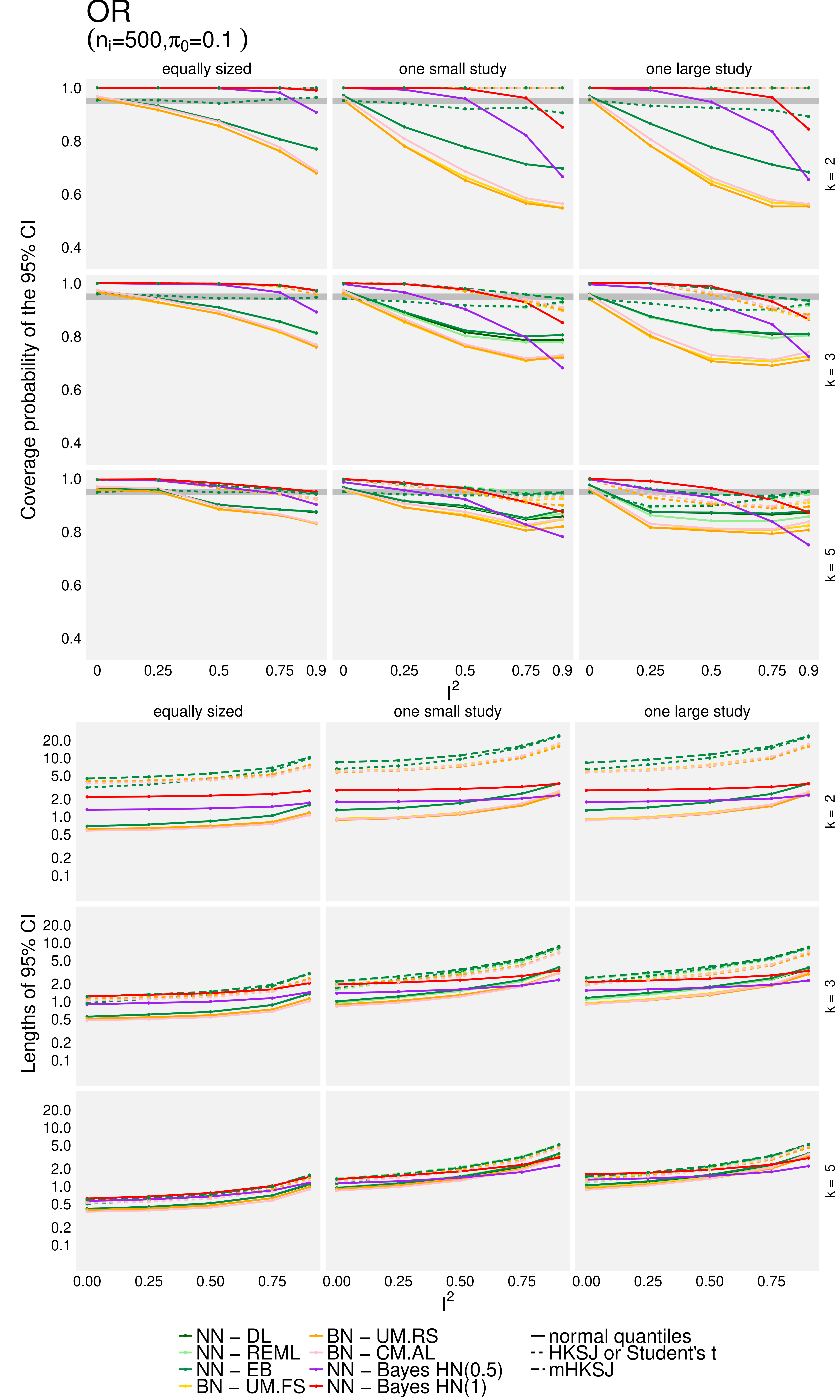

OR
(n_i=500,π_0=0.1)
equally sized
one small study
one large study
Coverage probability of the 95% CI
1.0
0.8
0.6
0.4
k = 2
k = 3
k = 5
0
0.25
0.5
0.75
0.9
I^2
Lengths of 95% CI
20.0
10.0
5.0
2.0
1.0
0.5
0.2
0.1
0.00
0.25
0.50
0.75
NN – DL
NN – REML
NN – EB
BN – UM.FS
BN – UM.RS
BN – CM.AL
NN – Bayes HN(0.5)
NN – Bayes HN(1)
normal quantiles
HKSJ or Student's t
mHKSJ

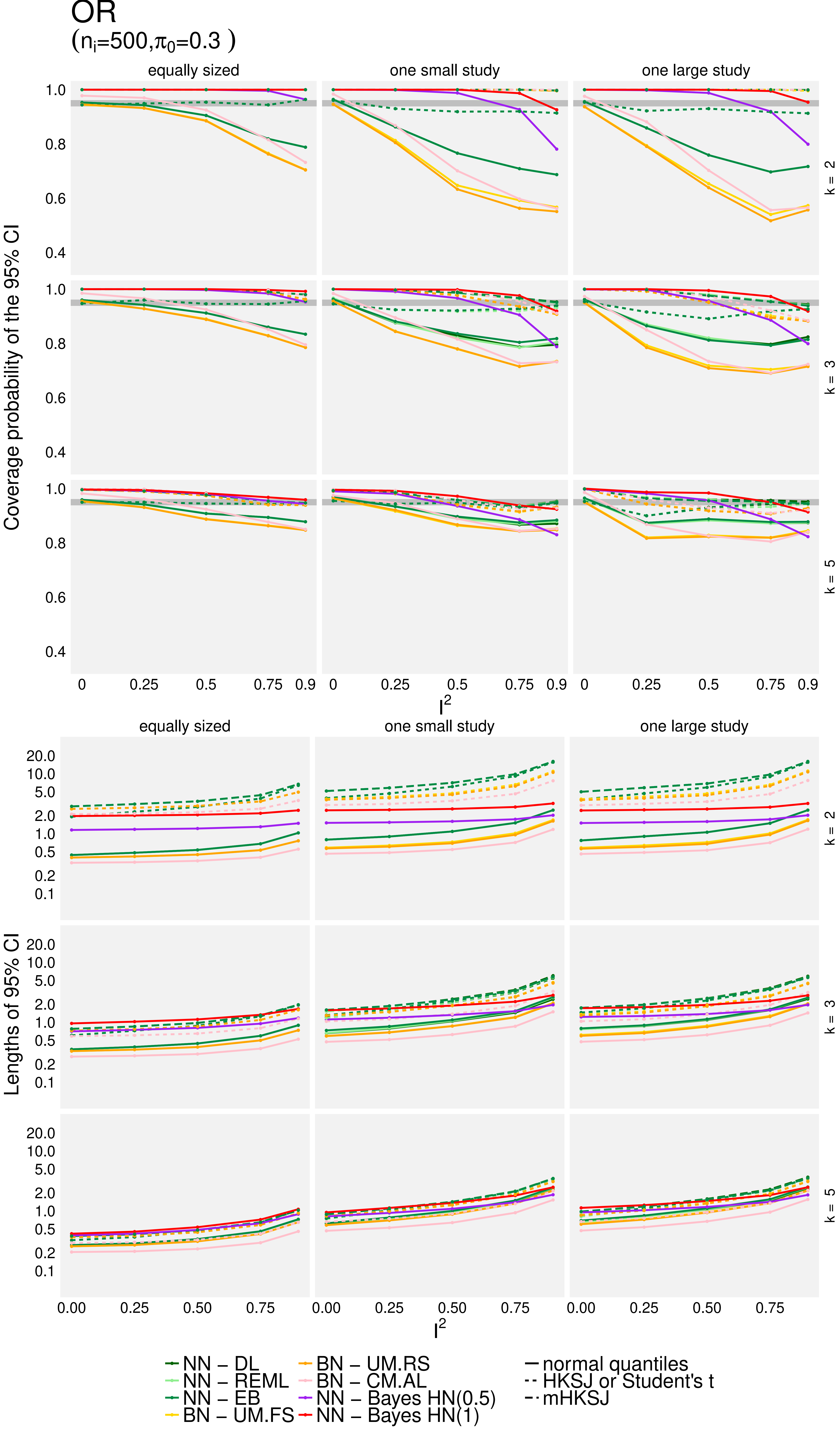
OR
(n_i=500,π_0=0.3)
equally sized
one small study
one large study
Coverage probability of the 95% CI
k = 2
k = 3
k = 5
I^2
Lengths of 95% CI
NN – DL
NN – REML
NN – EB
BN – UM.FS
BN – UM.RS
BN – CM.AL
NN – Bayes HN(0.5)
NN – Bayes HN(1)
normal quantiles
HKSJ or Student's t
mHKSJ

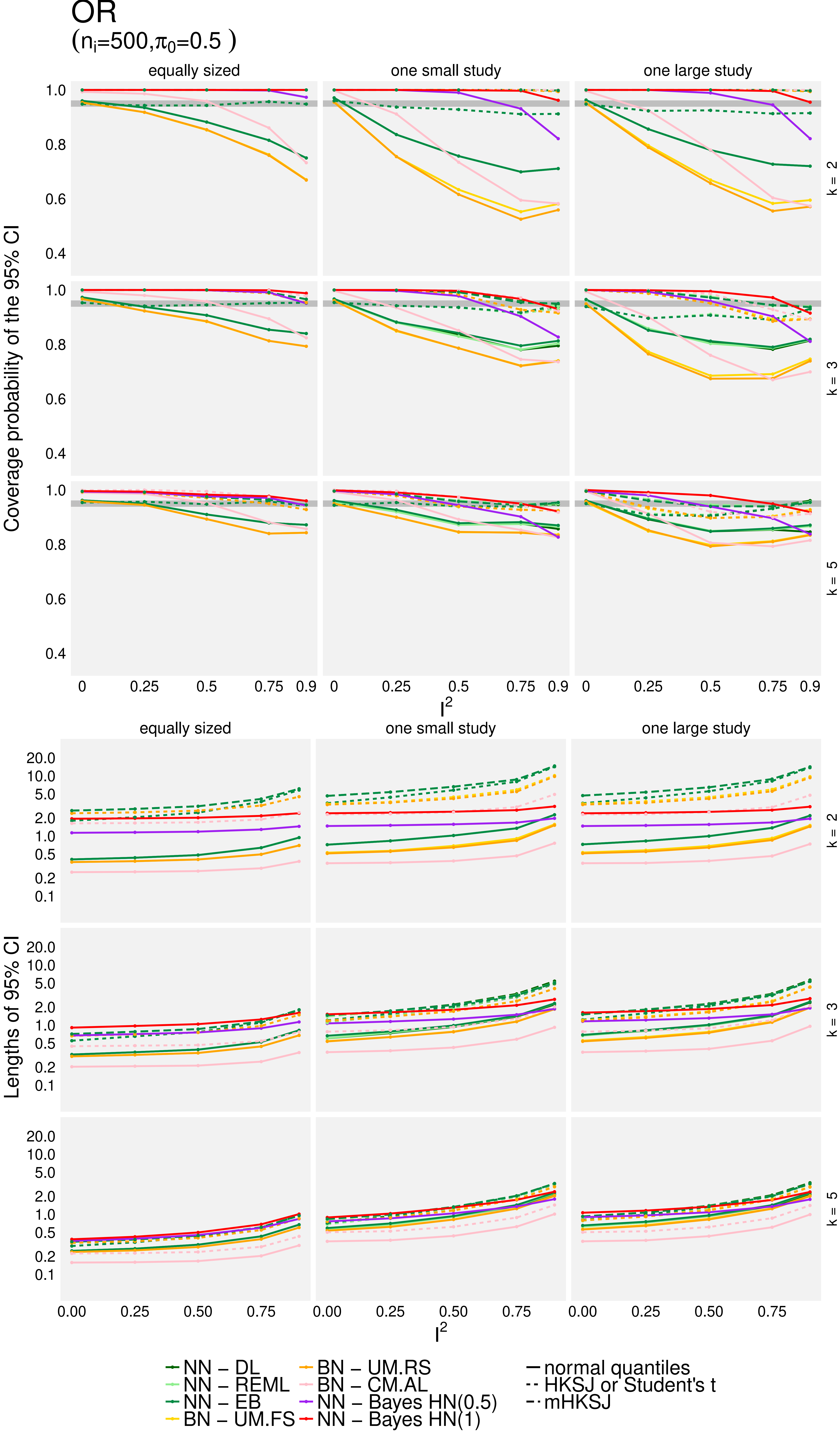

OR
(n_i=500,π_0=0.5)
equally sized
one small study
one large study
Coverage probability of the 95% CI
k = 2
k = 3
k = 5
I^2
Lengths of 95% CI
NN – DL
NN – REML
NN – EB
BN – UM.FS
BN – UM.RS
BN – CM.AL
NN – Bayes HN(0.5)
NN – Bayes HN(1)
normal quantiles
HKSJ or Student's t
mHKSJ

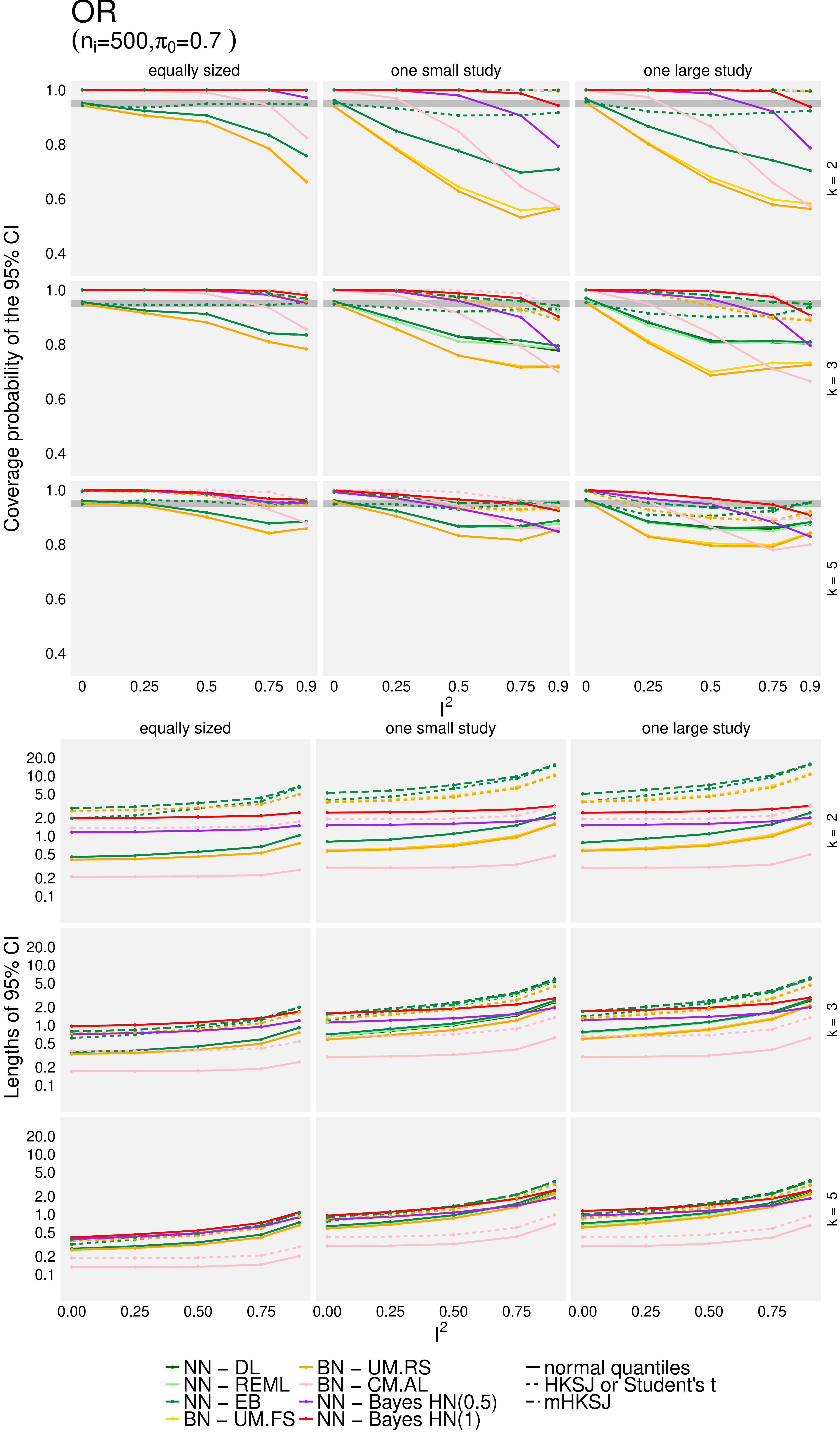

OR
(n_i=500,π_0=0.7)
equally sized
one small study
one large study
Coverage probability of the 95% CI
1.0
0.8
0.6
0.4
k = 2
k = 3
k = 5
0
0.25
0.5
0.75
0.9
I2
Lengths of 95% CI
20.0
10.0
5.0
2.0
1.0
0.5
0.2
0.1
0.00
0.25
0.50
0.75
NN – DL
NN – REML
NN – EB
BN – UM.FS
BN – UM.RS
BN – CM.AL
NN – Bayes HN(0.5)
NN – Bayes HN(1)
normal quantiles
HKSJ or Student's t
mHKSJ

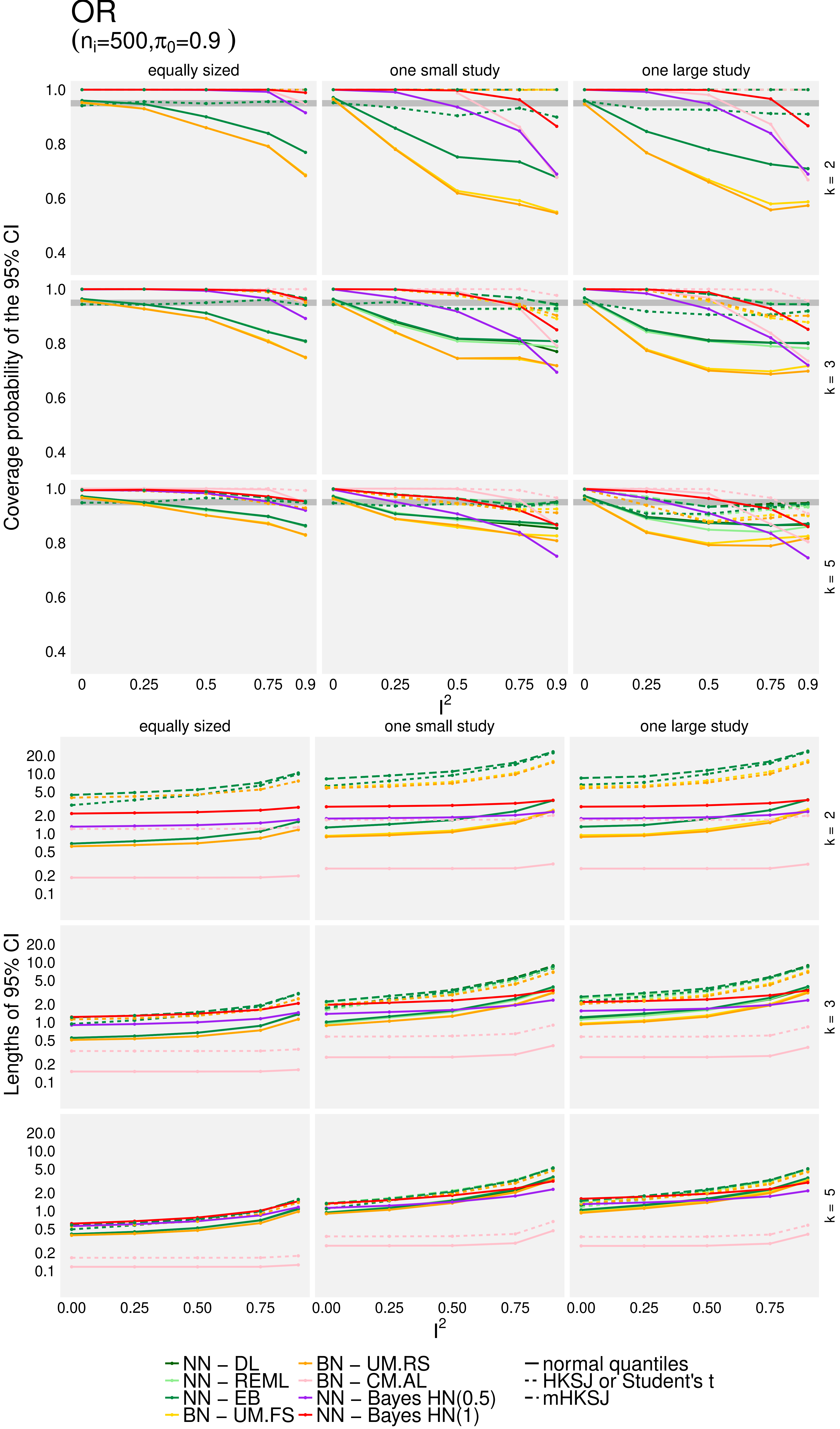
OR
(n_i=500,π_0=0.9)
equally sized
one small study
one large study
Coverage probability of the 95% CI
k = 2
k = 3
k = 5
I^2
Lengths of 95% CI
NN – DL
NN – REML
NN – EB
BN – UM.FS
BN – UM.RS
BN – CM.AL
NN – Bayes HN(0.5)
NN – Bayes HN(1)
normal quantiles
HKSJ or Student's t
mHKSJ

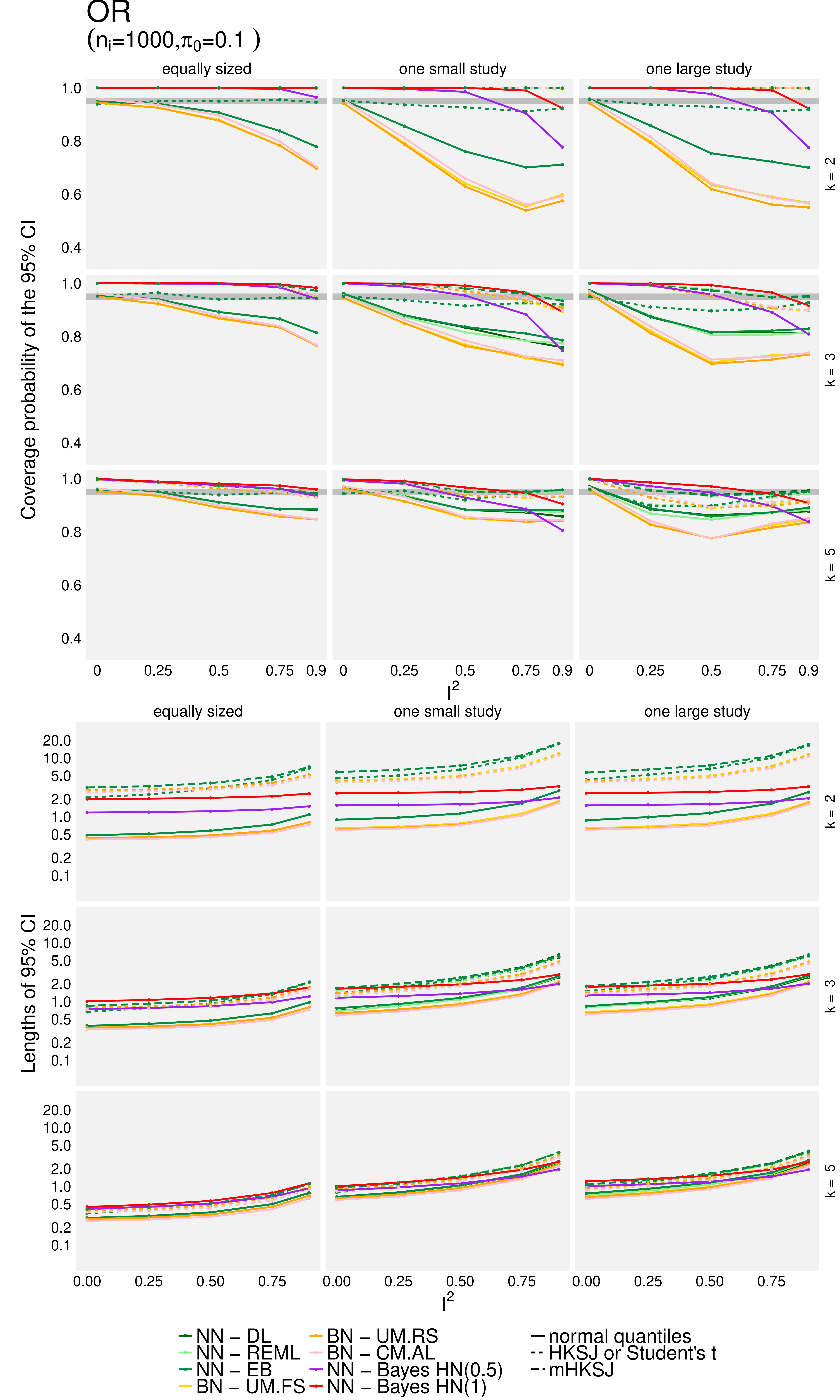

OR
(n_i=1000,π_0=0.1)
equally sized
one small study
one large study
Coverage probability of the 95% CI
1.0
0.8
0.6
0.4
k = 2
k = 3
k = 5
0
0.25
0.5
0.75
0.9
I^2
Lengths of 95% CI
20.0
10.0
5.0
2.0
1.0
0.5
0.2
0.1
0.00
0.25
0.50
0.75
NN – DL
NN – REML
NN – EB
BN – UM.FS
BN – UM.RS
BN – CM.AL
NN – Bayes HN(0.5)
NN – Bayes HN(1)
normal quantiles
HKSJ or Student's t
mHKSJ

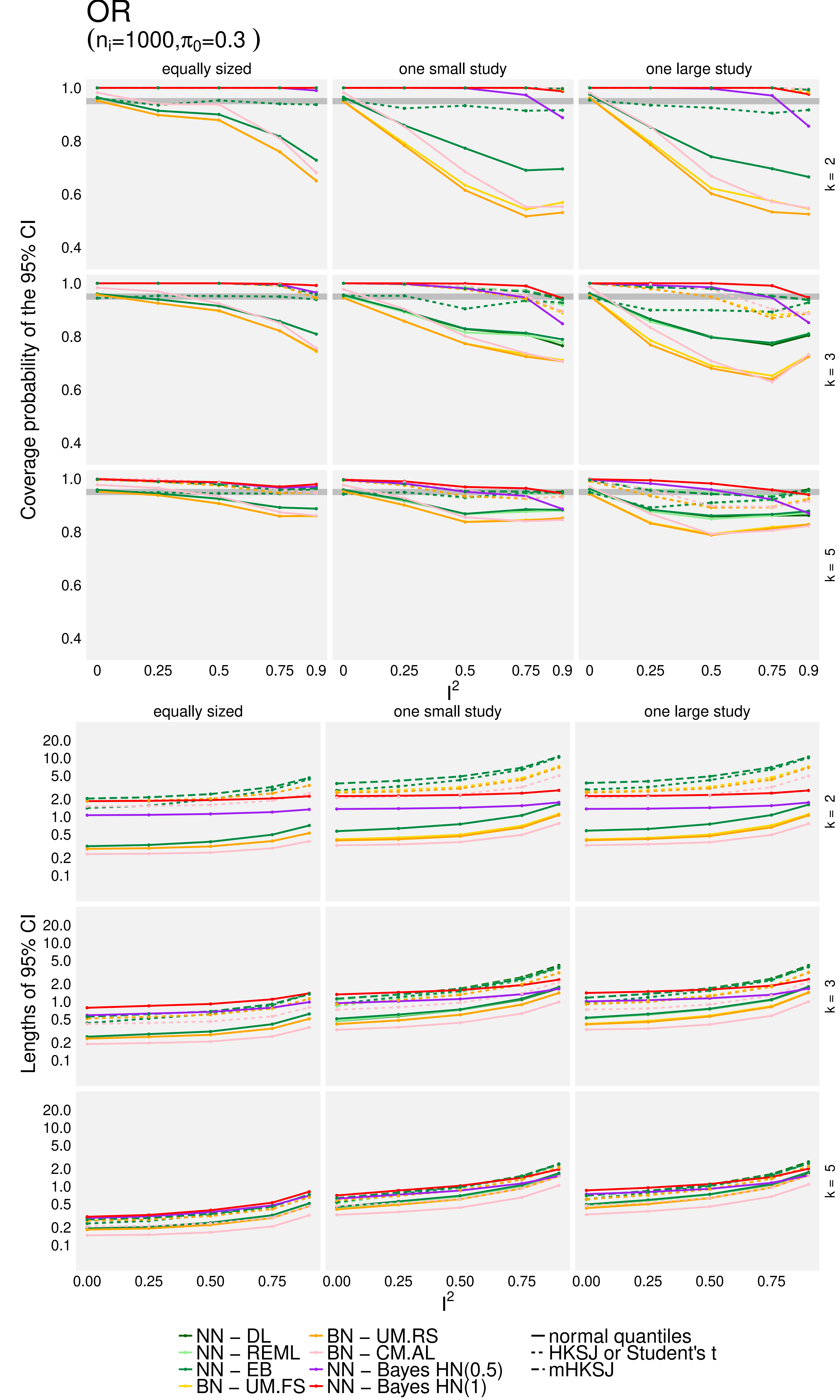

OR
(n_i=1000,π_0=0.3)
equally sized
one small study
one large study
Coverage probability of the 95% CI
k = 2
k = 3
k = 5
I^2
Lengths of 95% CI
NN – DL
NN – REML
NN – EB
BN – UM.FS
BN – UM.RS
BN – CM.AL
NN – Bayes HN(0.5)
NN – Bayes HN(1)
normal quantiles
HKSJ or Student's t
mHKSJ

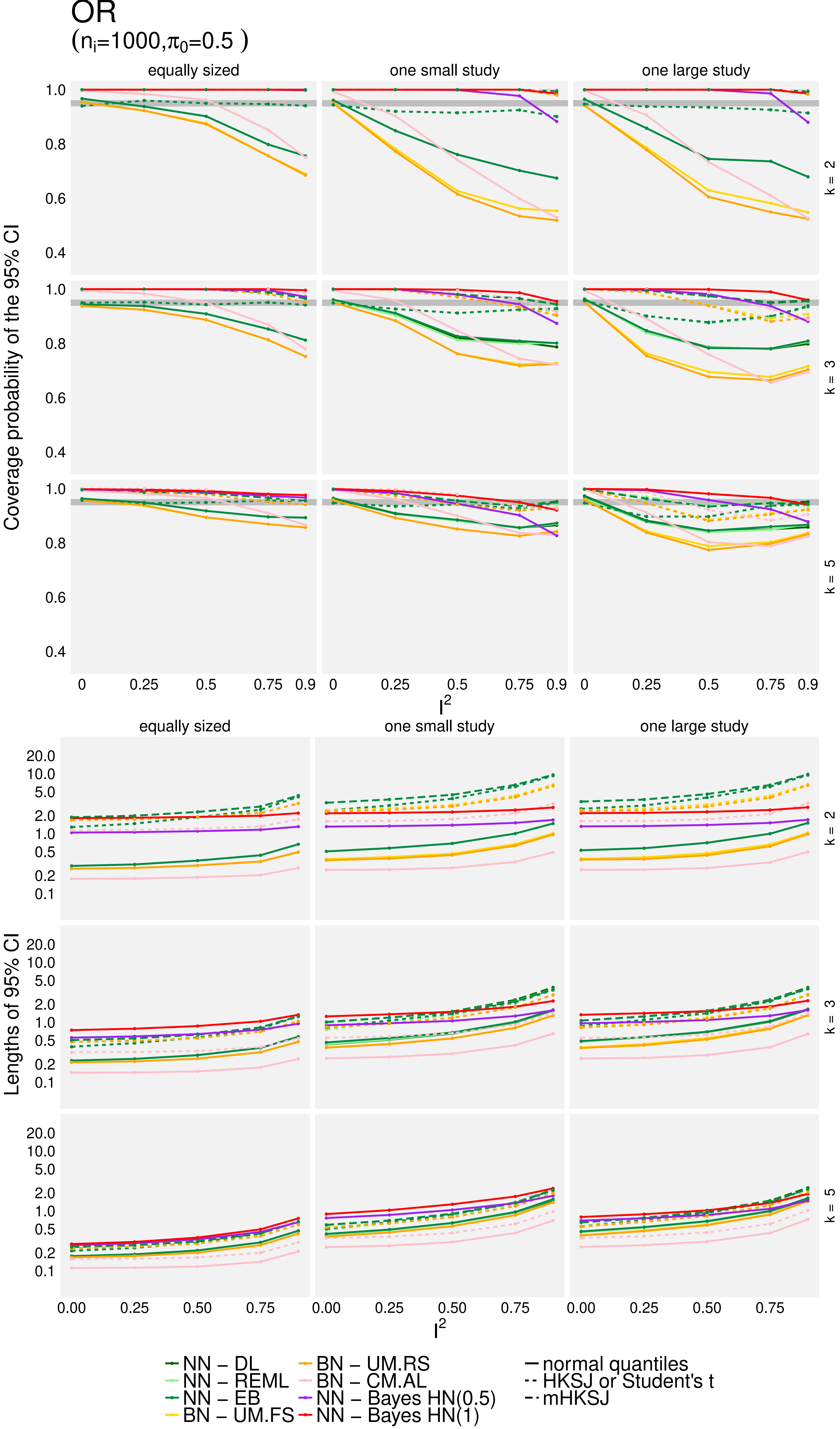
OR
(n_i=1000,π_0=0.5)
equally sized
one small study
one large study
Coverage probability of the 95% CI
k = 2
k = 3
k = 5
I^2
Lengths of 95% CI
NN – DL
NN – REML
NN – EB
BN – UM.FS
BN – UM.RS
BN – CM.AL
NN – Bayes HN(0.5)
NN – Bayes HN(1)
normal quantiles
HKSJ or Student's t
mHKSJ

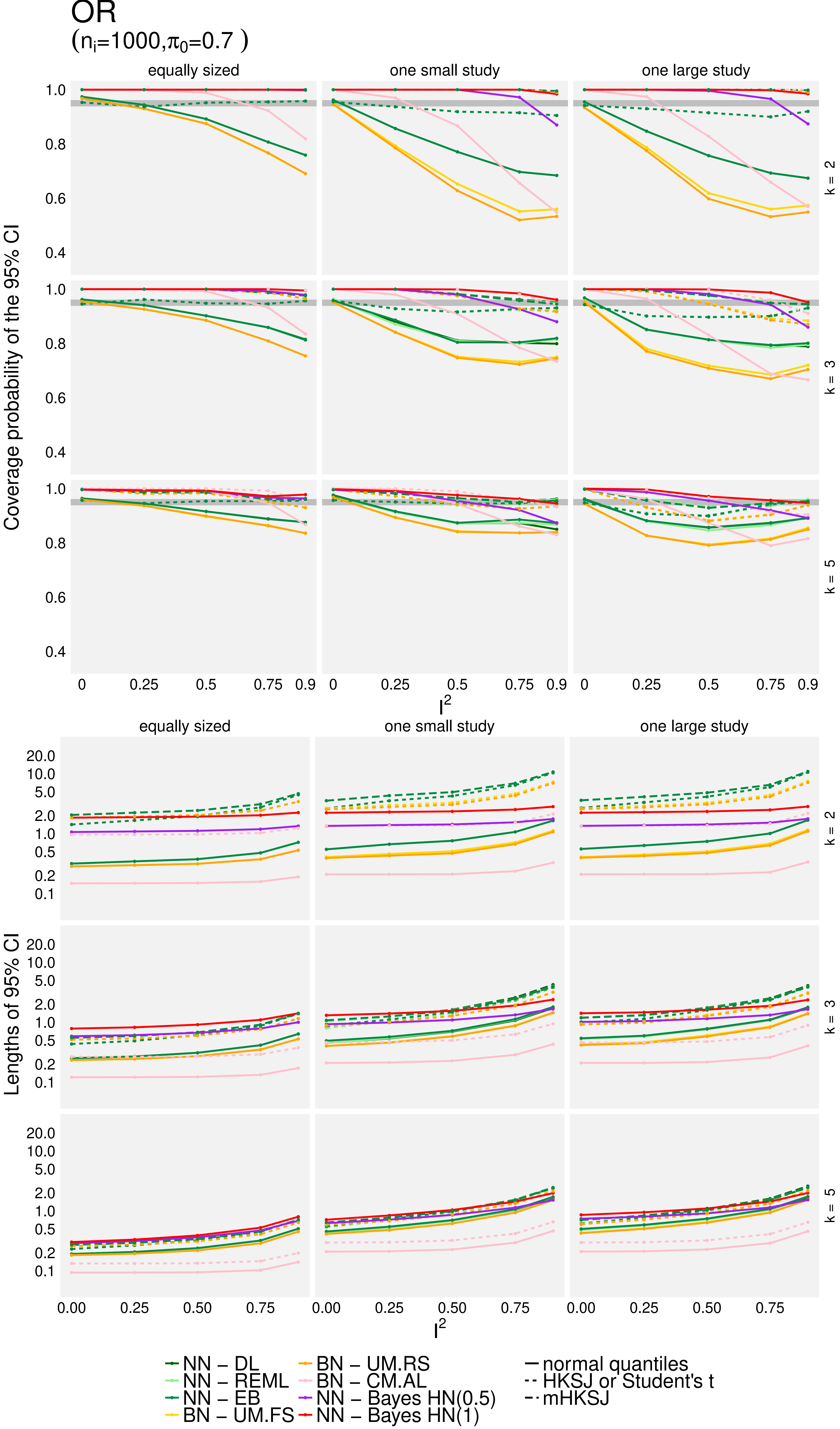

OR
(n_i=1000,π_0=0.7)
equally sized
one small study
one large study
Coverage probability of the 95% CI
k = 2
k = 3
k = 5
I^2
Lengths of 95% CI
NN – DL
NN – REML
NN – EB
BN – UM.FS
BN – UM.RS
BN – CM.AL
NN – Bayes HN(0.5)
NN – Bayes HN(1)
normal quantiles
HKSJ or Student's t
mHKSJ

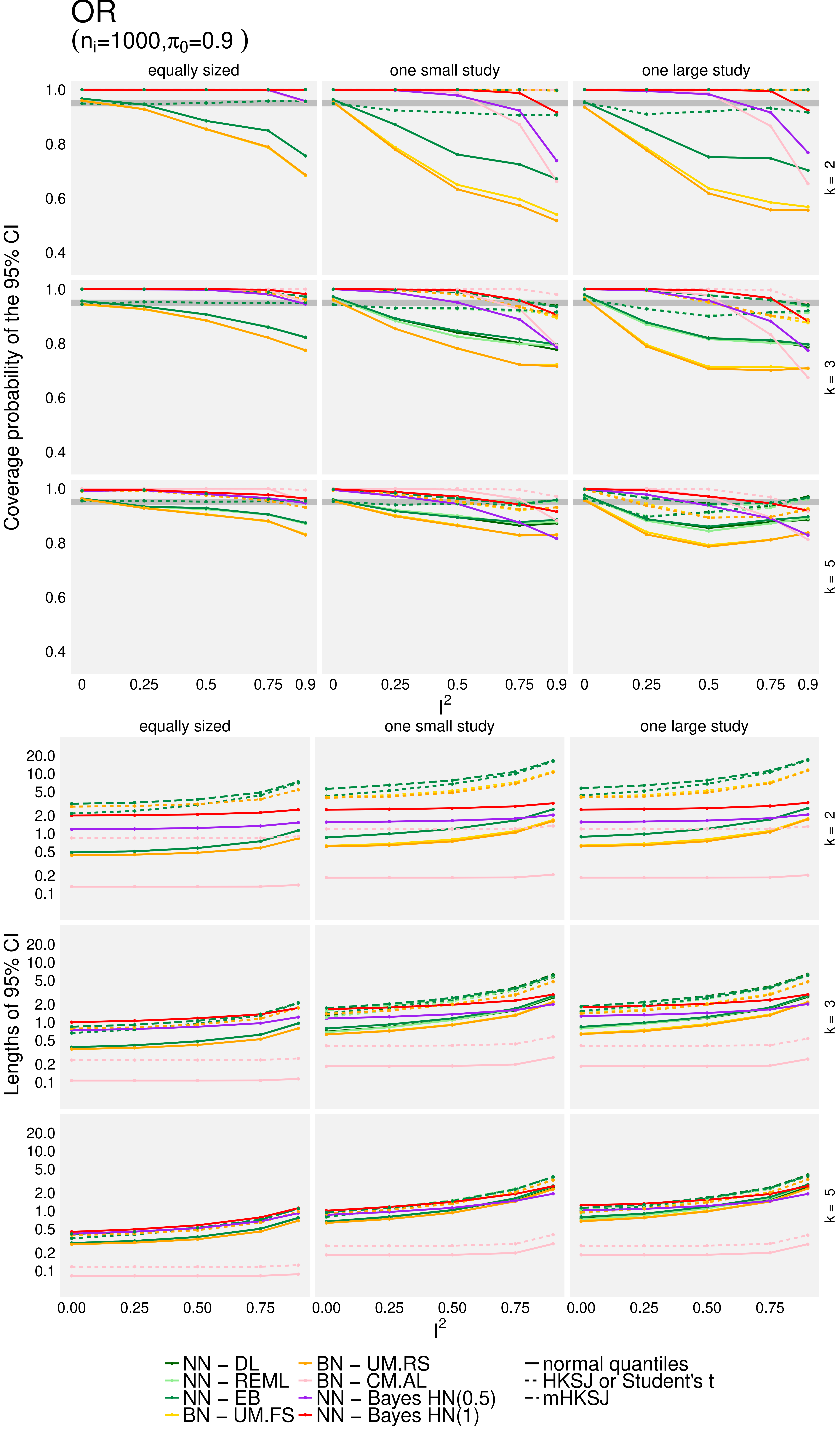

OR
(n_i=1000,π_0=0.9)
equally sized
one small study
one large study
Coverage probability of the 95% CI
k = 2
k = 3
k = 5
I^2
Lengths of 95% CI
NN – DL
NN – REML
NN – EB
BN – UM.FS
BN – UM.RS
BN – CM.AL
NN – Bayes HN(0.5)
NN – Bayes HN(1)
normal quantiles
HKSJ or Student's t
mHKSJ
